# Synthesis of 2-Amino-4, 5-Diarylthiazole Derivatives and Evaluation of Their Anti-*Candida* Albicans Activity

**DOI:** 10.3390/molecules30071643

**Published:** 2025-04-07

**Authors:** Dongmei Gao, Lele Shi, Yuhang Huang, Yingmei Lv, Xuan Yang, Zhenting Du

**Affiliations:** 1 Yangling Vocational & Technical College, Yangling 712100, China; 2 School of Chemistry and Pharmacy, Northwest A & F University, Yangling 712100, China; shilele23@163.com (L.S.); 15621485160@163.com (Y.H.); lvyingmei1213@163.com (Y.L.); 13980307830@163.com (X.Y.)

**Keywords:** 2-Amino-4, 5-diarylthiazole, anti-*Candida albicans* activity, molecular docking

## Abstract

The thiazole heterocycle is one of the most common moieties found in various drugs. Using 2-aminothiazole as the core structure, the amino group was functionalized with an amide. As a result, 30 trisubstituted 2-amino-4, 5-diarylthiazole derivatives were synthesized, with different substitutions introduced at the C2, C4, and C5 positions. The anti-*Candida albicans* biological activities of these synthetic compounds on five kinds of *Candida albicans* at different concentrations were detected by the microdilution method. In the first round, four derivatives of 2-amino-4, 5-diarylthiazole exhibited moderate anti-*Candida albicans* activity. Among them, **4a8** was chosen to be subjected to a demethylation process. Thus, **5a8** was synthesized successfully, giving anti-*Candida albicans* activity (MIC_80_ = 9 μM) similar to that of a typical antifungal drug, fluconazole. To understand the mechanism of anti-*Candida albicans*, molecular docking of the most active **5a8** against four target proteins of anti-*Candida albicans,* such as glutamine-fructose-6-phosphoamidamitransferase (GFAT), protein kinase (Yck2), heat-shock protein 90 (Hsp90), and lanosterol 14a-demethylase (CYP51) was carried out. Our research will provide an experimental basis and theoretical guidance for the further design of a new aminothiazole-leading pharmaceutical molecule.

## 1. Introduction

The incidence of invasive fungal infections (IFIs) has risen significantly, posing a serious clinical threat, largely due to the growing number of immunocompromised individuals worldwide [1,2,3]. While various factors contribute to this increase, a primary cause is the alteration of hosts’ immune systems, often a result of receiving solid organ or hematopoietic stem cell transplants, or the use of immune-modulating treatments such as tumor necrosis factor antagonists to manage chronic inflammatory conditions [4]. Among these infections, Candidiasis has emerged as one of the most frequent fungal diseases observed in immunosuppressed patients over the past two decades [5,6,7]. Such individuals are highly vulnerable to opportunistic infections, which are influenced by their specific health statuses. Infections caused by *Candida albicans* are particularly common in immunocompromised populations, such as those who have undergone long-term or heavy usages of broad-spectrum antibiotics, hormones, or immunosuppressive agents [8], or those with severe underlying conditions or history of organ transplant surgeries [9]. Currently, antifungal medications are the main therapeutic approach for deep fungal infections, but two significant challenges persist. First, prolonged use of antifungal drugs often leads to the development of drug resistance [10,11,12]. Second, the widespread emergence of drug-resistant bacteria in clinical settings has rendered antibiotic resistance a growing public health concern [13]. This has heightened the urgency for developing new antifungal agents or innovative drug delivery systems to address deep fungal infections [14,15,16].

**Figure 1:**
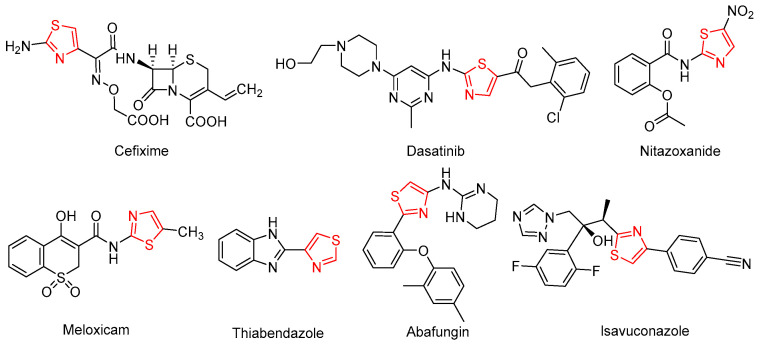
Structures of commercial drugs bearing thiazole.

Thiazole heterocycles represent a common structural motif in various biologically active compounds [17]. Numerous commercial drugs featuring the thiazole group (Figure 1), such as cefixime [18], dasatinib [19], nitazoxanide [20], meloxicam [21], abafungin [22] and isavuconazole [23], are widely employed in clinical antifungal treatments [24]. In the agricultural sector, thiabendazole [25,26] has been used as an antifungal agent against plant pathogens since Merck patented it in 1962. Bikobo and colleagues designed and synthesized a series of 2-phenylaminothiazole derivatives, evaluating their antifungal activity against *Candida albicans* and *Candida krusei* [27], and identified a promising lead compound with superior efficacy compared to fluconazole. Lino et al. synthesized novel thiazole derivatives with hydrazone groups and assessed their activity against seven fungi, including *Candida albicans*, with results indicating that the thiazole ring is crucial for antifungal effectiveness [28]. Kamat et al. developed pyridine-thiazole hydrazide compounds and evaluated their antibacterial and anti-inflammatory properties, finding that three of these compounds exhibited antifungal activity comparable to fluconazole and nystatin against four fungi, including *Candida albicans* [29].

Arora et al. obtained novel 2,4-disubstituted thiazole derivatives with anti-*Candida albicans* activity through computer-aided drug design (CADD) using the GFAT protein as a template, which laid a theoretical foundation for the study of the antifungal function of thiazole derivatives [30]. Pricopie et al. designed and synthesized two thiazole derivatives with lipophilic substituents and evaluated their anti-*Candida* activity in vitro. They found that the newly synthesized compounds may interfere with the ergosterol biosynthesis pathway by inhibiting 14α-demethylase (CYP51), and the destruction of the fungal cell membrane was observed through staining [31]. Cowen’s group screened a library of 736 protein kinase inhibitors, and they found that the 2,3-arylpyrazolopyridine derivative GW461484A was capable of restoring caspofungin sensitivity and potentiating the activity of fluconazole against *Candida albicans*. Yck2, a member of the CK1 (casein kinase 1) family, was confirmed to be the molecular target of GW461484A [32]. Based on the aforementioned literature review, thiazole derivatives exhibit notable antifungal activity, with 2,4-disubstituted aminothiazole compounds demonstrating significant antibacterial activity against clinical fungi *Candida albicans*. However, to the best of our knowledge, trisubstituted thiazole compounds are seldom reported. Heat-shock protein 90 (Hsp90) is an essential molecular chaperone that is highly conserved among eukaryotes [33]. Based on these results, studying how medicine candidates interact with these proteins is quite reasonable. Obviously, it was proved that using these targets to explore new leading compounds saves labor.

**Figure 2:**
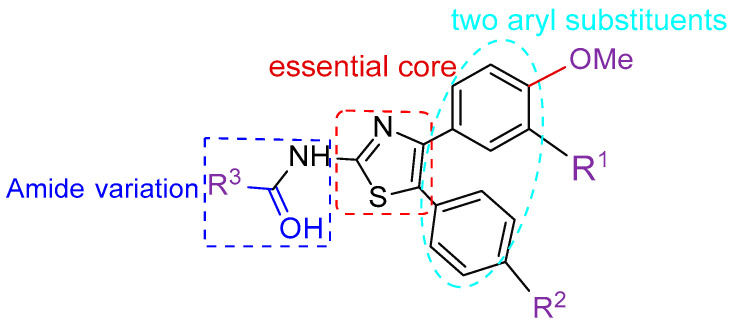
The design of molecules, including thiazole moiety.

Thus, we envisioned that the 2-aminothiazole moiety would be a promising substructure as developing new anti-*Candida albicans* derivatives. In addition, introducing an amide bond to 2-aminothiazole is a common strategy in structural modifications. A series of different substituted benzamide, heterocyclic amide, and aliphatic amide are introduced at the C2 position, while two substituted aryl groups are incorporated at the C4 and C5 positions (Figure 2).

## 2. Results and Discussion

### 2.1. Synthetic Results

**Figure 3:**
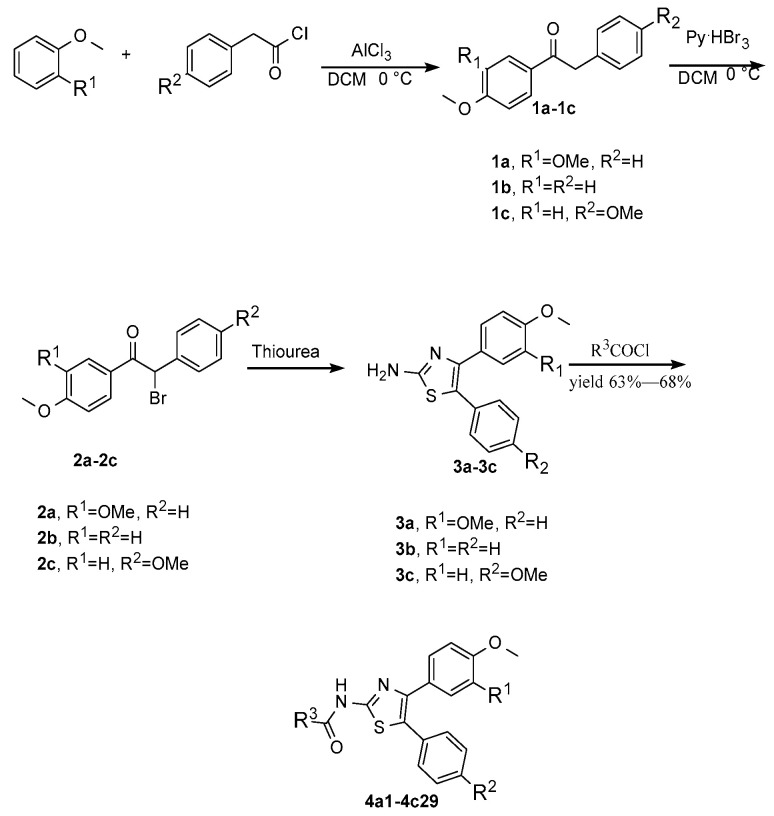
The synthesis procedure of the designed molecules.

The synthetic workflow is shown in Figure 3. The synthesis commenced at the two-aryl moiety through a Friedel–Crafts reaction to 1,2-diaryl-ethanone, similar to what has been reported in the literature [34]. At 0 °C, they were subjected to a bromination process using pyridinium tribromide, which is an alternative to liquid bromine; the corresponding bromo-ethanone derivatives **2a**–**2c** could be obtained in excellent yields. In ethanol, these derivatives reacted with thiourea at refluxed temperature, and the 2-NH_2_-thiazole heterocycles **3a**–**3c** were constructed. None of these synthetic intermediates were characterized fully, and after a conventional amide formation using acyl chloride in the presence of triethyl amine, the final derivatives were catered in good overall yields. A total of 29 compounds were synthesized, as shown in Table 1.

### 2.2. Antifungal Activity Against Candida Albicans

After synthesizing 29 compounds, the antifungal activities of the designed derivatives against five species of *Candida albicans* were evaluated in vitro. We used an FLC-sensitive strain (*Candida albicans* 186382) and five FLC-resistant species (4935, 5122, 5172, and 5272) to evaluate the antifungal activity of our synthetic compounds alone according to microdilution methods. Interestingly, compounds **4a1**, **4a8**, **4b19**, and **4b23** exhibited moderate activity (Table 2).

**Table 1:** The detailed information for designed compounds.

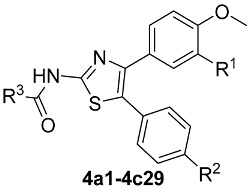	
**4a1**, R^1^ = OCH_3_, R^2^ = H, R^3^ = CH_3_CH_3_CH_2_-	**4a2**, R^1^ = OCH_3_, R^2^ = H, R^3^ = CH_3_(CH_2_)_3_CH_2_-
**4a3**, R^1^ = OCH_3_, R^2^ = H, R^3^ = CH_3_(CH_2_)_5_CH_2_-	**4a4**, R^1^ = OCH_3_, R^2^ = H, R^3^ = C_6_H_4_-
**4a5**, R^1^ = OCH_3_, R^2^ = H, R^3^ = PhCH_2_CH_2_-	**4a6**, R^1^ = OCH_3_, R^2^ = H, R^3^ = 2-Me-C_6_H_4_-
**4a7**, R^1^ = OCH_3_, R^2^ = H, R^3^ = 3-MeC_6_H_4_-	**4a8**, R^1^ = OCH_3_, R^2^ = H, R^3^ = 4-Me-C_6_H_4_-
**4a9**, R^1^ = OCH_3_, R^2^ = H, R^3^ = 2-MeO-C_6_H_4_-	**4a10**, R^1^ = OCH_3_, R^2^ = H, R^3^ = 4-MeO-C_6_H_4_-
**4a11**, R^1^ = OCH_3_, R^2^ = H, R^3^ = 2-F-C_6_H_4_-	**4a12**, R^1^ = OCH_3_, R^2^ = H, R^3^ = 3-F-C_6_H_4_-
**4a13**, R^1^ = OCH_3_, R^2^ = H, R^3^ = 3-Cl-C_6_H_4_-	**4a14**, R^1^ = OCH_3_, R^2^ = H, R^3^ = 4-Cl-C_6_H_4_-
**4a15**, R^1^ = OCH_3_, R^2^ = H, R^3^ = 4-I-C_6_H_4_-	**4a16**, R^1^ = OCH_3_, R^2^ = H, R^3^ = 4-CF_3_-C_6_H_4_-
**4a17**, R^1^ = OCH_3_, R^2^ = H, R^3^ = 2-thiophenyl-	**4a18**, R^1^ = OCH_3_, R^2^ = H, R^3^ = 6-Cl-nicotinoy-
**4b19**, R^1^ = R^2^ = H, R^3^ = 2-MeO-C_6_H_4_-	**4b20**, R^1^ = R^2^ = H, R^3^ = 3-Cl-C_6_H_4_-
**4b21**, R^1^ = R^2^ = H, R^3^ = 4-Cl-C_6_H_4_-	**4b22**, R^1^ = R^2^ = H, R^3^ = 6-Cl-nicotinoy-
**4b23**, R^1^ = R^2^ = H, R^3^ = 1-Naphthyl-	**4c24**, R^1^ = H, R^2^ = OCH_3_, R^3^ = 3-F-C_6_H_4_-
**4c25**, R^1^ = H, R^2^ =OCH_3_, R^3^ = 4-F-C_6_H_4_-	**4c26**, R^1^ = H, R^2^ = OCH_3_, R^3^ = 3-Cl-C_6_H_4_-
**4c27**, R^1^ = H, R^2^ = OCH_3_, R^3^ = 4-Cl-C_6_H_4_-	**4c28**, R^1^ = H, R^2^ = OCH_3_, R^3^ = 3-CF_3_-C_6_H_4_-
**4c29**, R^1^ = H, R^2^ = OCH_3_, R^3^ = 4-CF_3_-C_6_H_4_-	

**Figure 4:**
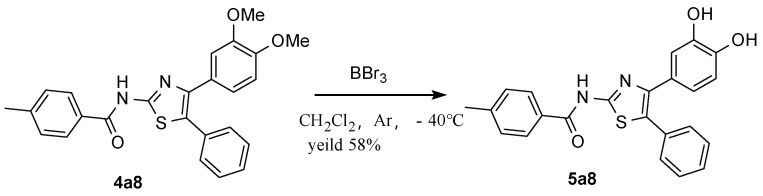
Synthesis of demethylated compound from **4a8**.

With these data on hand, to differentiate the activities between -OMe and -OH, the demethylated form of the most active **4a8** was obtained after it was subjected to a boron tribromide demethylation protocol [35] (Figure 4).

As shown in Table 3, after the demethylation, the corresponding compound showed excellent biological activity against *Candida albicans*. Regardless of whether the strain was sensitive or resistant, the activity was boosted remarkably in both. Even more, in this experimental condition, the biological activity was similar to that of the control, commercial fluconazole. It is possible that the two hydroxyl groups in **5a8** enhanced the solubility of the molecules and gave the best results. At the same time, the C2 position of the thiazole, aryl, especially 4-methyl phenyl, as in **5a8**, had the best activity.

### 2.3. Molecular Docking Against Different Proteins

The molecular docking method was used to predict the binding mode between the small molecule and the protein. The docking process was carried out using AutoDock Vina software(v1.1.2). Before docking, the crystal structure of *Candida albicans* glutamine-fructose-6-phosphate aminotransferase (GFAT), protein kinase (Yck2), lanosterol 14a-demethylase (CYP51), and heat-shock protein 90 (Hsp90) were obtained from the Protein Data Bank (PDB) (PDB ID, 2POC). The 3D structure of compound **5a8** was constructed using Chem3D, and energy-minimized with the MMFF94 force field. The protein was preprocessed using PyMol(V2.4.0a0) software, including hydrogen addition, the removal of water molecules, and the removal of non-ligand small molecules. A docking box was then defined to enclose compound **5a8** within the protein’s active pocket. Finally, the PDB format of both the small molecule and receptor protein was converted to PDBQT format using the AutoDockFR software(v1.0) suite. The docking was then performed, and the results were visualized and analyzed using PyMol.

#### 2.3.1. GFAT as the Target

Glutamine-fructose-6-phosphoamidamitransferase (GFAT) was found to be the key rate-limiting enzyme in the first step of chitin synthesis in the cell walls of fungi [30]. Inhibition of GAFT leads to the inability of the fungal cell wall to grow, resulting in the death of the entire fungus.

**Figure 5:**
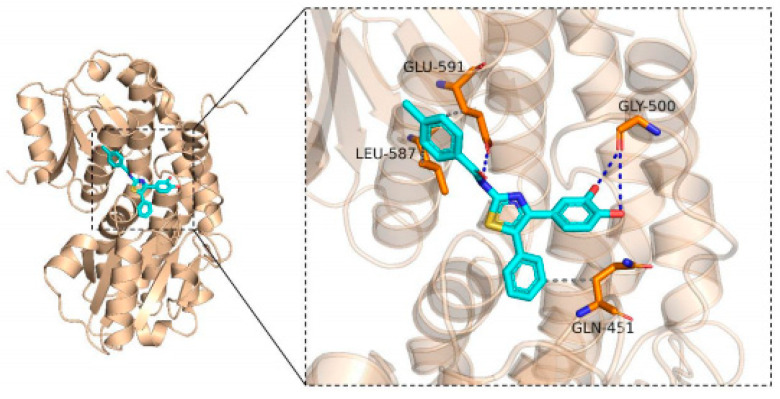
The binding mode of **5a8** and GFAT protein.

In Figure 5, the blue color represents the 3D structure of small molecule **5a8**, the orange color represents the protein GFAT structure, the blue dashed line represents hydrogen bonding, and the gray dashed line represents the hydrophobic interaction. Thus, we could see that compound **5a8** is embedded into the protein GFAT. It interacts with the residual of Gly-500 and Glu-591 by hydrogen bond. It also maintains hydrophobic interaction with Leu-587, Gln-451, and Gln-591. This reveals that the hydroxyl group is indispensable in the exhibition of biological activities. The docking score was −7.426 kcal/mol.

#### 2.3.2. CYP51 as Target

**Figure 6:**
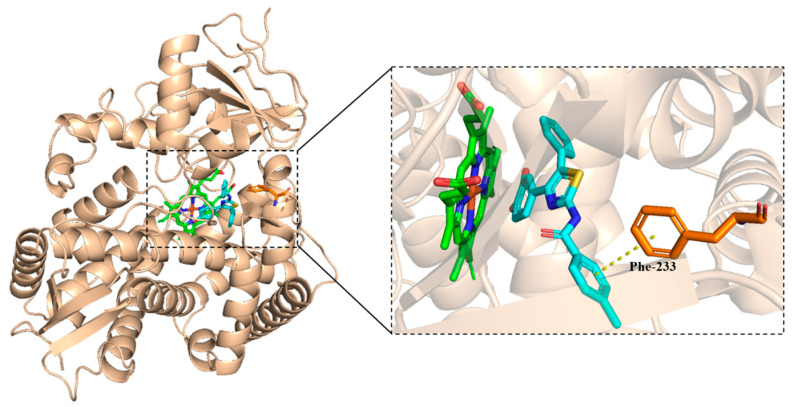
The binding mode of **5a8** and CYP51 protein.

There is some π-π stack between the methylphenyl group and Phe-233 in CYP51. However, the docking score was as high as −8.104 kcal/mol, as shown in Figure 6.

#### 2.3.3. Yck2 as Target

**Figure 7:**
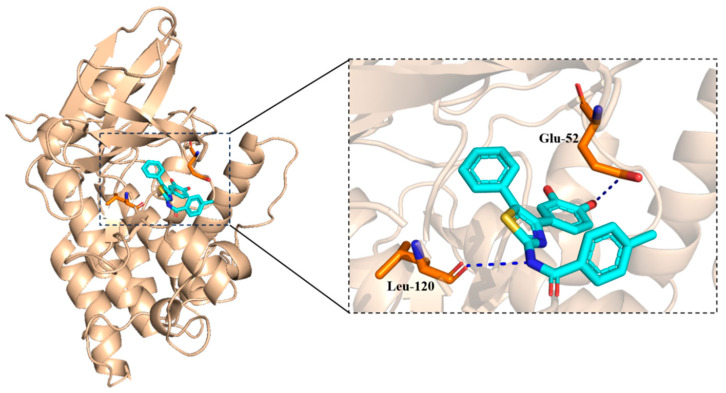
The binding mode of **5a8** and Yck2 protein.

As shown in Figure 7, two hydrogen bonds (blue dash line) were observed. The hydroxyl group seemed important in forming the hydrogen bonds. The docking score was −4.894 kcal/mol.

#### 2.3.4. Hsp90 as Target

**Figure 8:**
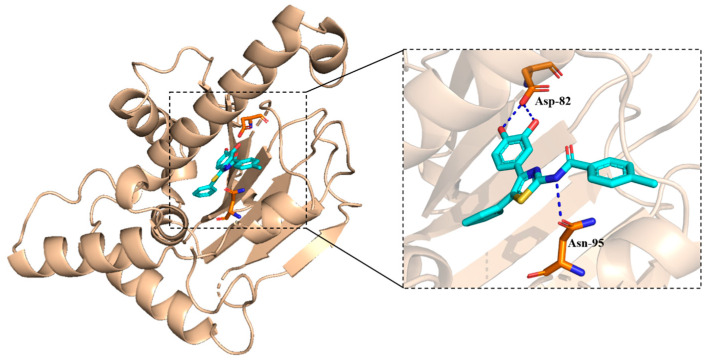
The binding mode of **5a8** and Hsp90 protein.

As shown in Figure 8, there were three main interactions between **5a8** and Hsp90. These interactions revealed that the hydroxyl group in **5a8** is crucial for biological activity. The docking score was −6.431 kcal/mol.

## 3. Materials and Methods

Melting points were determined using a WRS-1B melting point apparatus (Shanghai Precision & Scientific Instrument Co., Ltd., Shanghai, China); HRMS data were acquired on a Waters Xevo G2-S QTof mass spectrometer (Waters Corporation, Milford, MA, USA) with an electrospray ionization (ESI) source; and NMR spectra were recorded on Bruker Avance III 400 and 500 MHz spectrometers (Bruker Biospin, Rheinstetten, Germany) equipped with 5 mm inverse probes. The general procedures are as follows: Air- and moisture-sensitive reactions were performed in flame-dried round bottom flasks fitted with rubber septa under a positive argon pressure. Air- and moisture-sensitive liquids and solutions were transferred via a syringe or stainless-steel cannula. Organic solutions were concentrated by rotary evaporation below 35 °C at 20 mm Hg. Flash column chromatography was performed employing silica gel (230–400 mesh). Thin-layer chromatography (TLC) was performed using glass plates with silica gel F245, and eluted by PE:EA 20:1–5:1. HRMS was detected on a Waters G2-S qtof instrument. All chemicals were purchased from Chinese Chemical Company such as Aladdin, Leyan, Energy chemiccls etc.

### 3.1. 1-(3,4-Dimethoxyphenyl)-2-phenylethan-1-one ***1a***

To a 250 mL round-bottom flask, *o*-dimethoxybenzene (10.0 g, 72.38 mmol) in 100 mL of dichloromethane was added. Then, anhydrous aluminum chloride (14.5 g, 108.57 mmol) was added to the flask quickly; phenylacetyl chloride (12.5 mL, 94.09 mmol) was introduced into the reaction flask dropwise. The reaction was allowed to stir at 0 °C for two hours and was monitored by TLC. After the reaction was complete, it was quenched by pouring into ice water. After the removal of the reaction solvent under reduced pressure, the residual was extracted with ethyl acetate (100 mL × 3). The extracts were combined, dried over anhydrous sodium sulfate, filtered, and concentrated under reduced pressure. Compound **1a** was obtained in 78% yield after column chromatography.

The synthesis of **1b** and **1c** was similar to that of **1a**, and in the yields of 74% and 76%, respectively.

### 3.2. 2-Bromo-1-(3,4-dimethoxyphenyl)-2-phenylethan-1-one ***2a***

At first, the reaction glassware was equipped with an alkalic absorption beaker. To that, under an ice-water bath, a solution of **1a** (14.0 g, 54.62 mmol) in dichloromethane (100 mL) was added to pyridinium tribromide (19.0 g, 59.41 mmol), and the reaction mixture was allowed to stir for five hours at ambient temperature. The reaction process was monitored by TLC. After completion, it was quenched with sodium sulfite; the reaction mixture was extracted with ethyl acetate (100 mL × 3). The extracts were combined, dried over anhydrous sodium sulfate, filtered, and concentrated under reduced pressure. Compound **2a** was used without further purification.

The synthesis of **2b** and **2c** was similar to that of **1a**, with yields of 72% and 70%, respectively.

### 3.3. 4-(3,4-Dimethoxyphenyl)-5-phenylthiazol-2-amine ***3a***

To a 250 mL round-bottom flask, **2a** (15.0 g, 49.48 mmol) and 95% ethanol (100 mL) were added, before adding thiourea (4.1 g, 54.43 mmol), and the reaction mixture was heated to reflux for five hours. The reaction was monitored by TLC. After the reaction was complete, it was quenched by pouring into ice water. After the removal of the most reaction solvent under reduced pressure, the residual was basified to pH 7–8 using 10% NaOH, and then a large quantity of solid precipitated. It was filtered, collected, and dried. Compound **3a** was obtained in 70% yield after column chromatography using PE:EA 20:1–5:1 as eluents.

The synthesis of **3b** and **3c** was similar to that of **1a**, with yields of 72% and 70%, respectively.

### 3.4. N-(4-(3,4-dimethoxyphenyl)-5-phenylthiazol-2-yl)butyramide ***4a1***

To a solution of intermediate **3a** (0.3 g, 0.96 mmol) in 20 mL tetrahydrofuran, an appropriate amount of triethylamine and a small amount of 4-dimethylamino pyridine was added as catalysts. Finally, butyryl chloride (0.14 mL, 1.15 mmol) was introduced into the above-mentioned mixed system, and the reaction was allowed to stir at room temperature for about 10 h. The reaction was monitored by TLC until completion. The reaction mixture was poured into ice water, and the precipitate was collected and recrystallized in methanol. The target compound **4a1** was obtained with a 65% yield.

The synthesis of compounds **4a2–4c29** was carried out using a procedure analogous to that described for 4a1, yielding target products in 63–68% isolated yields. Comprehensive structural characterization data for all new compounds—including ^1^H NMR, ^13^C NMR, and HRMS spectra—are provided in the Supplementary Information.

### 3.5. N-(4-(3,4-dihydroxyphenyl)-5-phenylthiazol-2-yl)-4-methylbenzamide ***5a8***

To a solution of **4a8** (0.3 g, 0.72 mmol) reacting with 20 mL of dichloromethane in a 50 mL round-bottom flask, at −56 °C, boron tribromide (0.35 mL, 3.60 mmol) was added by syringe. The reaction was monitored by TLC. After completion, the reaction was quenched by a small amount of methanol. After removal of the dichloromethane under reduced pressure, the residue was washed by water and brine, dried over anhydrous Na_2_SO_4_, and filtered. The filtrate was evaporated under vacuum to obtain compound **5a8** (yield 72%) as yellowish solid, m. p. 123.6–125.5 °C.

### 3.6. Antifungal Activity In Vitro

#### Determination of MIC_80_ and MFC Values

The antifungal activities in vitro of target compounds against five strains of fungi were determined via the BMD method with assays in 96-well plates as described in the CLSI guidelines (CLSI M27-A3 and M38-A2) [36,37,38]. FLC was selected as the positive control. To prepare the stock solutions of the tested compounds, they were dissolved in 100% DMSO with a concentration of 0.8 mg/mL. Before the assay, the solutions were diluted to a concentration of 8.0 µg/mL with RPMI medium 1640. The final concentrations of tested compounds were 64, 32, 16, 8, 4, 2, 1, 0.5, 0.25, and 0.125 µg/mL. After inoculation, the plates were incubated at 30 °C for 24 h. The absorbance of each well was scanned at a wavelength of 625 nm via an ELISA reader (DNM 9602, Perlong, Bingjing, China), and the inhibition rate was calculated.

$$Inhibitionrate\;(\%)=\frac{A_{p}-A_{D}}{A_{p}-A_{N}}\times 100\%$$

where AP is the absorbance of positive control wells, AD is the absorbance of drug wells, and AN is the absorbance of negative control wells. The MIC_80_ values were the concentration of tested compounds when the inhibition rate was at 80%. A curve was plotted with the logarithm of the concentration as the horizontal axis and the inhibition rate as the vertical axis, and the MIC_80_ was calculated.

$$MIC_{80}=10\log A+\log\frac{1}{N}\times \frac{a-80}{a-b}$$

Among them, *A* refers to the corresponding minimum concentration in the aforementioned gradient concentrations when the inhibition rate is just above 80%. a is the inhibition rate at concentration *A*, b is the inhibition rate just below a, and *N* represents the dilution factor.

## 4. Conclusions

Thirty novel trisubstituted 2-aminothiazole derivatives were efficiently synthesized by introducing different substituents (including benzamides, heterocyclic amides, and fatty amides) at the C2 position of the thiazole ring. Substituted aryl groups were also incorporated at the C4 and C5 positions with 2-aminothiazole as the parent nucleus. In the first round of experiments, the promising compound **4a8** was found to exhibit the best activity among the 29 candidates. Consequently, its demethylated derivative (compound **5a8**) was synthesized, and demonstrated activity comparable to fluconazole in anti-Candida albicans assays. Molecular docking calculations targeting four proteins were performed to gain insight into the anti-Candida albicans activity of **5a8**. The biological activity is primarily attributed to π-π stacking, hydrophobic interactions, and hydrogen bonding.

## Figures and Tables

**Table 2:** MIC_80_ of target compounds (**4a1–4c29**) against *Candida albicans* (μM).

Compound	FLC-Sensitive Strain	FLC-Resistant Strains			
	186382	4935	5122	5172	5272
**4a1**	167	>334	>334	>334	334
**4a8**	37	148	74	296	148
**4b19**	76	307	>307	>307	>307
**4b23**	73	>292	292	146	292
FLC	7	26	52	14	52

Note: (1) other compounds were also tested, but showed MIC_80_ values > 334 and were therefore not listed in the table. (2) FLC is fluconazole.

**Table 3:** MIC_80_ of most active compound, **5a8,** against *Candida albicans* (μM).

Compound	FLC-Sensitive Strain	FLC-Resistant Strains			
	186382	4935	5122	5172	4935
**5a8**	9	19	39	39	39
FLC	7	26	52	14	52

Note: FLC is fluconazole.

## Data Availability

No new data were created in this study.

## Supplementary Materials

The following supporting information can be downloaded at https://www.mdpi.com/article/10.3390/molecules30071643/s1, Figure S1: Mass spectrum of compound 4a1; Figure S2: ^1^H NMR And ^13^C NMR Spectrum of compound 4a1; Figure S3:Mass spectrum of compound 4a2; Figure S4:^1^H NMR And ^13^C NMR Spectrum of compound 4a2; Figure S5: Mass spectrum of compound 4a3; Figure S6:^1^H NMR And ^13^C NMR Spectrum of compound 4a3; Figure S7:Mass spectrum of compound 4a4; Figure S8:^1^H NMR And ^13^C NMR Spectrum of compound 4a4; Figure S9: Mass spectrum of compound 4a5; Figure S10: ^1^H NMR And ^13^C NMR Spectrum of compound 4a5; Figure S11: Mass spectrum of compound 4a6; Figure S12: ^1^H NMR And ^13^C NMR Spectrum of compound 4a6; Figure S13: Mass spectrum of compound 4a7; Figure S14: ^1^H NMR And ^13^C NMR Spectrum of compound 4a7; Figure S15: Mass spectrum of compound 4a8; Figure S16:^1^H NMR And ^13^C NMR Spectrum of compound 4a8; Figure S17: Mass spectrum of compound 4a9; Figure S18: ^1^H NMR And ^13^C NMR Spectrum of compound 4a9; Figure S19: Mass spectrum of compound 4a10; Figure S20: ^1^H NMR And ^13^C NMR Spectrum of compound 4a10; Figure S21: Mass spectrum of compound 4a11; Figure S22: ^1^H NMR And ^13^C NMR Spectrum of compound 4a11; Figure S23: Mass spectrum of compound 4a12; Figure S24: ^1^H NMR And ^13^C NMR Spectrum of compound 4a12; Figure S25: Mass spectrum of compound 4a13; Figure S26: ^1^H NMR And ^13^C NMR Spectrum of compound 4a13; Figure S27: Mass spectrum of compound 4a14; Figure S28: ^1^H NMR And ^13^C NMR Spectrum of compound 4a14; Figure S29: Mass spectrum of compound 4a15; Figure S30: ^1^H NMR And ^13^C NMR Spectrum of compound 4a15; Figure S31: Mass spectrum of compound 4a16; Figure S32: ^1^H NMR And ^13^C NMR Spectrum of compound 4a16; Figure S33: Mass spectrum of compound 4a17; Figure S34: ^1^H NMR And ^13^C NMR Spectrum of compound 4a17; Figure S35: Mass spectrum of compound 4a18; Figure S36: ^1^H NMR And ^13^C NMR Spectrum of compound 4a18; Figure S37: Mass spectrum of compound 4b19; Figure S38: ^1^H NMR And ^13^C NMR Spectrum of compound 4b19; Figure S39: Mass spectrum of compound 4b20; Figure S40: ^1^H NMR And ^13^C NMR Spectrum of compound 4b20; Figure S41: Mass spectrum of compound 4b21; Figure S42: ^1^H NMR And ^13^C NMR Spectrum of compound 4b21; Figure S43: Mass spectrum of compound 4b22; Figure S44: ^1^H NMR And ^13^C NMR Spectrum of compound 4b22; Figure S45: Mass spectrum of compound 4b23; Figure S46:^1^H NMR And ^13^C NMR Spectrum of compound 4b2; Figure S47. Mass spectrum of compound 4c24; Figure S48: ^1^H NMR And ^13^C NMR Spectrum of compound 4c24; Figure S49:Mass spectrum of compound 4c25; Figure S50: ^1^H NMR And ^13^C NMR Spectrum of compound 4c25; Figure S51:Mass spectrum of compound 4c26; Figure S52: ^1^H NMR And ^13^C NMR Spectrum of compound 4c26; Figure S53:Mass spectrum of compound 4c27; Figure S54:^1^H NMR And ^13^C NMR Spectrum of compound 4c27; Figure S55: Mass spectrum of compound 4c28; Figure S56:^1^H NMR And ^13^C NMR Spectrum of compound 4c28; Figure S57:Mass spectrum of compound 4c29; Figure S58:^1^H NMR And ^13^C NMR Spectrum of compound 4c29; Figure S59: Mass spectrum of compound 5a8; Figure S60: ^1^H NMR And ^13^C NMR Spectrum of compound 5a8; Figure S61: ^1^H NMR Spectrum of compound 3a; Figure S62: ^13^C NMR Spectrum of compound 3a; Figure S63: ^1^H NMR Spectrum of compound 3b; Figure S64: ^13^C NMR Spectrum of compound 3b; Figure S65: ^1^H NMR Spectrum of compound 3c; Figure S66: ^13^C NMR Spectrum of compound 3c.All NMR and HRMS spectra are included in the Supplementary Information (SI).
