# Supplementary material for: Synthesis of 2-Amino-4, 5-Diarylthiazole Derivatives and Evaluation of Their Anti-Candida Albicans Activity
Source: Molecules. 2025 Apr 7;30(7):1643. doi: 10.3390/molecules30071643 (PMC11990618; doi:10.3390/molecules30071643)
Supplement: Supplementary file 1 [file molecules-30-01643-s001.zip › molecules-3539207-supplementary.pdf]

Supporting information

Synthesis of 2-Amino-4, 5-diarylthiazole derivatives and evaluation of their anti-Candida albicans activity

Dongmei Gao[1], Lele Shi[2], Yuhang Huang[2], Yingmei Lv[2], Xuan Yang[2], Zhenting Du[*2]

[1] Yangling Vocational & Technical College, Yangling, 712100, Shaanxi, China

[2]School of Chemistry and Pharmacy, Northwest A & F University, Yangling, 712100, Shaanxi, China

_N_-[4-(3, 4-Dimethoxyphenyl)-5-phenyl-1, 3-thiazol-2-yl]butanamide (4a1)

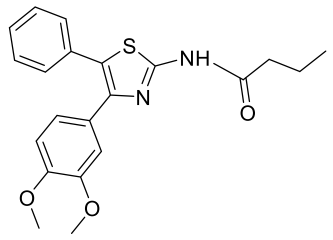

4a1, yield, 65%, white solid, m.p. 171.2-172.7°C. **^{1}H NMR** (500 MHz, $CDCl_3$) δ 11.58 (s, 1H), 7.41 – 7.36 (m, 2H), 7.34 (d, J = 6.7 Hz, 2H), 7.34 – 7.26 (m, 1H), 7.16 (dd, J = 8.3, 2.0 Hz, 1H), 6.92 (d, J = 2.0 Hz, 1H), 6.82 (d, J = 8.4 Hz, 1H), 3.87 (s, 3H), 3.58 (s, 3H), 1.83 – 1.71 (t, 2H), 1.45 (h, J = 7.4 Hz, 2H), 0.70 (t, J = 7.4 Hz, 3H).**^{13}C NMR** (125 MHz, $CDCl_3$) δ 171.8, 157.7, 148.9, 148.6, 143.5, 132.2, 129.7, 128.8, 127.9 127.2, 125.6, 121.5, 112.0, 111.0, 55.8, 55.6, 35.4, 31.6, 28.9, 28.9, 24.7, 22.6, 14.1. HRMS, cald. ($C_{21}H_{22}N_2O_3S$+H) =383.1431, Found, 383.1433.

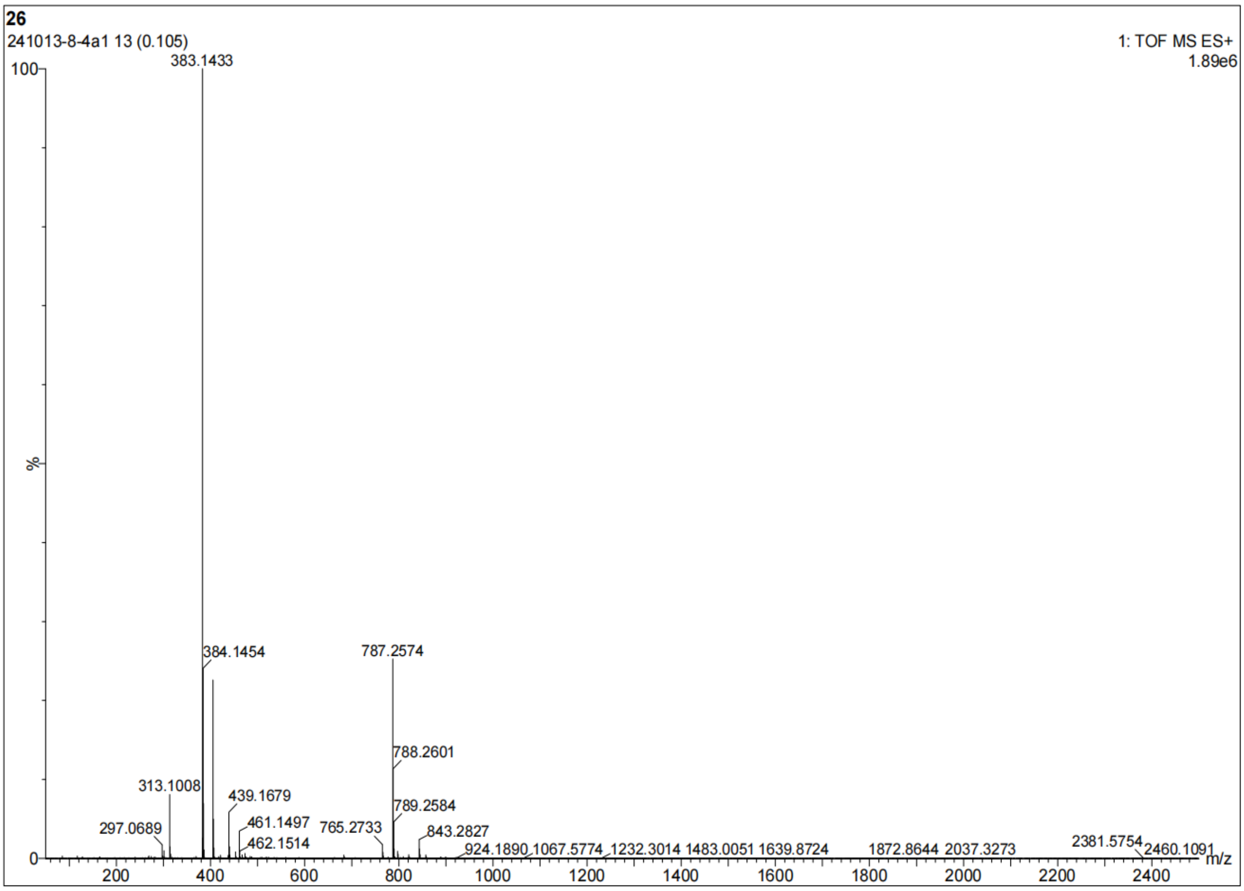

Fig.S1. Mass spectrum of compound 4a1

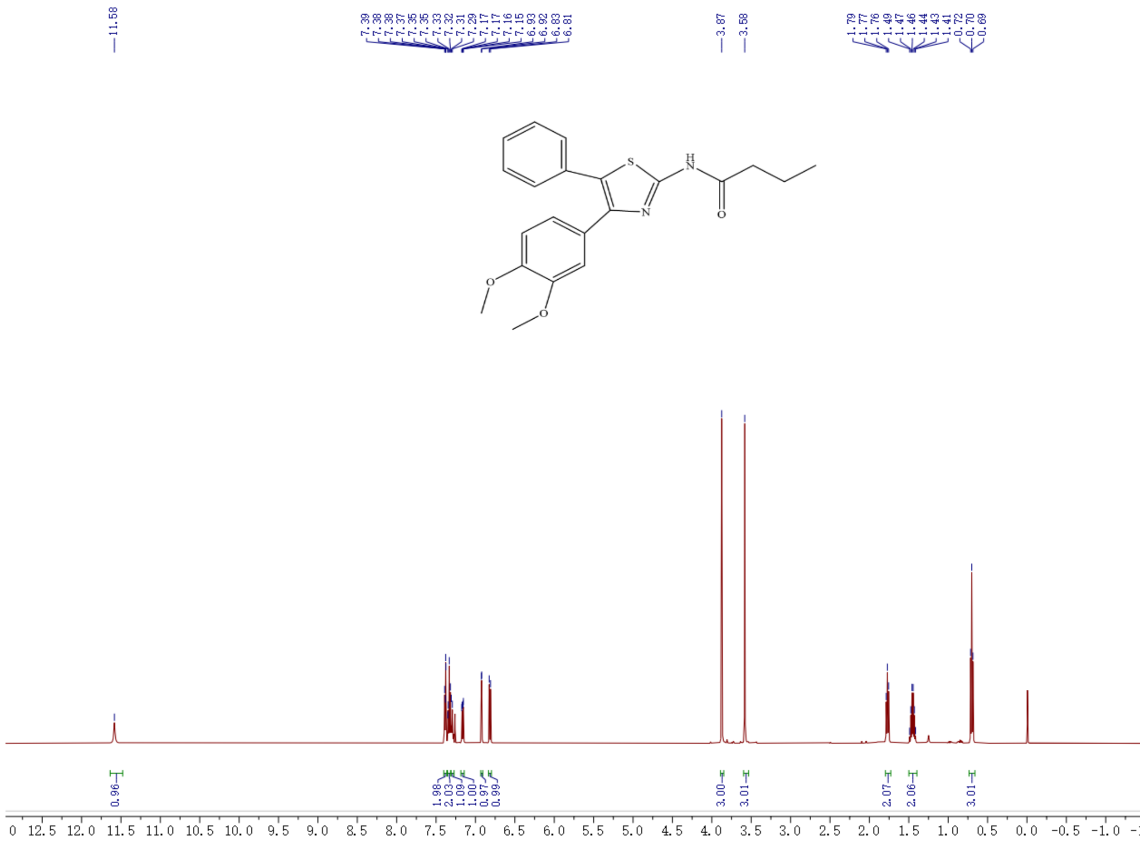

Fig.S2. ^{1}H NMR And ^{13}C NMR Spectrum of compound 4a1

N-[4-(3, 4-Dimethoxyphenyl)-5-phenyl-1, 3-thiazol-2-yl]hexanamide (4a2)

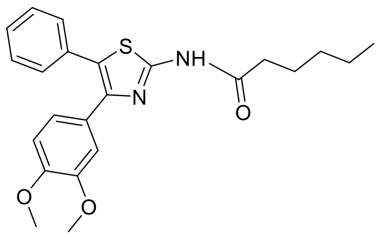

4a2, yield, 63%, white solid, m.p. 208.1-209.6°C. **^{1}H NMR** (500 MHz, $CDCl_3$) δ 11.60 (s, 1H), 7.44 (d, J = 7.2 Hz, 2H), 7.37 (dt, J = 12.7, 7.1 Hz, 2H), 7.22 (d, J = 8.2 Hz, 1H), 6.98 (s, 1H), 6.86 (d, J = 8.4 Hz, 1H), 3.92 (s, 3H), 3.62 (s, 3H), 1.85 (t, J = 7.7 Hz, 2H), 1.47 (p, J = 7.8 Hz, 2H), 1.21 (h, J = 7.1, 5.9 Hz, 2H), 1.06 (p, J = 8.6, 8.1 Hz, 2H), 0.85 (t, J = 7.3 Hz, 3H).**^{13}C NMR** (125 MHz, $CDCl_3$) δ 171.8, 157.7, 148.9, 148.7, 143.6, 132.3, 129.7, 128.8, 127.8, 127.3, 125.6, 121.5, 112.2, 111.2, , 55.9, 55.6, 35.4, 31.2, 24.4, 22.3, 13.8. HRMS, cald. ($C_{23}H_{26}N_2O_3S$+H) =411.1742, Found, 411.1744.

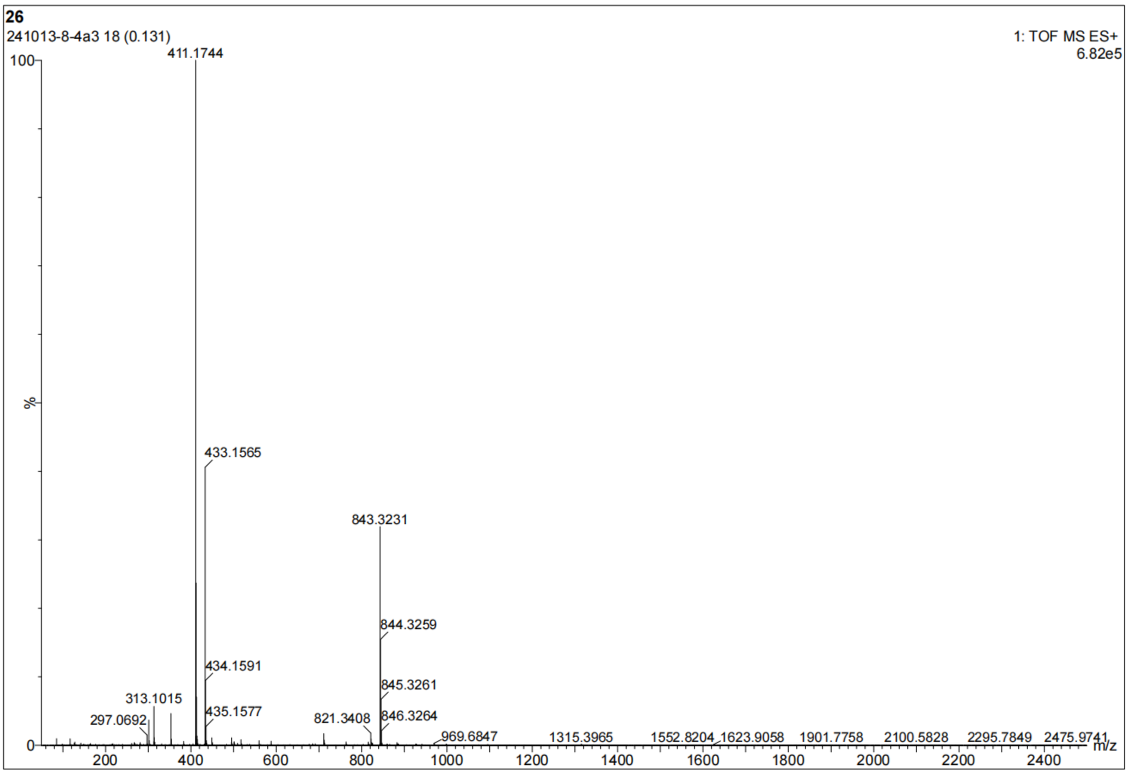

Fig.S3. Mass spectrum of compound 4a2

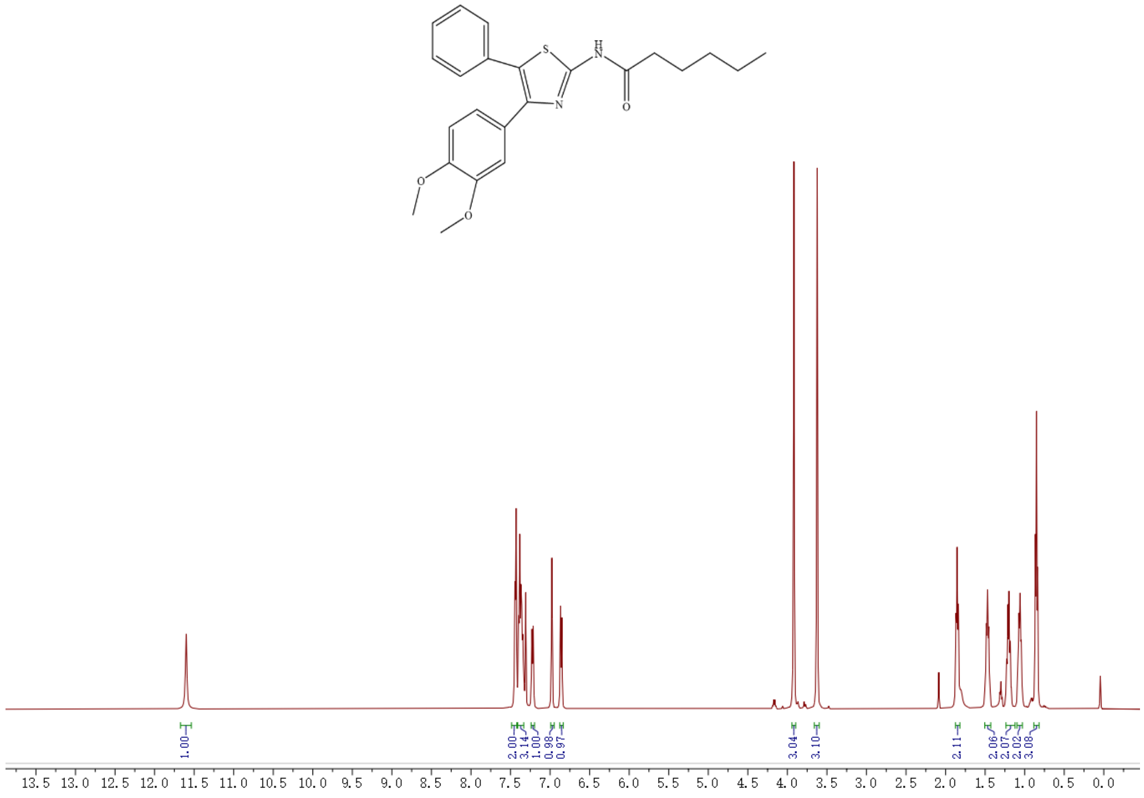

S
H
N
O
N
O
O
1.00
2.00
3.14
1.00
0.98
0.97
3.04
3.10
2.11
2.06
2.07
2.02
3.08
13.5 13.0 12.5 12.0 11.5 11.0 10.5 10.0 9.5 9.0 8.5 8.0 7.5 7.0 6.5 6.0 5.5 5.0 4.5 4.0 3.5 3.0 2.5 2.0 1.5 1.0 0.5 0.0

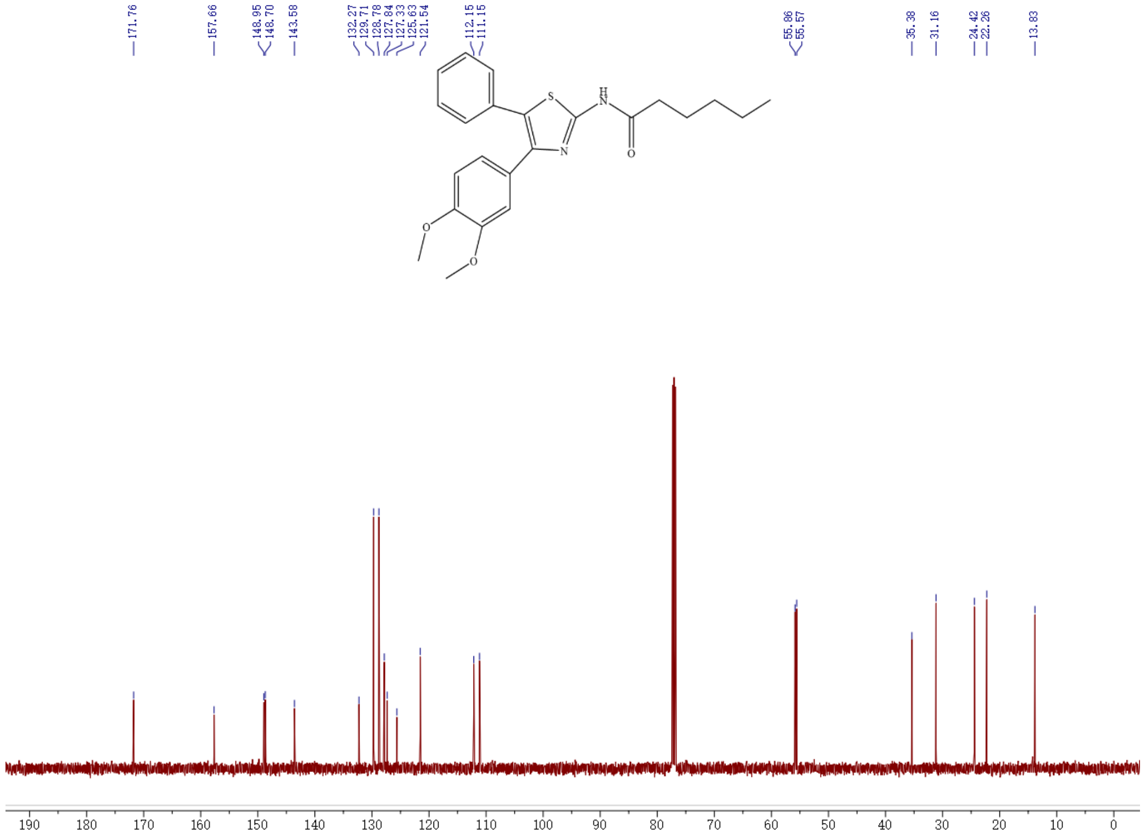

Fig.S4. ^{1}H NMR And ^{13}C NMR Spectrum of compound 4a2

N-[4-(3, 4-Dimethoxyphenyl)-5-phenyl-1, 3-thiazol-2-yl]octanamide (4a3)

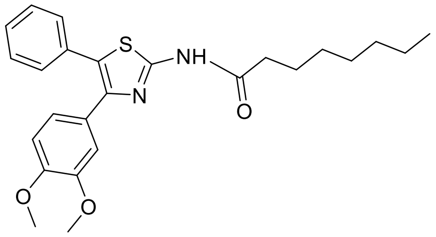

4a3, yield, 64%, white solid, m.p. 136.1-137.0°C. **^{1}H NMR** (500 MHz, $CDCl_3$) δ 11.66 (s, 1H), 7.41 – 7.36 (m, 2H), 7.34 (d, *J* = 6.7 Hz, 2H), 7.33 – 7.27 (m, 1H), 7.16 (dd, *J* = 8.4, 2.0 Hz, 1H), 6.91 (d, *J* = 2.0 Hz, 1H), 6.80 (d, *J* = 8.4 Hz, 1H), 3.87 (s, 3H), 3.57 (s, 3H), 1.78 (t, *J* = 7.7 Hz, 2H), 1.40 (p, *J* = 7.7 Hz, 2H), 1.23 (q, *J* = 7.4 Hz, 2H), 1.14 (p, *J* = 4.1, 3.6 Hz, 4H), 1.00 (p, *J* = 7.4, 7.0 Hz, 2H), 0.84 (t, *J* = 7.2 Hz, 3H).**^{13}C NMR** (125 MHz, $CDCl_3$) δ 171.8, 157.7, 148.9, 148.6, 143.5, 132.2, 129.7, 128.8, 127.2, 125.6, 121.5, 112.0, 111.0, 55.8, 55.6, 35.4, 31.6, 28.9, 28.9, 24.8, 22.6, 14.1. HRMS, cald. ($C_{25}H_{30}N_2O_3S$+H) = 439.2055, Found, 439.2051.

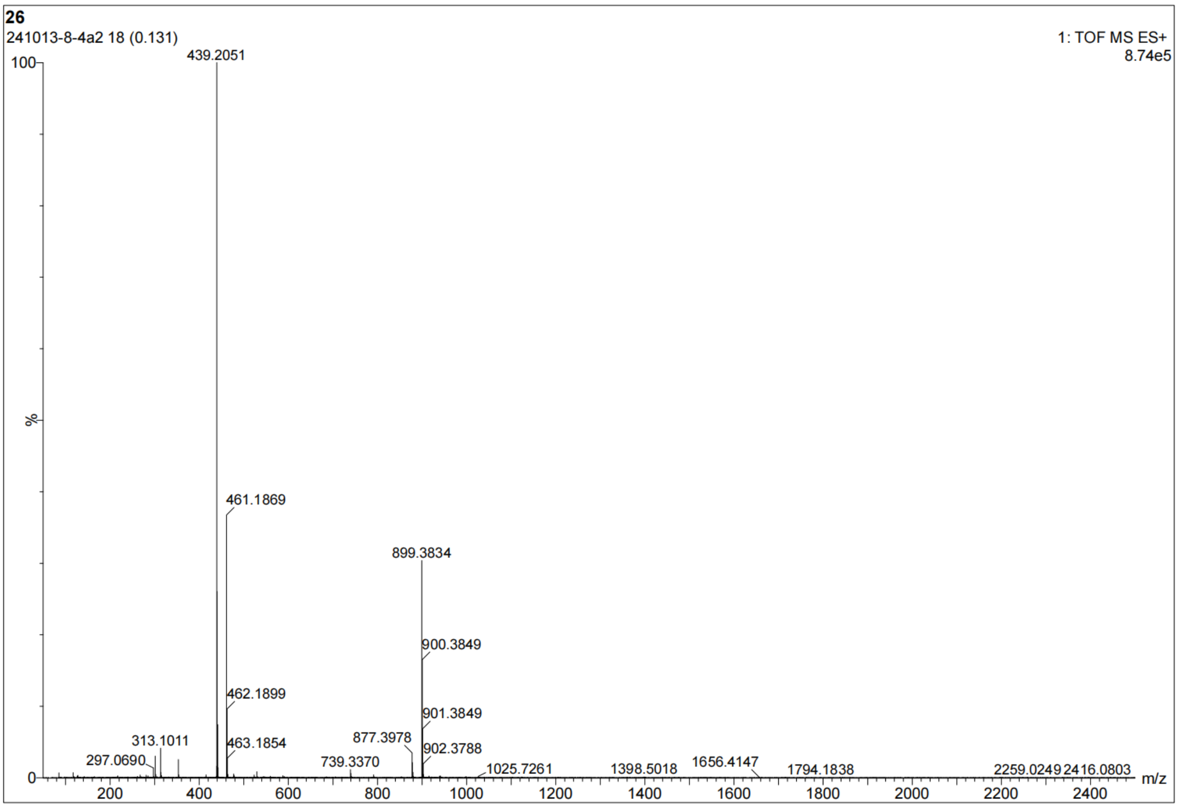

Fig.S5. Mass spectrum of compound 4a3

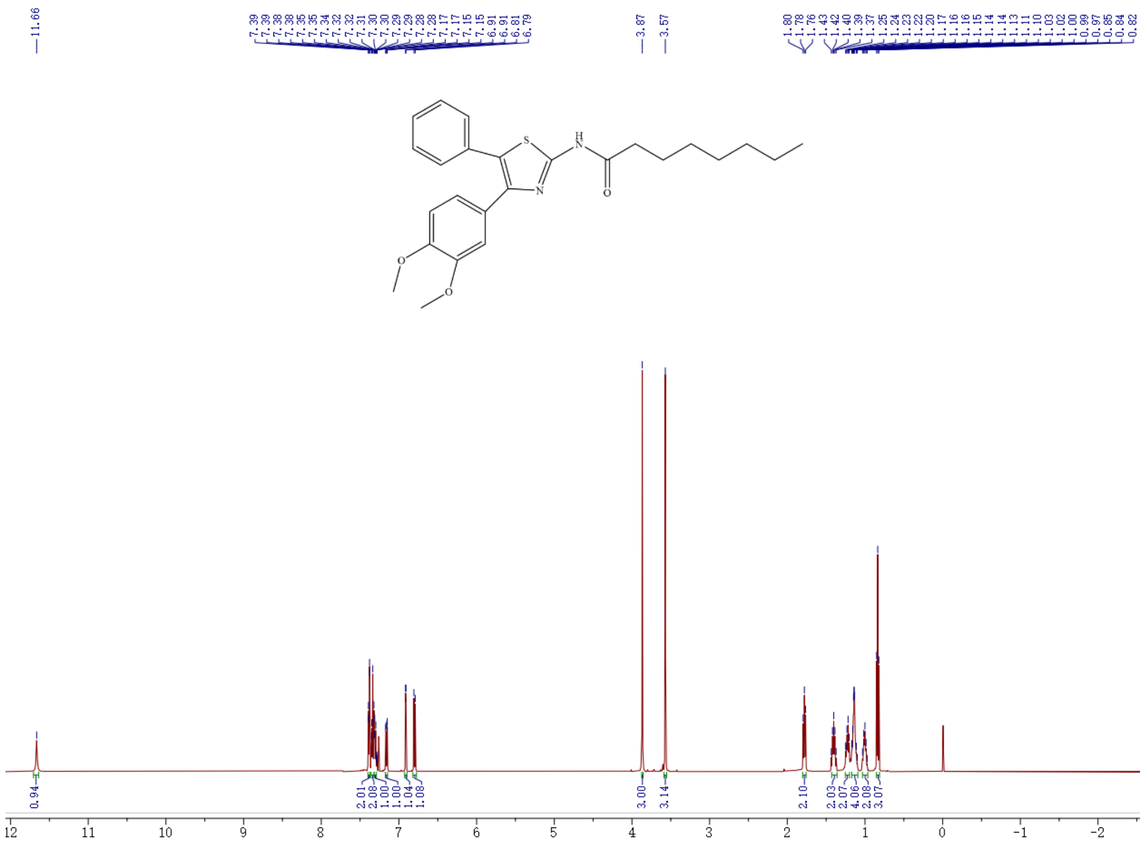
11.66
7.39 7.39 7.38 7.38 7.35 7.35 7.34 7.32 7.31 7.30 7.30 7.29 7.29 7.28 7.28 7.17 7.17 7.15 7.15 6.91 6.91 6.81 6.79
3.87
3.57
1.80 1.78 1.76 1.43 1.42 1.40 1.39 1.37 1.25 1.24 1.23 1.22 1.20 1.17 1.16 1.15 1.14 1.13 1.11 1.10 1.03 1.02 1.00 0.99 0.97 0.85 0.84 0.82
S
N
H
O
0.94
2.01 2.08 1.00 1.00 1.04 1.08
3.00
3.14
2.10
2.03 2.07 4.06 2.08 3.07
12 11 10 9 8 7 6 5 4 3 2 1 0 -1 -2

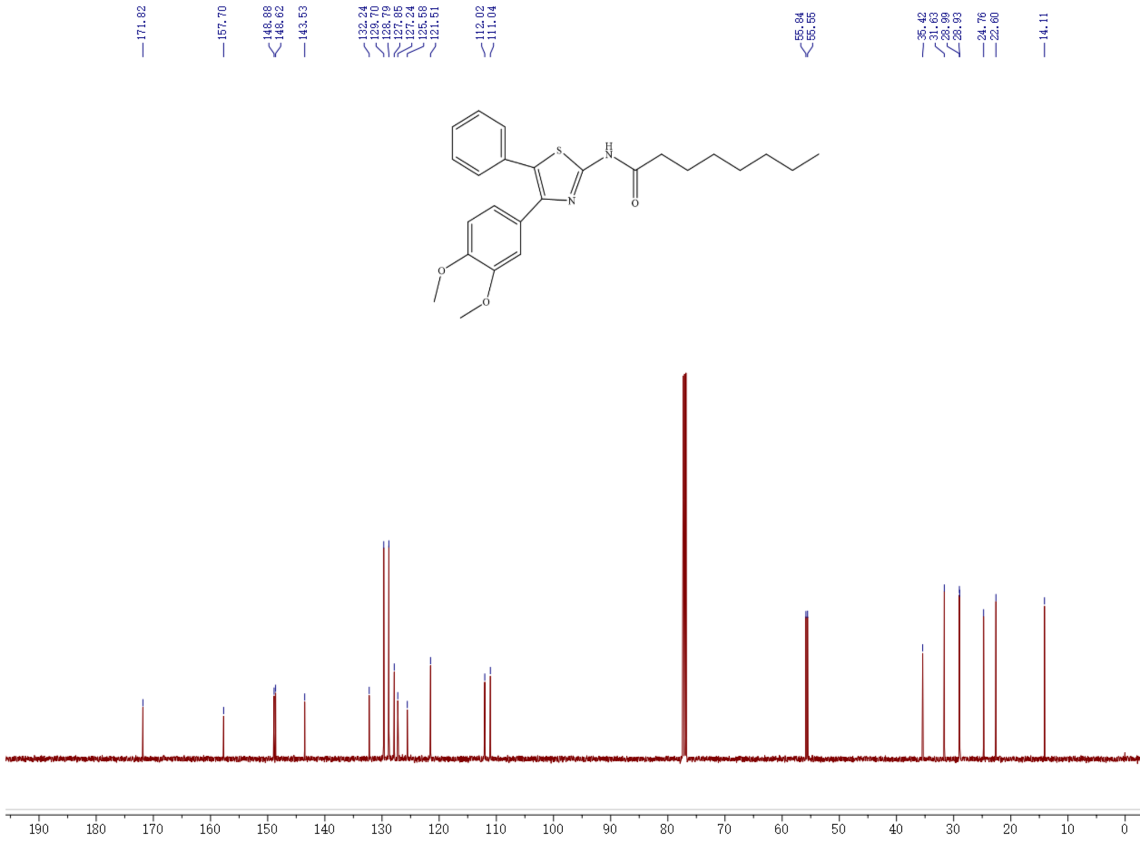
171.82
157.70
148.88 148.62
143.53
132.24 129.70 128.79 127.85 127.24 125.58 121.51
112.02 111.04
55.84 55.55
35.42 31.63 28.99 28.93 24.76 22.60
14.11
S
N
H
O
190 180 170 160 150 140 130 120 110 100 90 80 70 60 50 40 30 20 10 0

Fig.S6. ^{1}H NMR And ^{13}C NMR Spectrum of compound 4a3

N-[4-(3, 4-Dimethoxyphenyl)-5-phenyl-1, 3-thiazol-2-yl]benzamide (4a4)

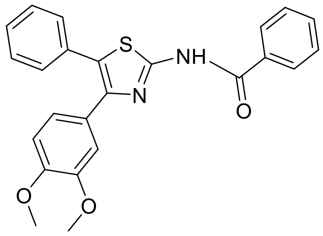

4a4, yield, 68%, white solid, m.p. 134.0-135.5°C. **^{1}H NMR** (500 MHz, $CDCl_3$) δ 11.46 (s, 1H), 7.82 (d, J = 7.4 Hz, 2H), 7.50 – 7.43 (m, 3H), 7.38 – 7.34 (m, 5H), 7.00 (d, J = 9.9 Hz, 1H), 6.84 (s, 1H), 6.64 (d, J = 8.4 Hz, 1H), 3.84 (s, 3H), 3.60 (s, 3H).**^{13}C NMR** (125 MHz, $CDCl_3$) δ 165.2, 157.2, 148.5, 148.2, 144.4, 132.5, 132.3, 131.8, 129.7, 128.8, 128.6, 127.8, 127.5, 127.1, 126.1, 121.4, 111.9, 110.9, 55.8, 55.4. HRMS, cald. ($C_{24}H_{20}N_2O_3S$+H) = 417.1273, Found,417.1276.

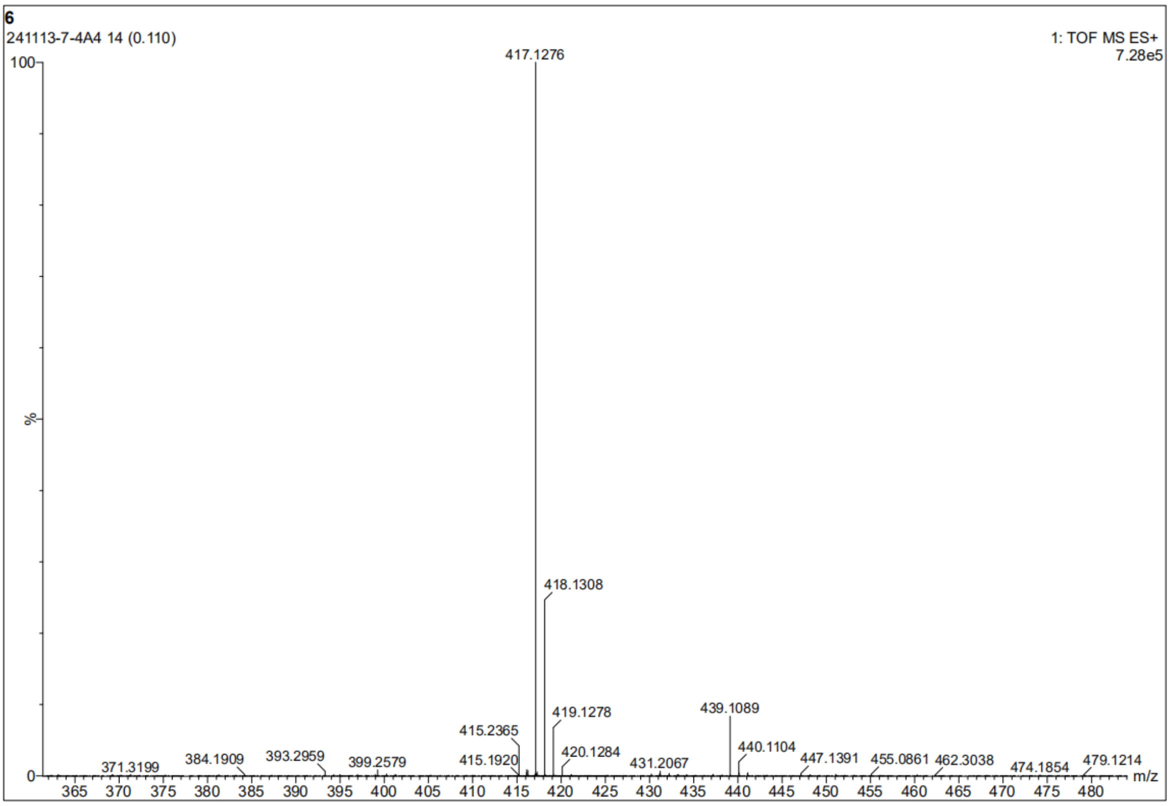

Fig.S7. Mass spectrum of compound 4a4

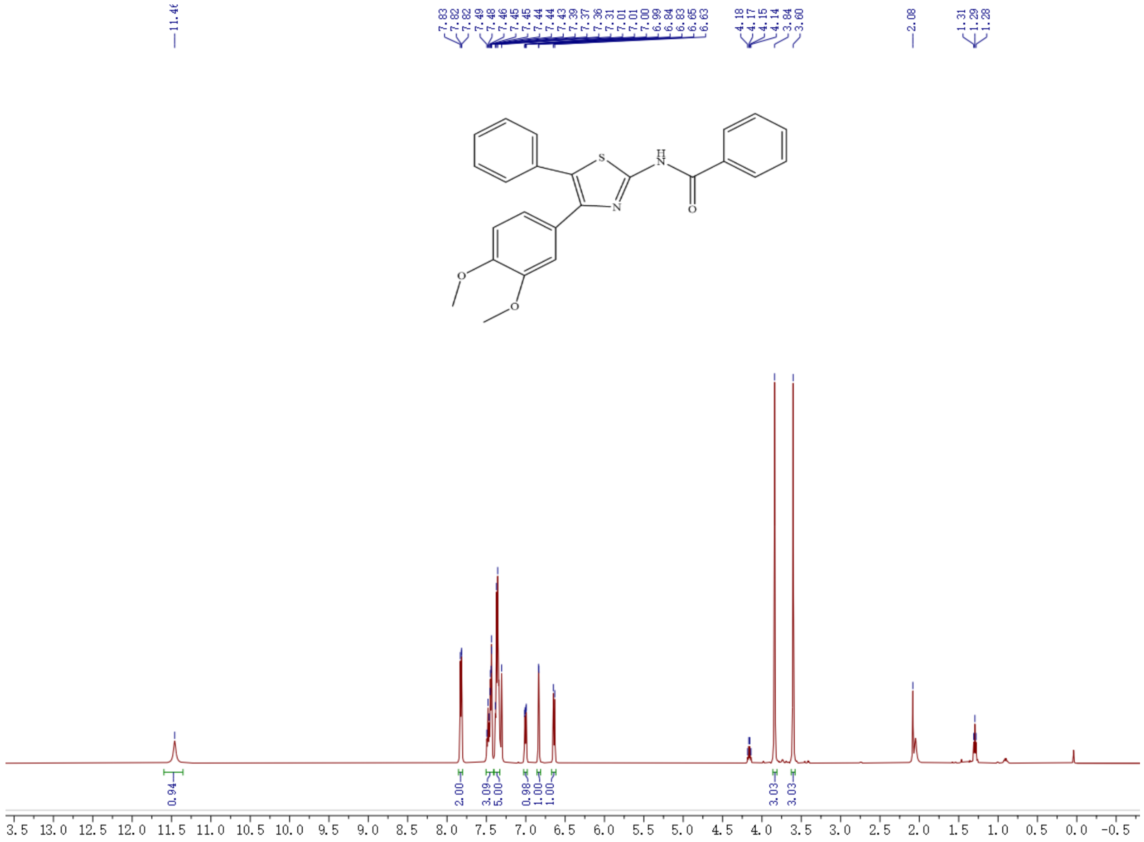
11.46
7.83
7.82
7.82
7.49
7.48
7.46
7.45
7.45
7.45
7.44
7.44
7.43
7.39
7.37
7.36
7.31
7.01
7.00
6.99
6.84
6.83
6.65
6.63
4.18
4.17
4.15
4.14
3.84
3.60
2.08
1.31
1.29
1.28
S
H
N
N
O
O
O
0.94
2.00
3.09
5.00
0.98
1.00
1.00
3.03
3.03
3.5 13.0 12.5 12.0 11.5 11.0 10.5 10.0 9.5 9.0 8.5 8.0 7.5 7.0 6.5 6.0 5.5 5.0 4.5 4.0 3.5 3.0 2.5 2.0 1.5 1.0 0.5 0.0 -0.5

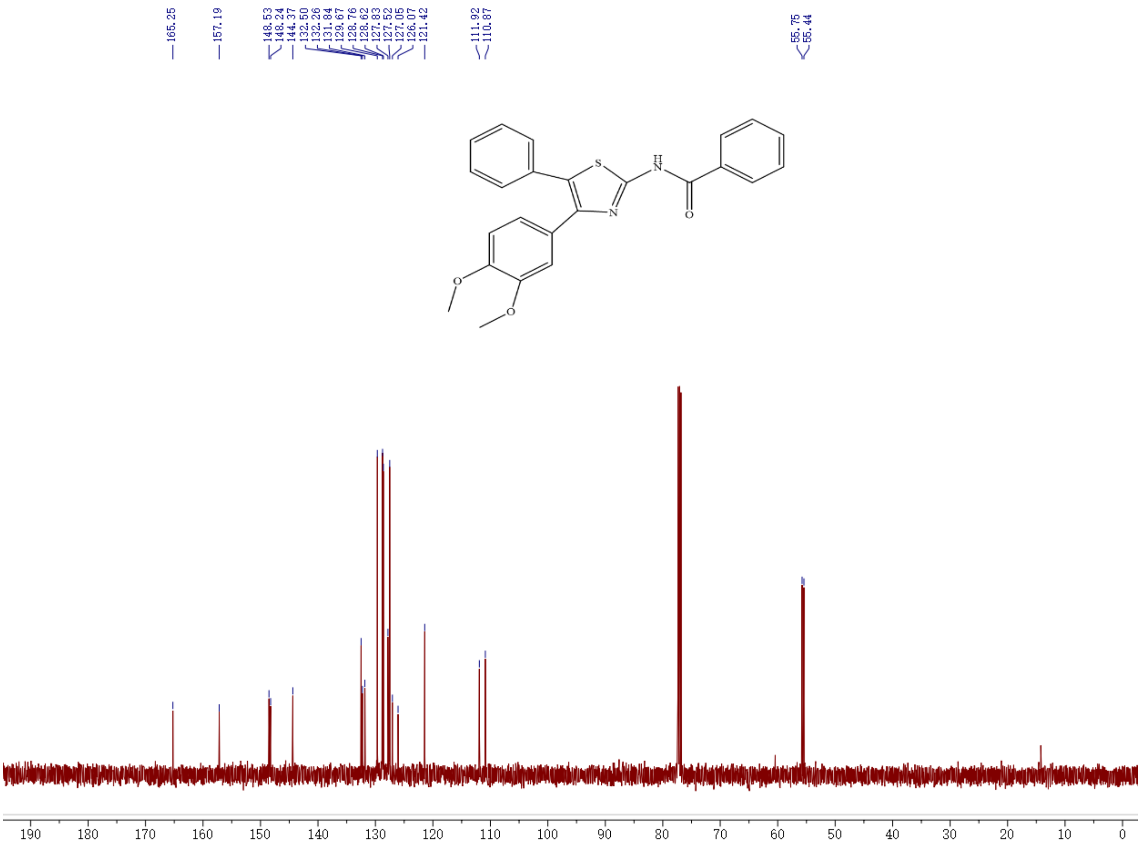

Fig.S8. ^{1}H NMR And ^{13}C NMR Spectrum of compound 4a4

2-{[4-(3, 4-dimethoxyphenyl)-5-phenyl-1, 3-thiazol-2-yl]amino}-1-phenylethan-1-one (4a5)

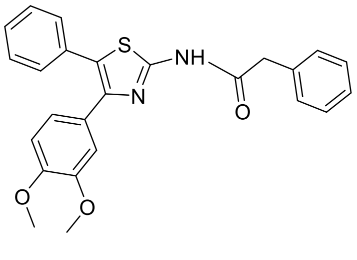

4a5, yield, 66%, white solid, m.p. 177.1-177.8°C. **^{1}H NMR** (500 MHz, $CDCl_3$) δ 10.70 (s, 1H), 7.42 (d, J = 8.2 Hz, 2H), 7.38 – 7.29 (m, 7H), 7.13 (d, J = 7.7 Hz, 2H), 6.98 (s, 1H), 6.83 (d, J = 8.4 Hz, 1H), 3.90 (s, 3H), 3.63 (s, 3H), 3.45 (s, 2H).**^{13}C NMR** (125 MHz, $CDCl_3$) δ 169.2, 156.7, 148.9, 148.7, 143.9, 133.2, 132.2, 129.7, 129.3, 128.9, 128.8, 127.9, 127.6, 127.4, 126.1, 121.5, 112.1, 111.2, 55.9, 55.6, 42.7. HRMS, cald. ($C_{25}H_{22}N_2O_3S$+H) = 431.1429, Found,431.1432.

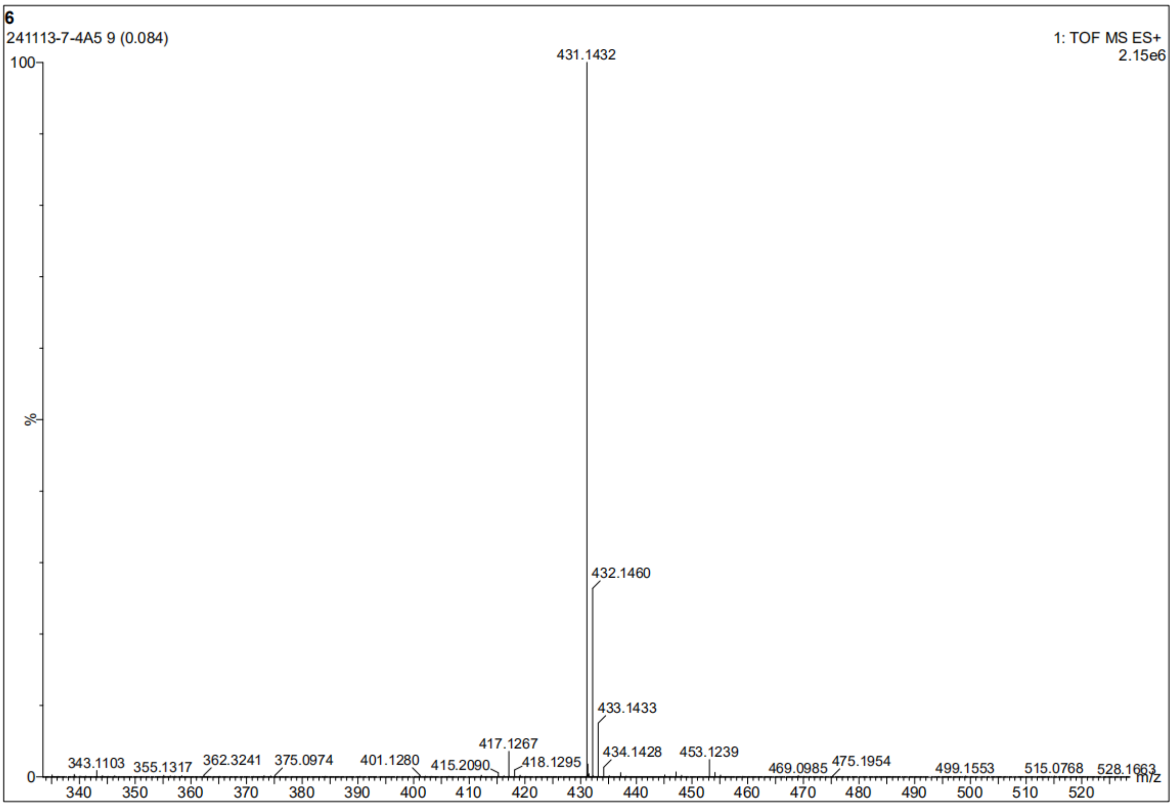

Fig.S9. Mass spectrum of compound 4a5

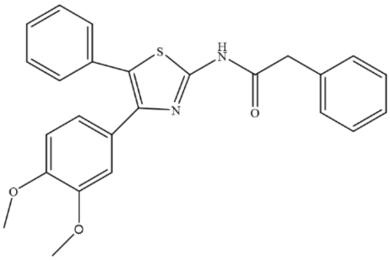

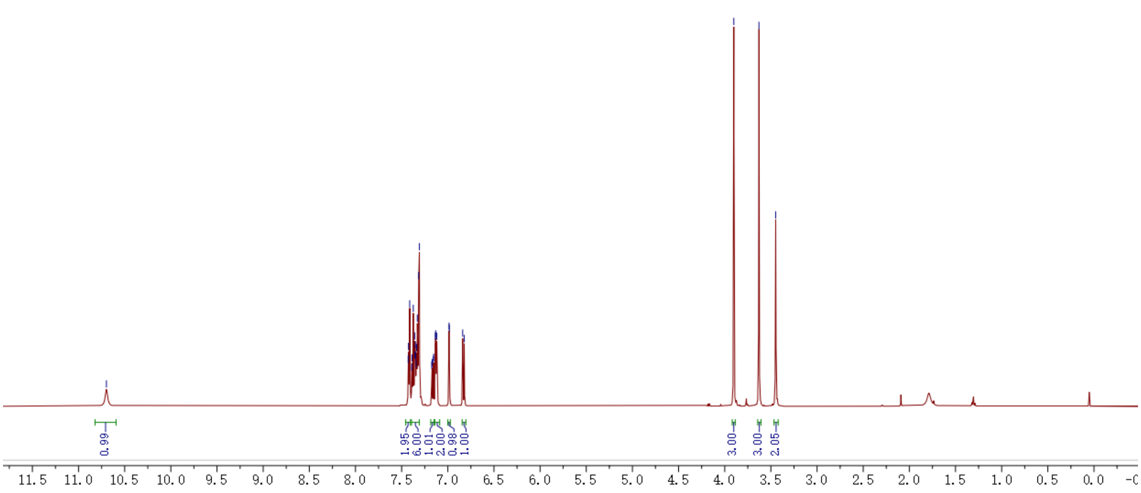

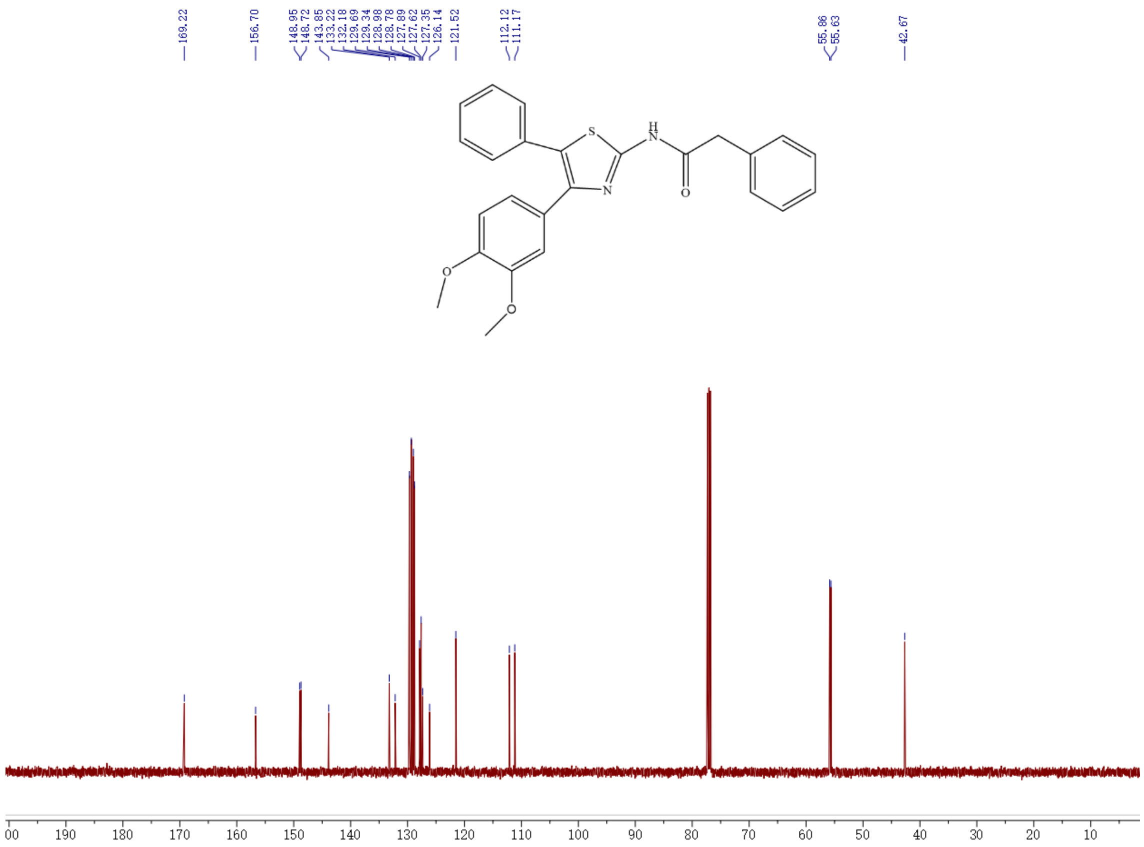

Fig.S10. ^{1}H NMR And ^{13}C NMR Spectrum of compound 4a5

_N_-[4-(3, 4-Dimethoxyphenyl)-5-phenyl-1, 3-thiazol-2-yl]-2-methylbenzamide (4a6)

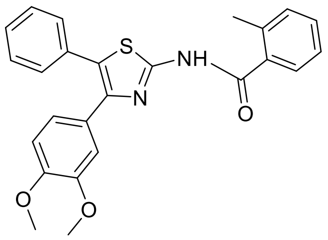

4a6, yield, 68%, white solid, m.p. 111.6-114.5°C. **^{1}H NMR** (500 MHz, $CDCl_3$) δ 11.35 (s, 1H), 7.44 – 7.41 (m, 3H), 7.40 – 7.33 (m, 3H), 7.32 – 7.27 (m, 1H), 7.18 (d, J = 7.7 Hz, 1H), 7.12 (t, J = 7.5 Hz, 1H), 7.00 (dd, J = 8.4, 2.0 Hz, 1H), 6.74 (d, J = 2.0 Hz, 1H), 6.71 (d, J = 8.3 Hz, 1H), 3.90 (s, 3H), 3.60 (s, 3H), 2.49 (s, 3H).**^{13}C NMR** (125 MHz, $CDCl_3$) δ 167.2, 156.9, 148.5, 148.1, 144.1, 138.0, 132.7, 132.3, 131.5, 131.2, 129.7, 128.7, 127.8, 127.3, 126.9, 125.7, 125.6, 121.2, 111.8, 110.7, 55.8, 55.4, 20.3. HRMS, cald. ($C_{25}H_{22}N_2O_3S$+H) = 431.1429, Found,431.1433.

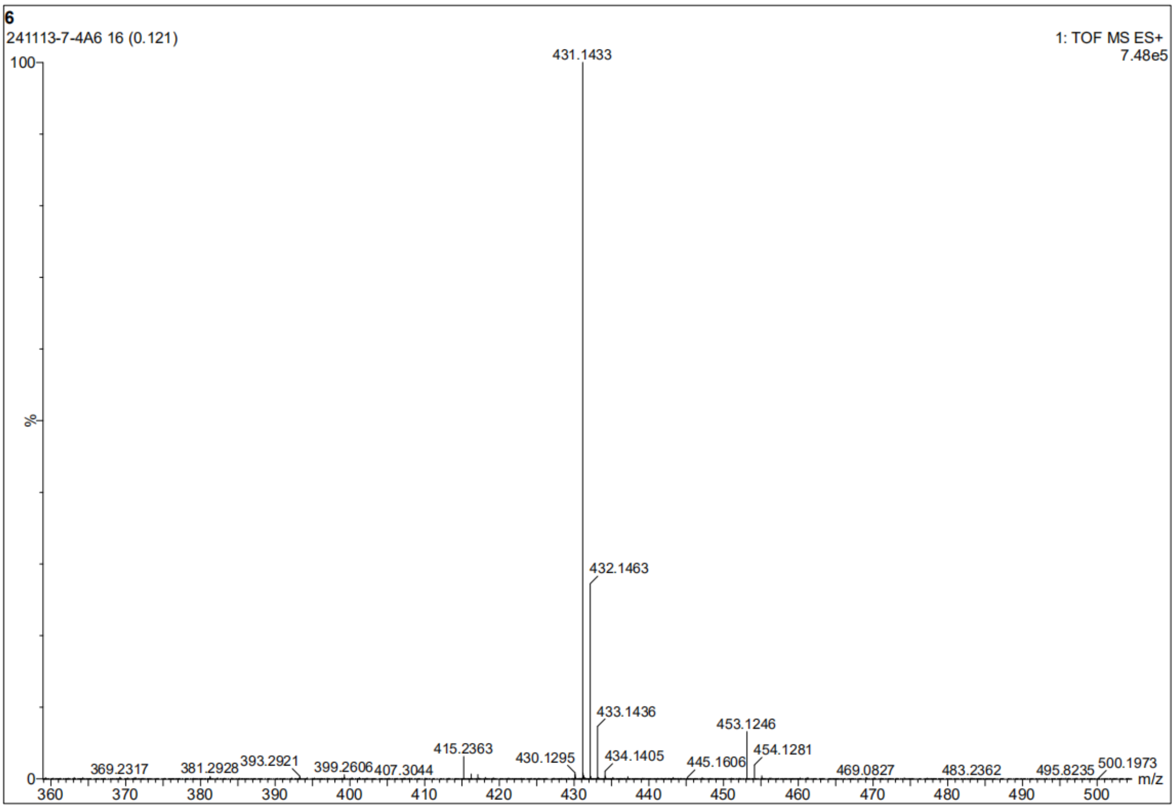

Fig.S11. Mass spectrum of compound 4a6

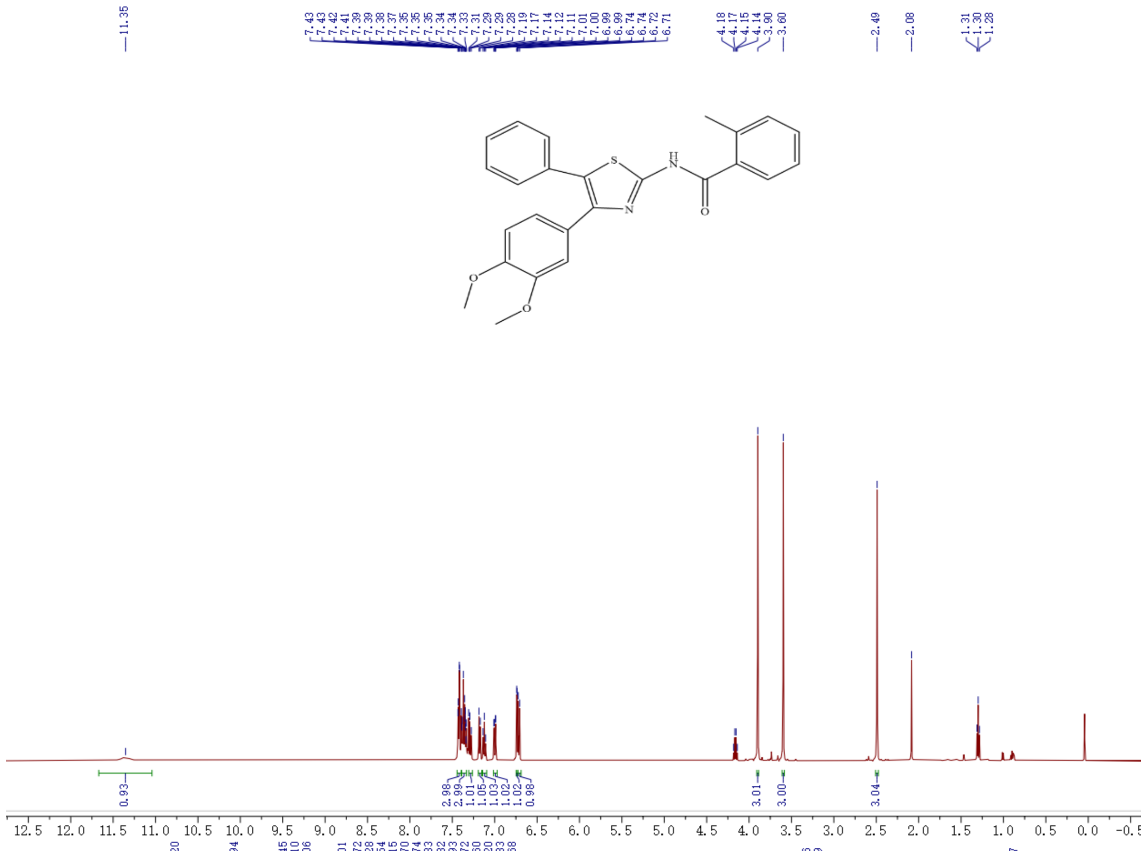

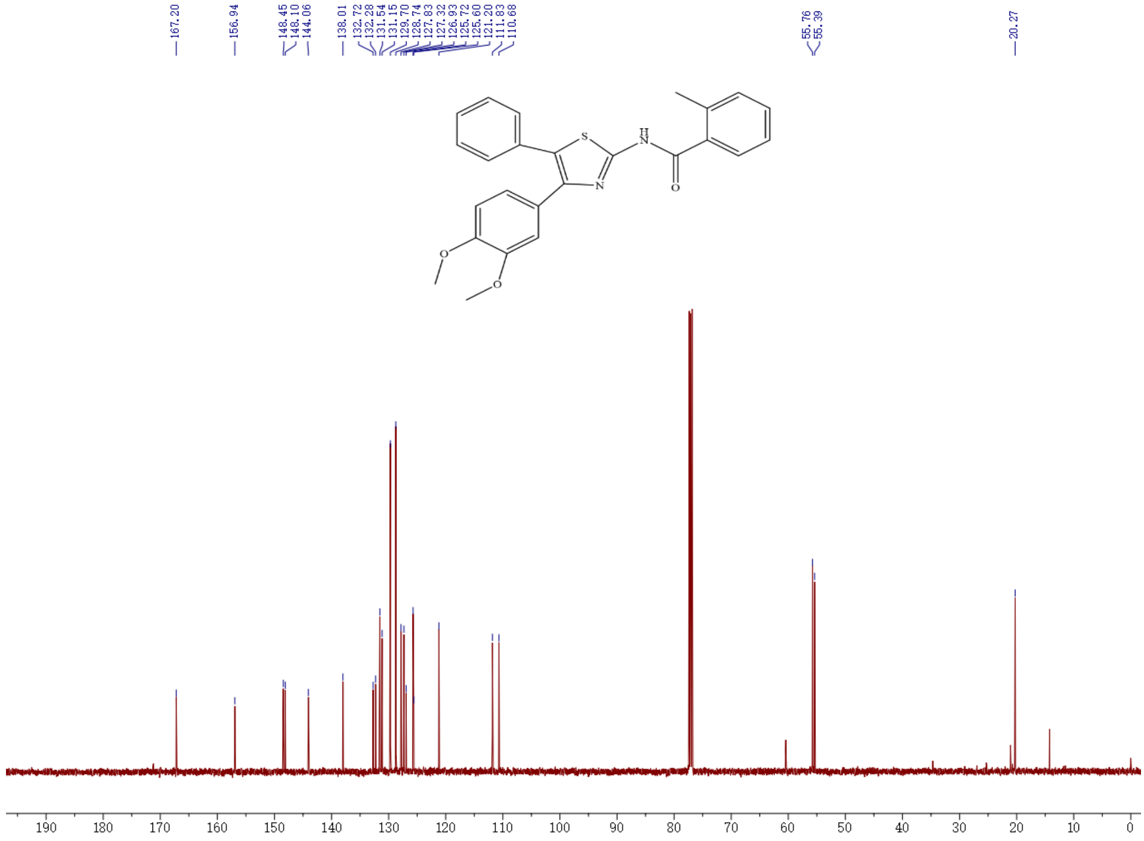

Fig.S12. ^{1}H NMR And ^{13}C NMR Spectrum of compound 4a6

N-[4-(3, 4-Dimethoxyphenyl)-5-phenyl-1, 3-thiazol-2-yl]-3-methylbenzamide (4a7)

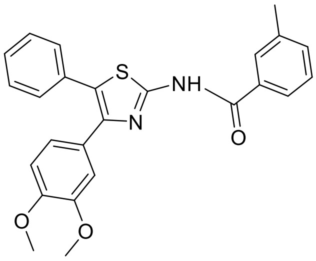

4a7, yield, 65%, white solid, m.p. 137.4-138.2℃. **^{1}H NMR** (500 MHz, $CDCl_3$) δ 11.11 (s, 1H), 7.64 (d, J = 11.8 Hz, 2H), 7.46 (d, J = 6.6 Hz, 2H), 7.42 – 7.34 (m, 3H), 7.33 – 7.27 (m, 2H), 7.04 (dd, J = 8.3, 2.0 Hz, 1H), 6.87 (d, J = 2.0 Hz, 1H), 6.68 (d, J = 8.4 Hz, 1H), 3.86 (s, 3H), 3.62 (s, 3H), 2.38 (s, 3H).**^{13}C NMR** (125 MHz, $CDCl_3$) δ 165.3, 156.9, 148.5, 148.2, 144.3, 138.6, 133.4, 132.3, 131.8, 129.7, 128.8, 128.6, 128.2, 127.9, 127.1, 126.0, 124.6, 121.3, 111.8, 110.8, 55.8, 55.4, 21.4. HRMS, cald. ($C_{25}H_{22}N_2O_3S$+H) = 431.1429, Found,431.1433.

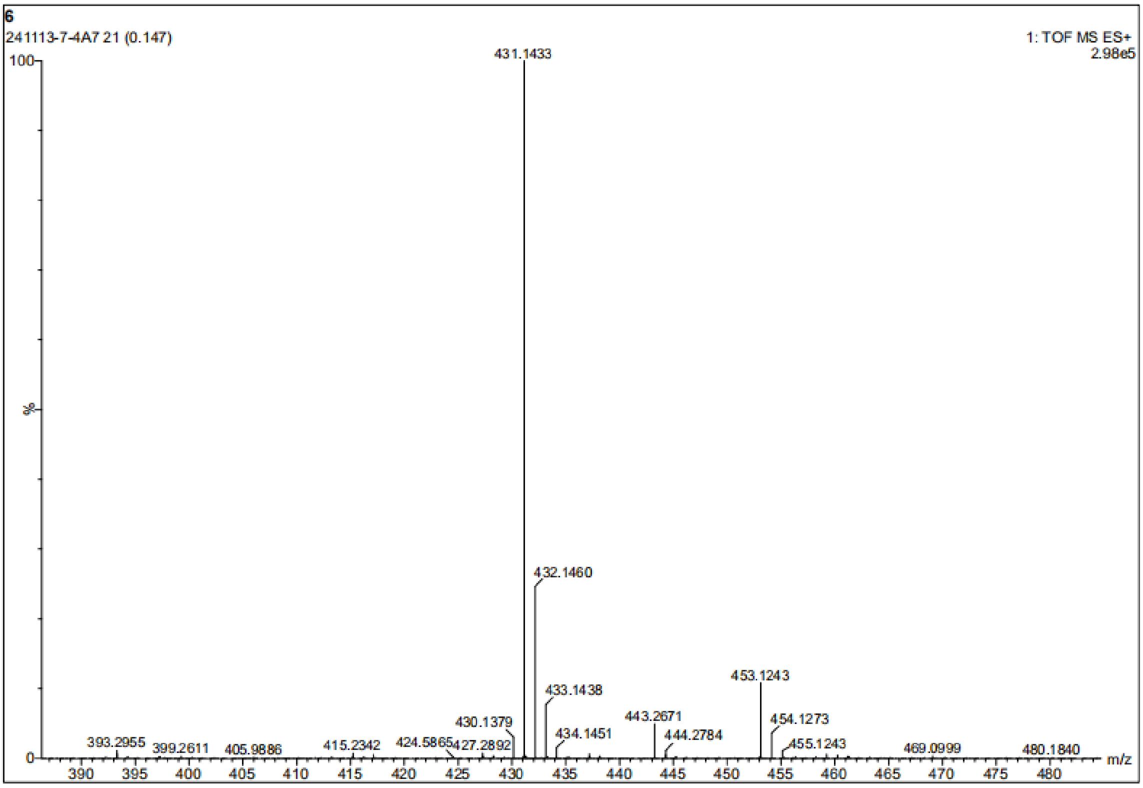

Fig.S13. Mass spectrum of compound 4a7

Fig.S14. ^{1}H NMR And ^{13}C NMR Spectrum of compound 4a7

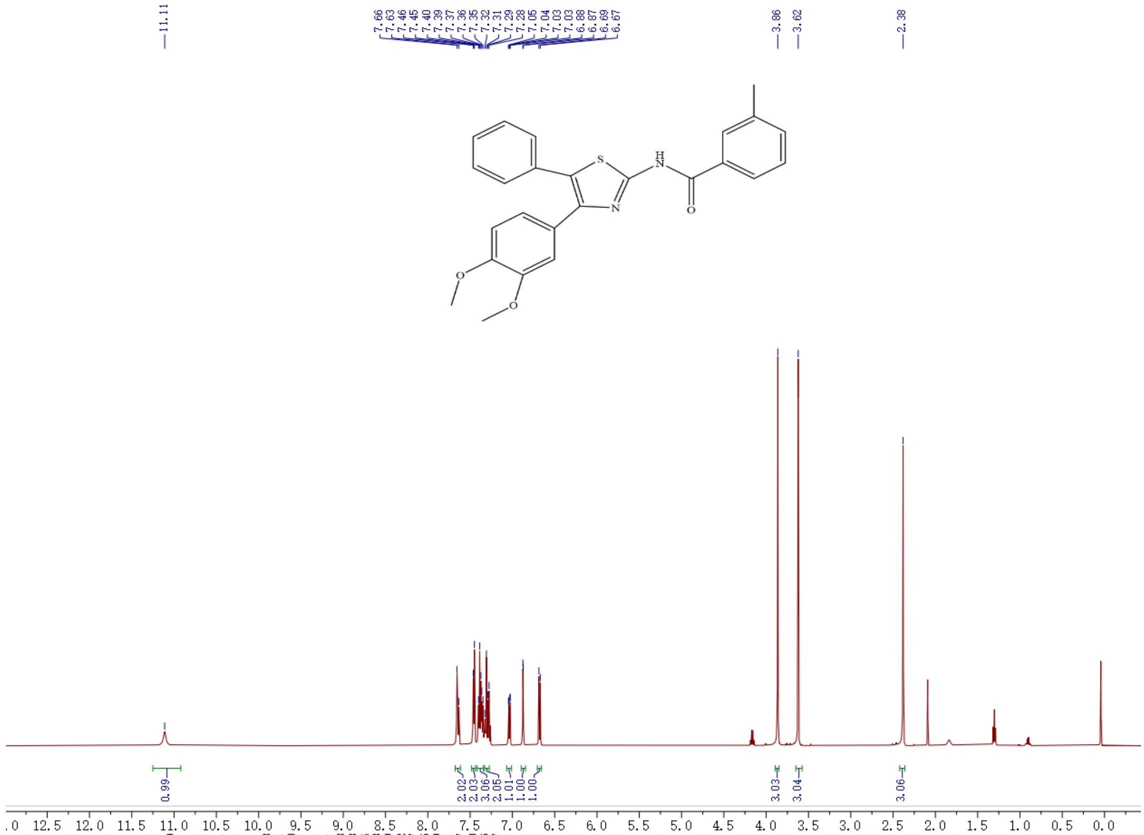

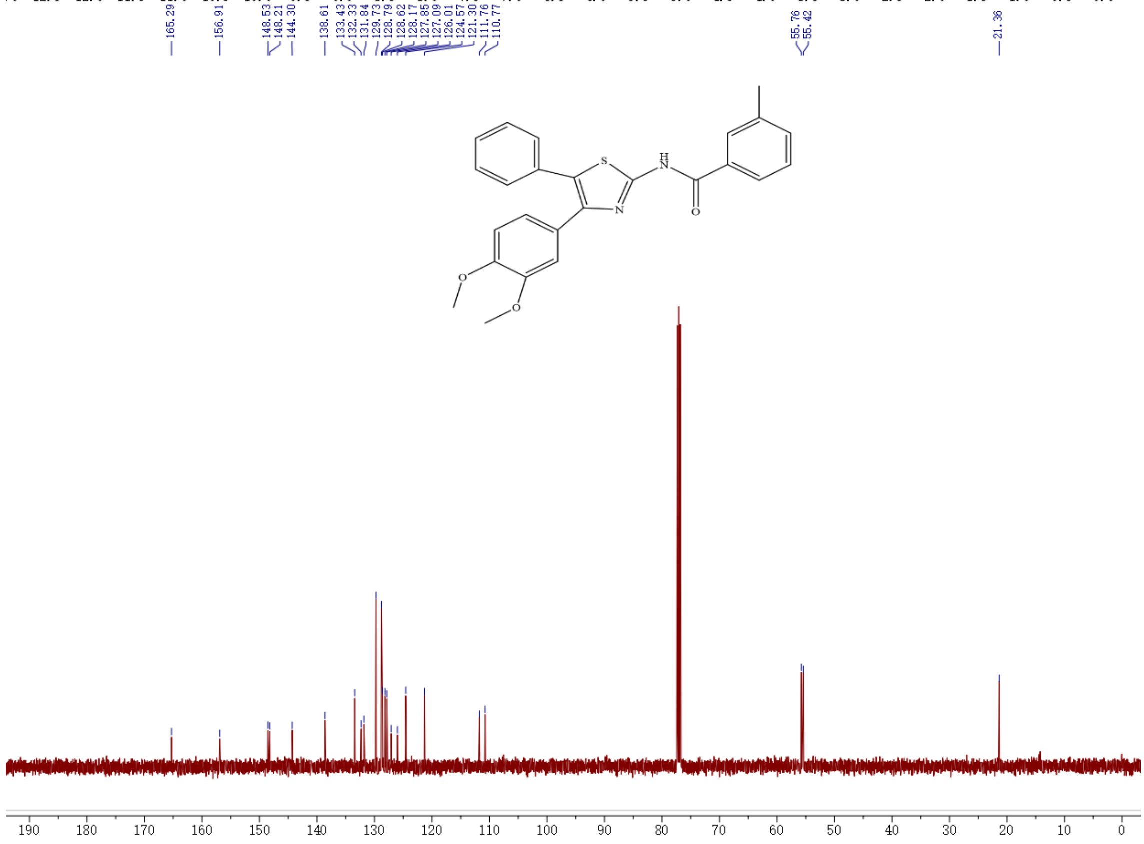

***N*-[4-(3, 4-Dimethoxyphenyl)-5-phenyl-1, 3-thiazol-2-yl]-4-methylbenzamide (4a8)**

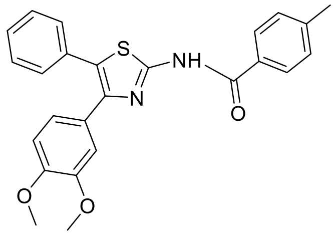

4a8, yield, 64%, white solid, m.p. 135.2-137.1℃. **^{1}H NMR** (500 MHz, $CDCl_3$) δ 11.14 (s, 1H), 7.69 (d, *J* = 8.1 Hz, 2H), 7.40 (d, *J* = 6.5 Hz, 1H), 7.36 – 7.21 (m, 3H), 7.12 (d, *J* = 8.0 Hz, 2H), 6.97 (dd, *J* = 8.2, 2.0 Hz, 1H), 6.82 (d, *J* = 2.0 Hz, 1H), 6.61 (d, *J* = 8.3 Hz, 1H), 3.81 (s, 3H), 3.57 (s, 3H), 2.35 (s, 3H).**^{13}C NMR** (125 MHz, $CDCl_3$) δ 165.1, 157.1, 148.5, 148.3, 144.3, 143.3, 132.4, 129.7, 129.4, 129.0, 128.8, 127.8, 127.5, 127.1, 125.9, 121.5, 111.9, 110.8, 55.8, 55.4, 21.6. HRMS, cald. ($C_{25}H_{22}N_2O_3S$+H) = 431.1429, Found,431.1433.

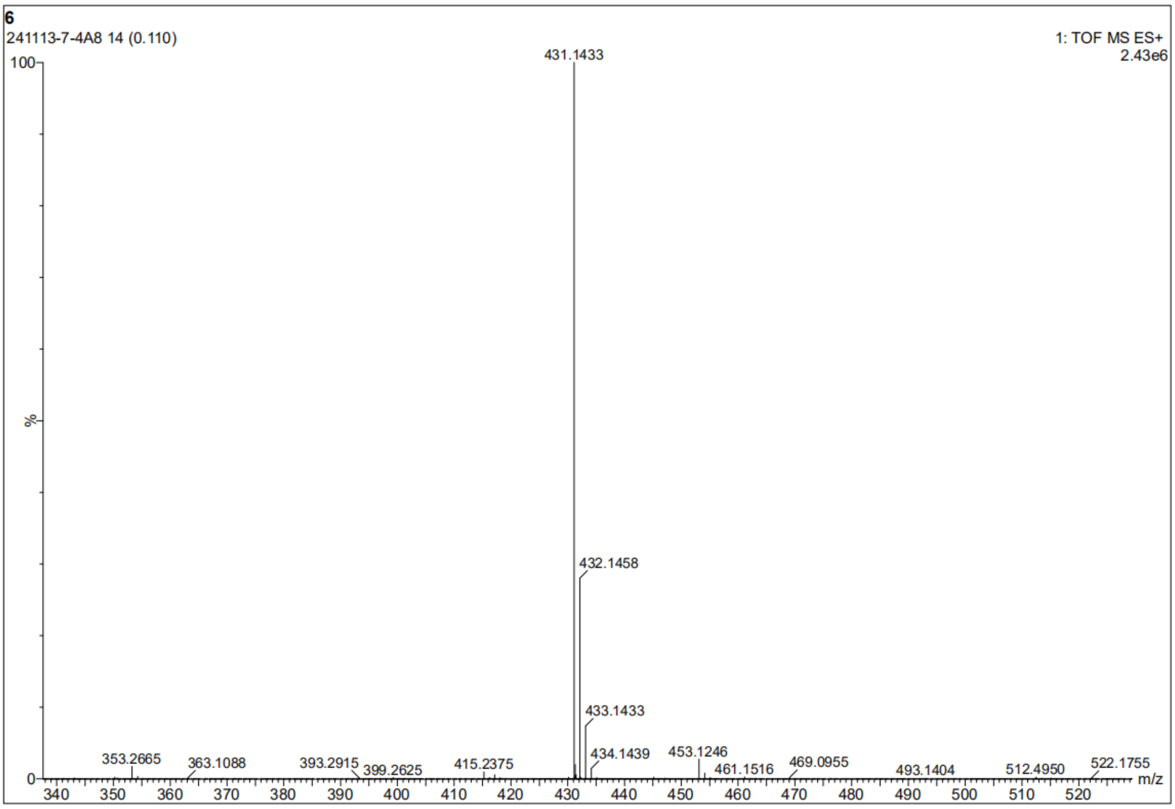

Fig.S15. Mass spectrum of compound 4a8

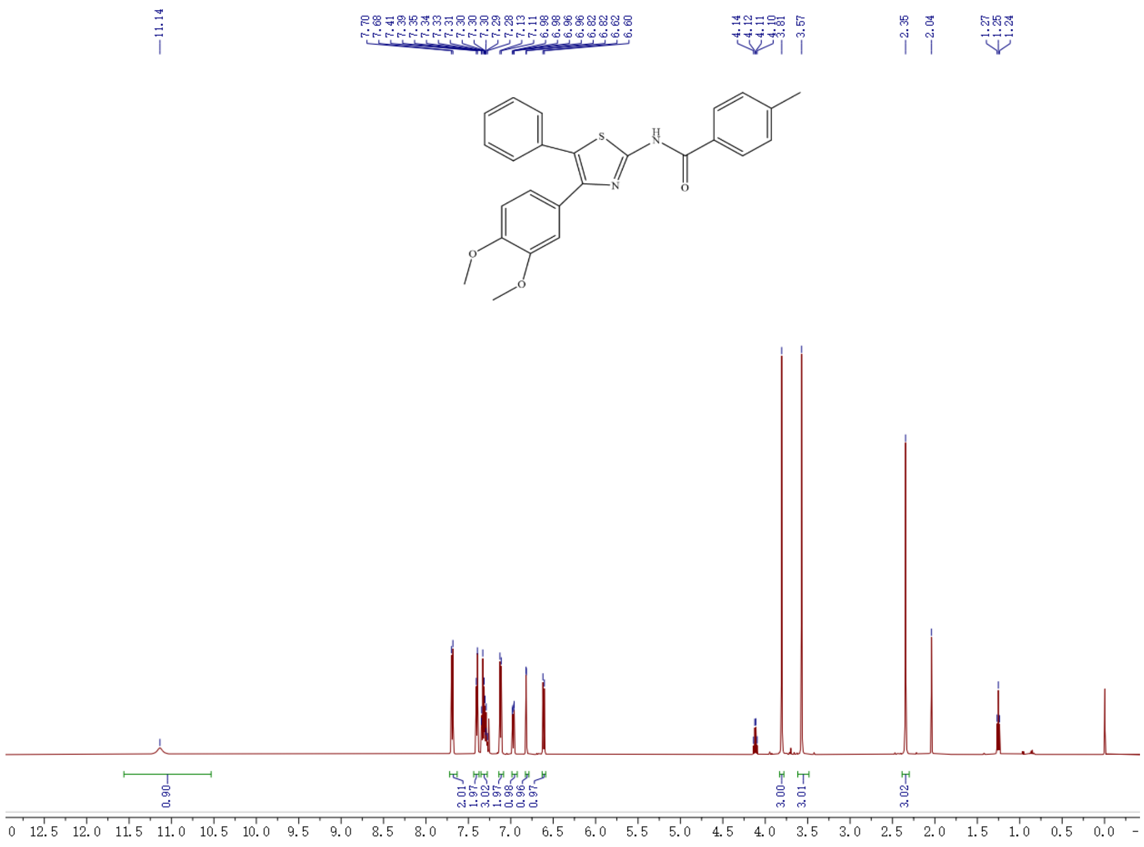
11.14
7.70 7.68 7.41 7.39 7.35 7.34 7.33 7.31 7.30 7.30 7.30 7.29 7.28 7.13 7.11 6.98 6.98 6.96 6.96 6.82 6.82 6.62 6.60
4.14 4.12 4.11 4.10 3.81
3.57
2.35
2.04
1.27 1.25 1.24
S
H
N
N
O
O
O
0.90
2.01 1.97 3.02 1.97 0.98 0.96 0.97
3.00 3.01
3.02
0 12.5 12.0 11.5 11.0 10.5 10.0 9.5 9.0 8.5 8.0 7.5 7.0 6.5 6.0 5.5 5.0 4.5 4.0 3.5 3.0 2.5 2.0 1.5 1.0 0.5 0.0

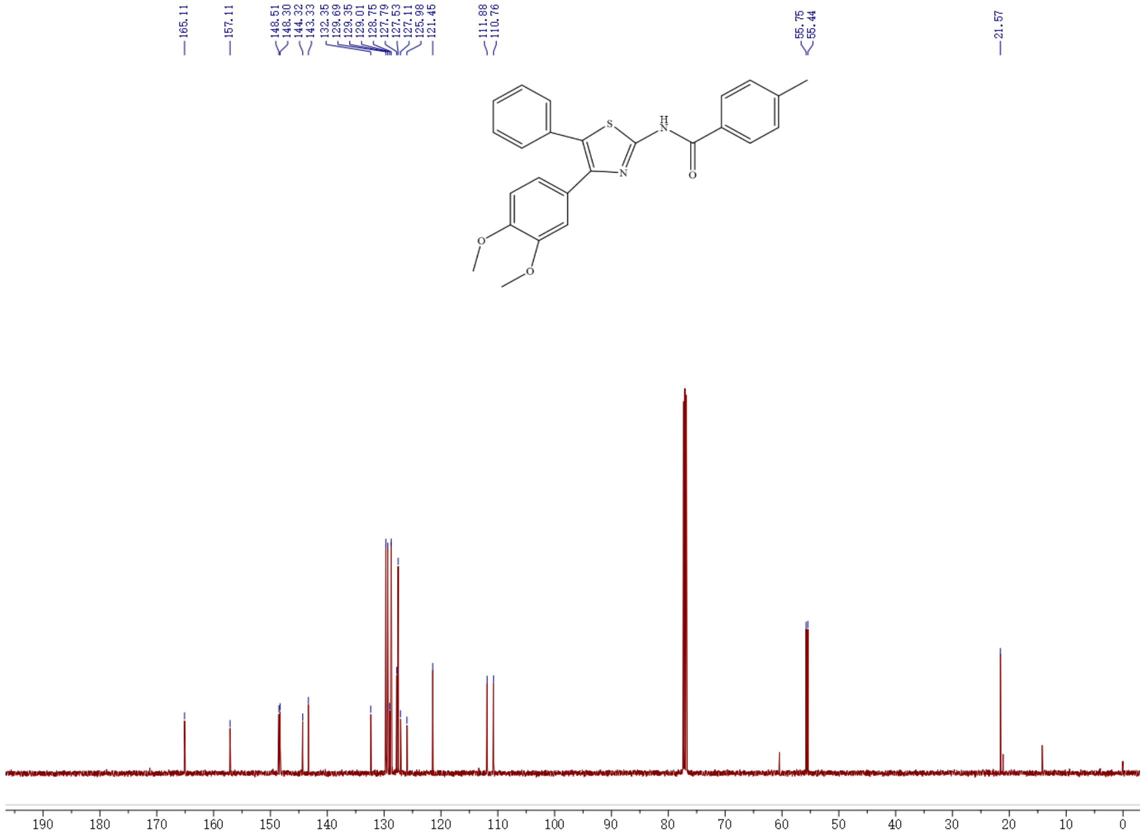
165.11
157.11
148.51 148.30 144.32 143.33
132.35 129.69 129.35 129.01 128.75 127.79 127.53 127.11 125.98 121.45
111.88 110.76
55.75 55.44
21.57
S
H
N
N
O
O
O
190 180 170 160 150 140 130 120 110 100 90 80 70 60 50 40 30 20 10 0

Fig.S16. ^{1}H NMR And ^{13}C NMR Spectrum of compound 4a8

***N*-[4-(3, 4-Dimethoxyphenyl)-5-phenyl-1, 3-thiazol-2-yl]-2-methoxybenzamide (4a9)**

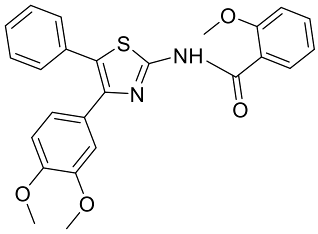

4a9, yield, 65%, white solid, m.p. 101.0-105.2°C. **^{1}H NMR** (500 MHz, $CDCl_3$) δ 11.15 (s, 1H), 8.36 (d, J = 7.6 Hz, 1H), 7.59 (t, J = 7.4 Hz, 1H), 7.44 (d, J = 7.2 Hz, 2H), 7.40 – 7.27 (m, 4H), 7.17 (s, 1H), 7.12 – 7.05 (m, 2H), 6.85 (d, J = 8.3 Hz, 1H), 4.14 (s, 3H), 3.92 (s, 3H), 3.73 (s, 3H).**^{13}C NMR** (125 MHz, $CDCl_3$) δ 162.7, 157.8, 155.7, 148.6, 148.5, 144.6, 134.5, 132.7, 132.6, 129.7, 128.7, 127.8, 127.7, 126.3, 121.8, 121.6, 119.1, 112.1, 111.6, 110.9, 56.4, 55.9, 55.6. HRMS, cald. ($C_{25}H_{22}N_2O_4S$+H) = 447.1379, Found,447.1384.

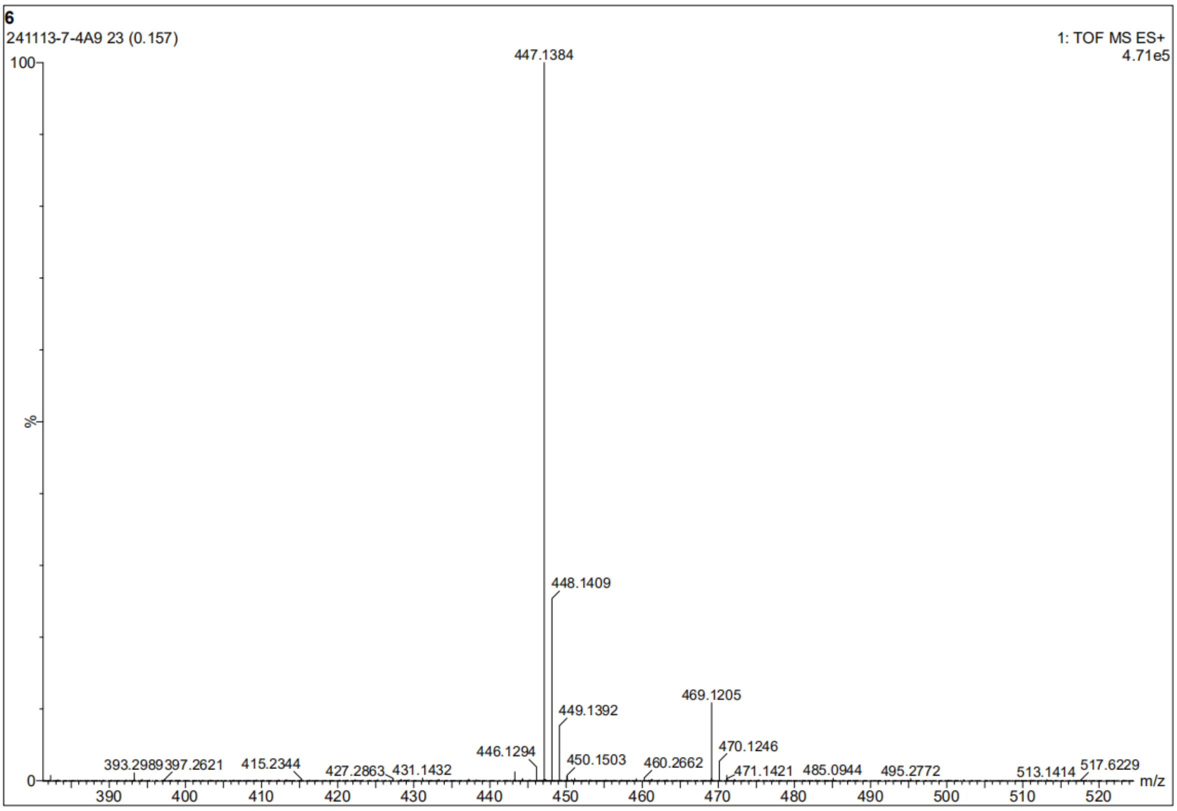

Fig.S17. Mass spectrum of compound 4a9

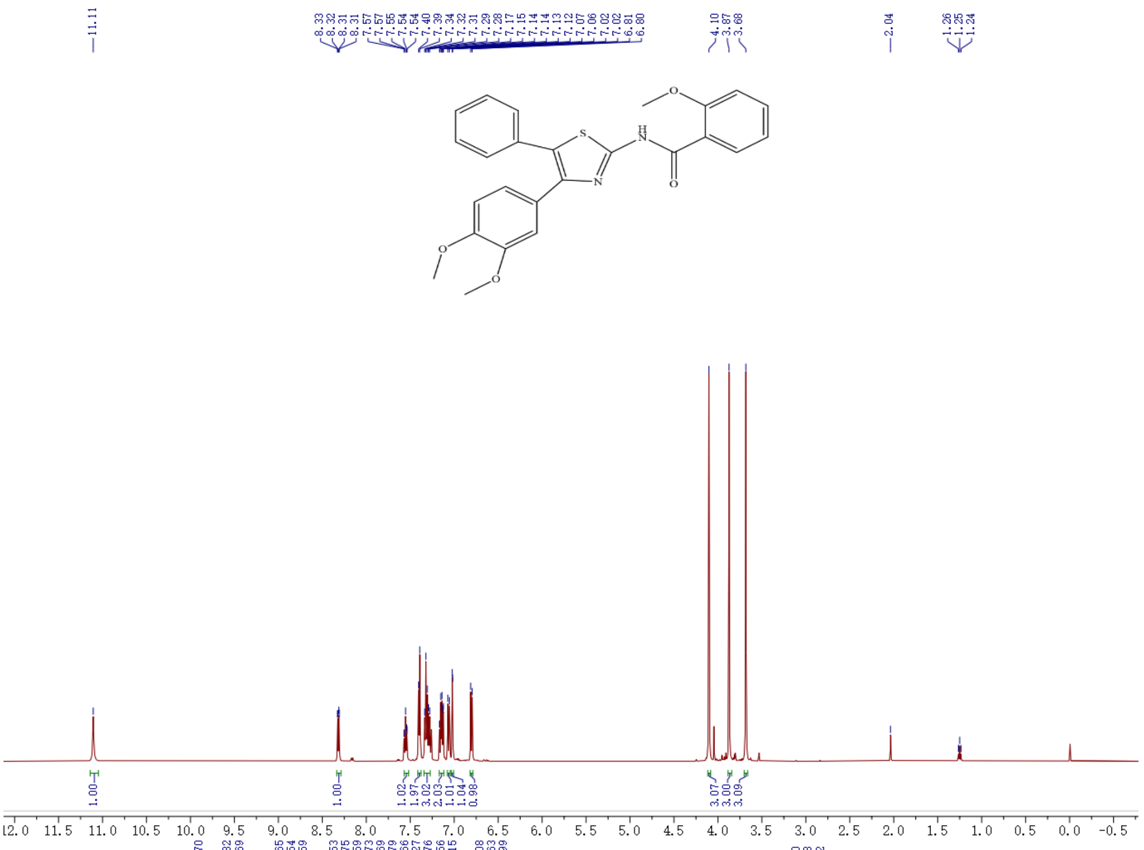

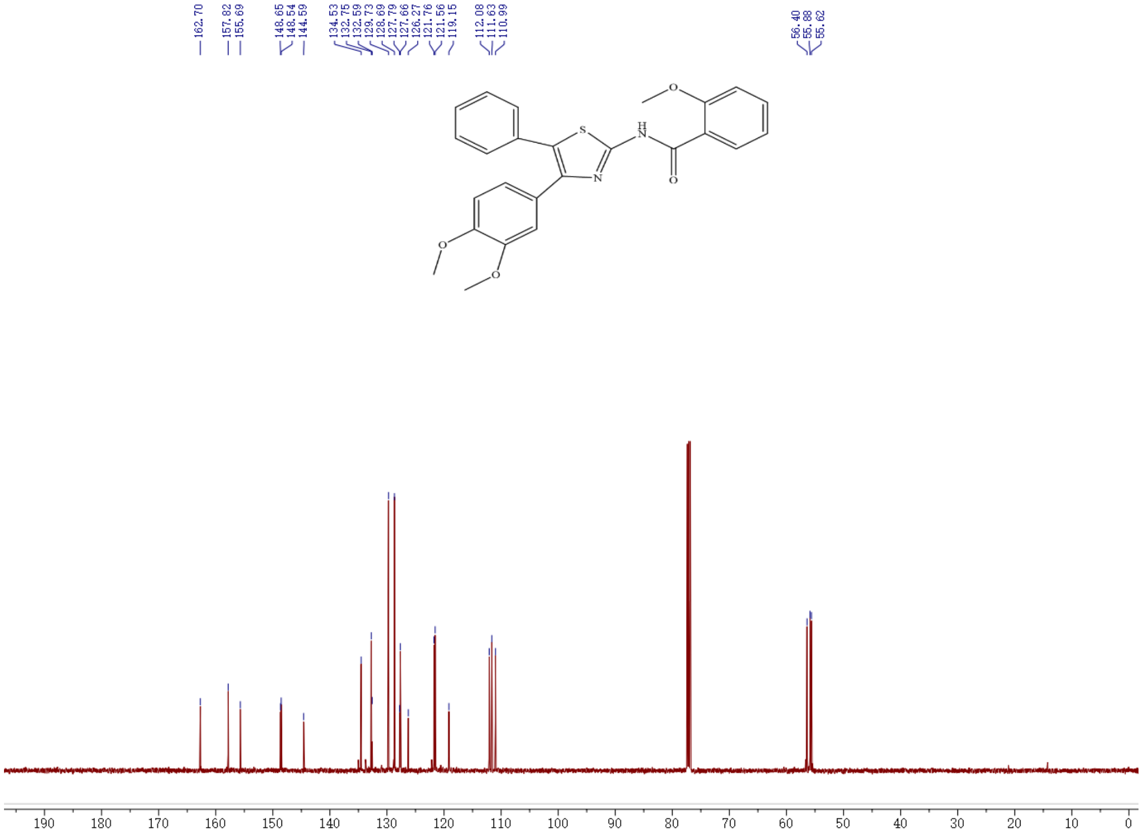

Fig.S18. ^{1}H NMR And ^{13}C NMR Spectrum of compound 4a9

N-[4-(3, 4-Dimethoxyphenyl)-5-phenyl-1, 3-thiazol-2-yl]-4-methoxybenzamide (4a10)

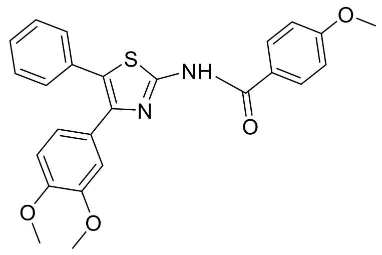

4a10, yield, 68%, white solid, m.p. 168.3-172.2℃. **^{1}H NMR** (500 MHz, $CDCl_3$) δ 11.90 (s, 1H), 7.68 (d, J = 8.5 Hz, 2H), 7.40 (d, J = 6.4 Hz, 2H), 7.32 (t, J = 7.2 Hz, 3H), 6.92 (dd, J = 8.2, 2.0 Hz, 1H), 6.74 (d, J = 1.9 Hz, 1H), 6.70 (d, J = 8.7 Hz, 2H), 6.55 (d, J = 8.3 Hz, 1H), 3.78 (s, 3H), 3.77 (s, 3H), 3.53 (s, 3H).**^{13}C NMR** (125 MHz, $CDCl_3$) δ 165.2, 162.8, 158.0, 148.4, 148.1, 144.3, 132.3, 129.6, 128.8, 127.7, 126.9, 125.7, 124.1, 121.5, 113.7, 111.9, 110.7, 55.7, 55.3, 55.3. HRMS, cald. ($C_{25}H_{22}N_2O_4S$+H) = 447.1379, Found,447.1383.

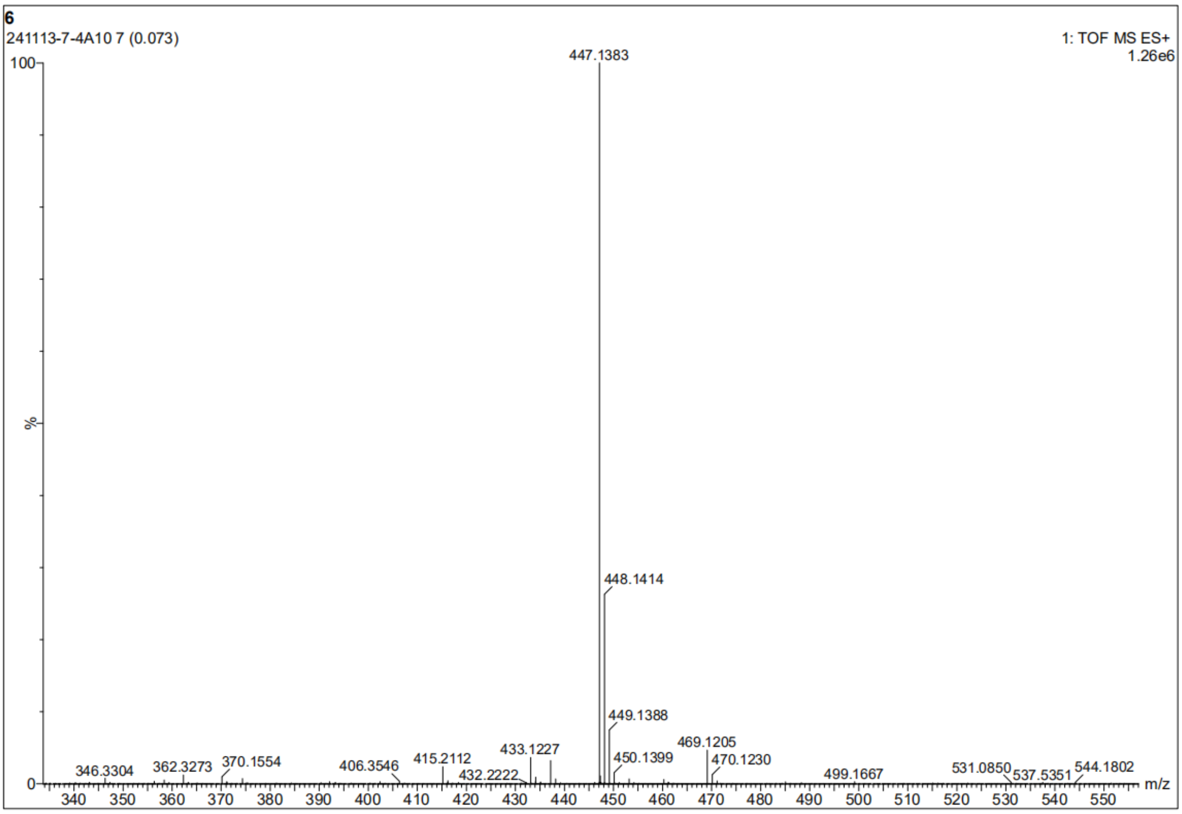

Fig.S19. Mass spectrum of compound 4a10

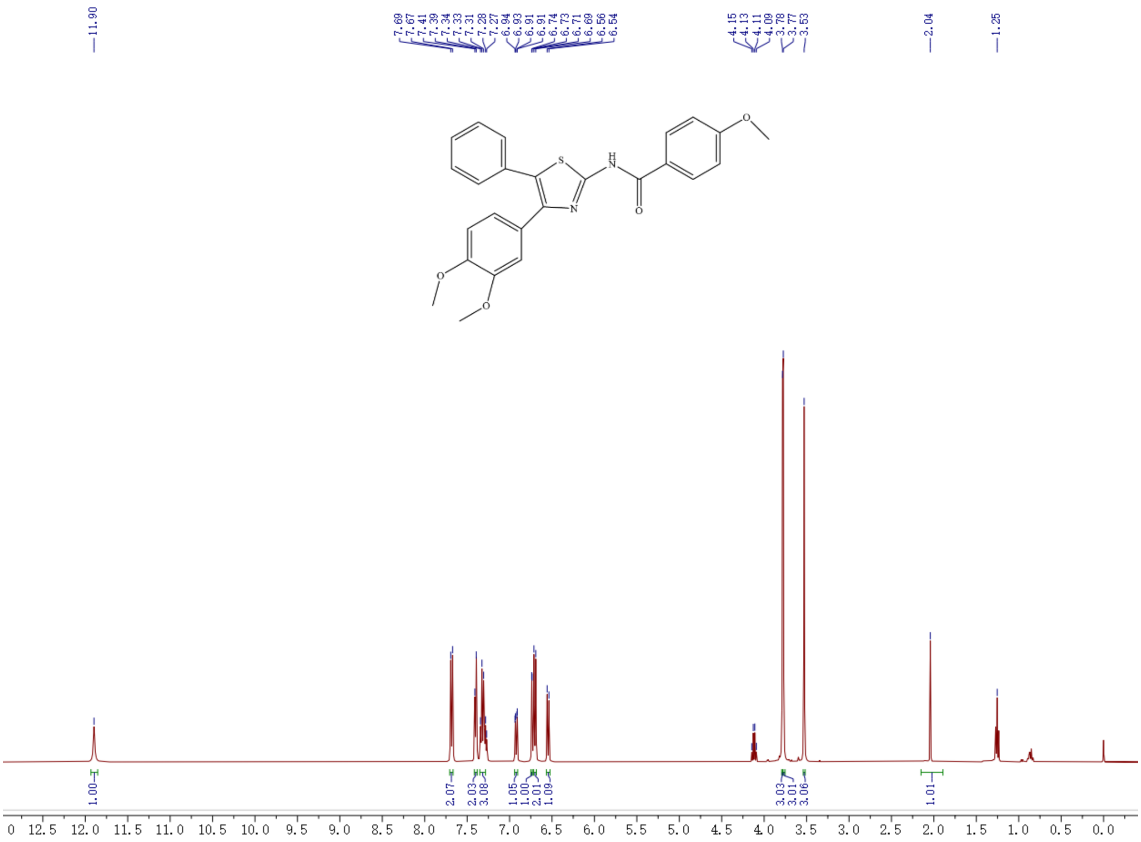

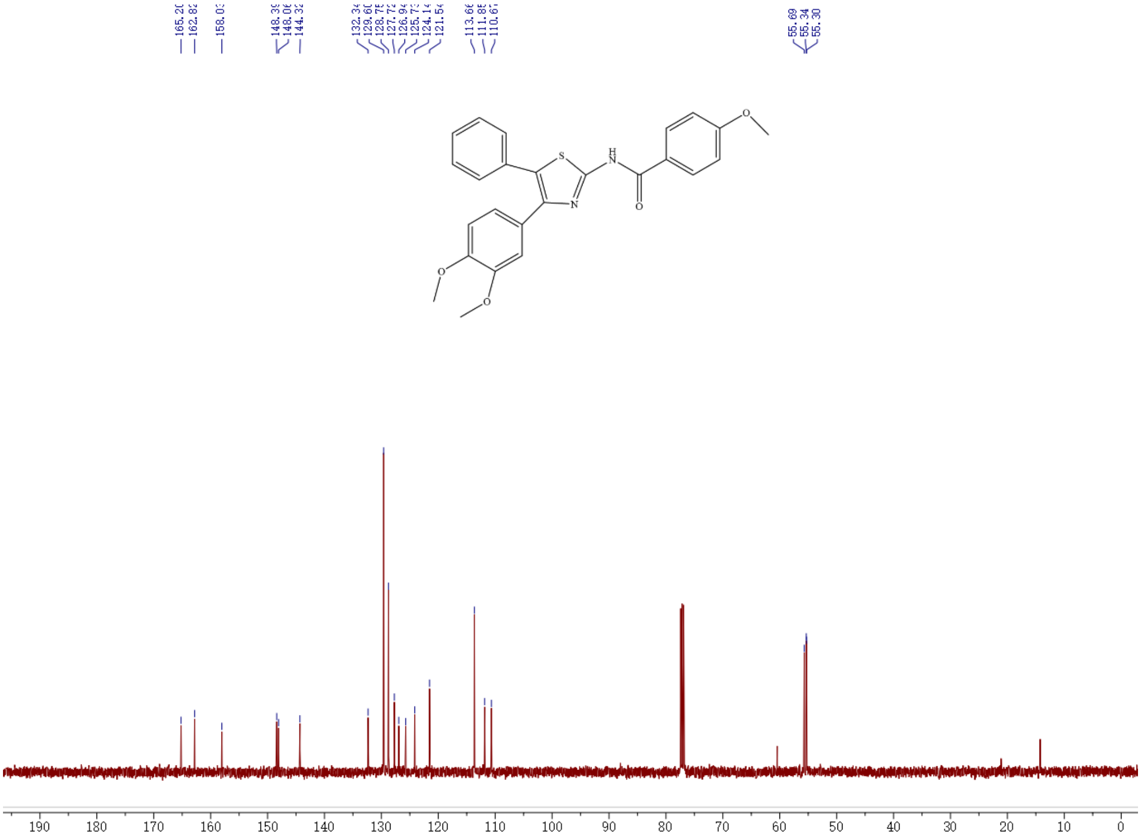

Fig.S20. 1H NMR And 13C NMR Spectrum of compound 4a10

N-[4-(3, 4-Dimethoxyphenyl)-5-phenyl-1, 3-thiazol-2-yl]-2-fluorobenzamide (4a11)

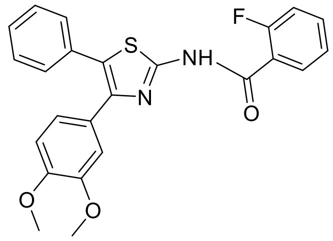

4a11, yield, 68%, white solid, m.p. 119.9-121.3℃. **^{1}H NMR** (500 MHz, $CDCl_3$) δ 10.16 (s, 1H), 8.24 (t, J = 7.8 Hz, 1H), 7.61 (d, J = 7.9 Hz, 1H), 7.45 (d, J = 6.8 Hz, 2H), 7.38 (dd, J = 12.5, 5.1 Hz, 4H), 7.27 – 7.23 (m, 1H), 7.14 (d, J = 8.3 Hz, 1H), 7.04 (d, J = 1.8 Hz, 1H), 6.82 (d, J = 8.4 Hz, 1H), 3.91 (s, 3H), 3.71 (s, 3H).**^{13}C NMR** (125 MHz, $CDCl_3$) δ 161.7, 160.6 (d, J = 2.9 Hz), 159.7, 155.1, 148.7, 148.6, 144.6, 134.9 (d, J = 9.4 Hz), 132.3 (d, J = 7.5 Hz), 129.8, 128.7, 127.9, 127.5, 126.6, 125.3 (d, J = 3.0 Hz), 121.4, 119.1 (d, J = 10.7 Hz), 116.4 (d, J = 24.3 Hz), 112.1, 111.0, 55.9, 55.6. HRMS, cald. ($C_{24}H_{19}FN_2O_3S$+H) = 435.1179, Found,435.1183.

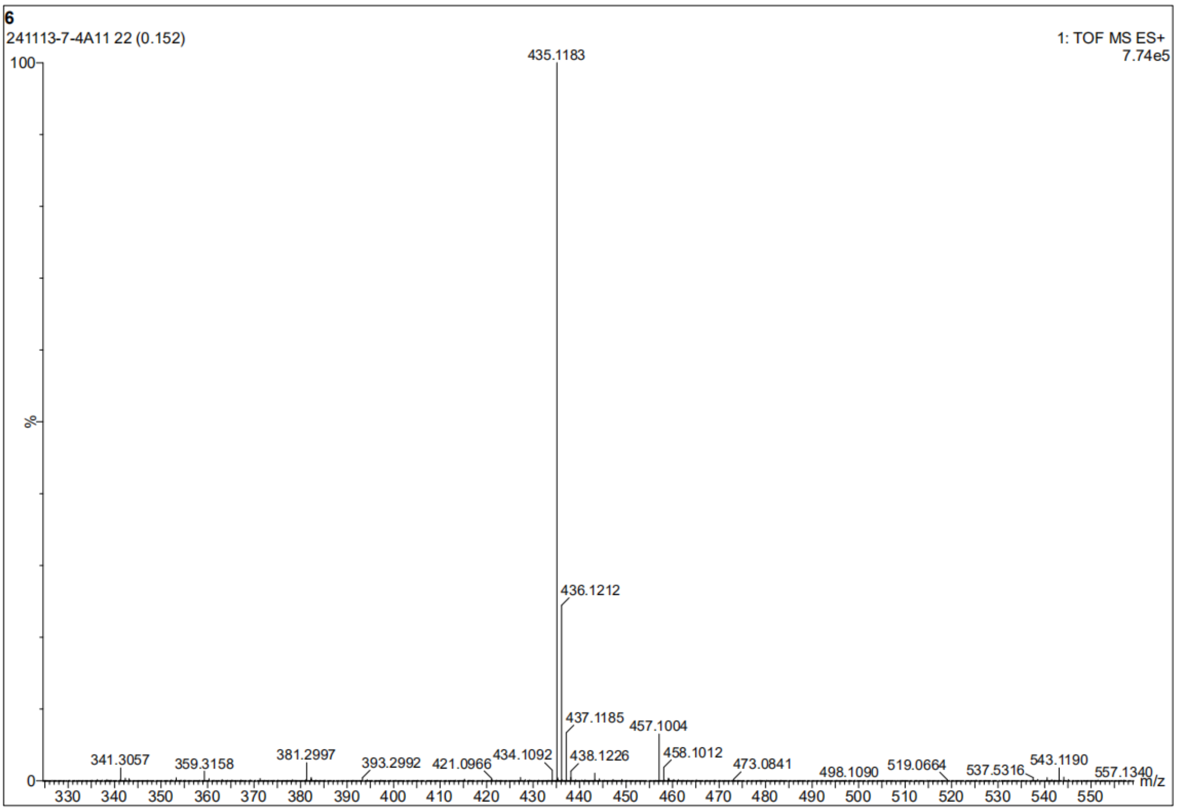

Fig.S21. Mass spectrum of compound 4a11

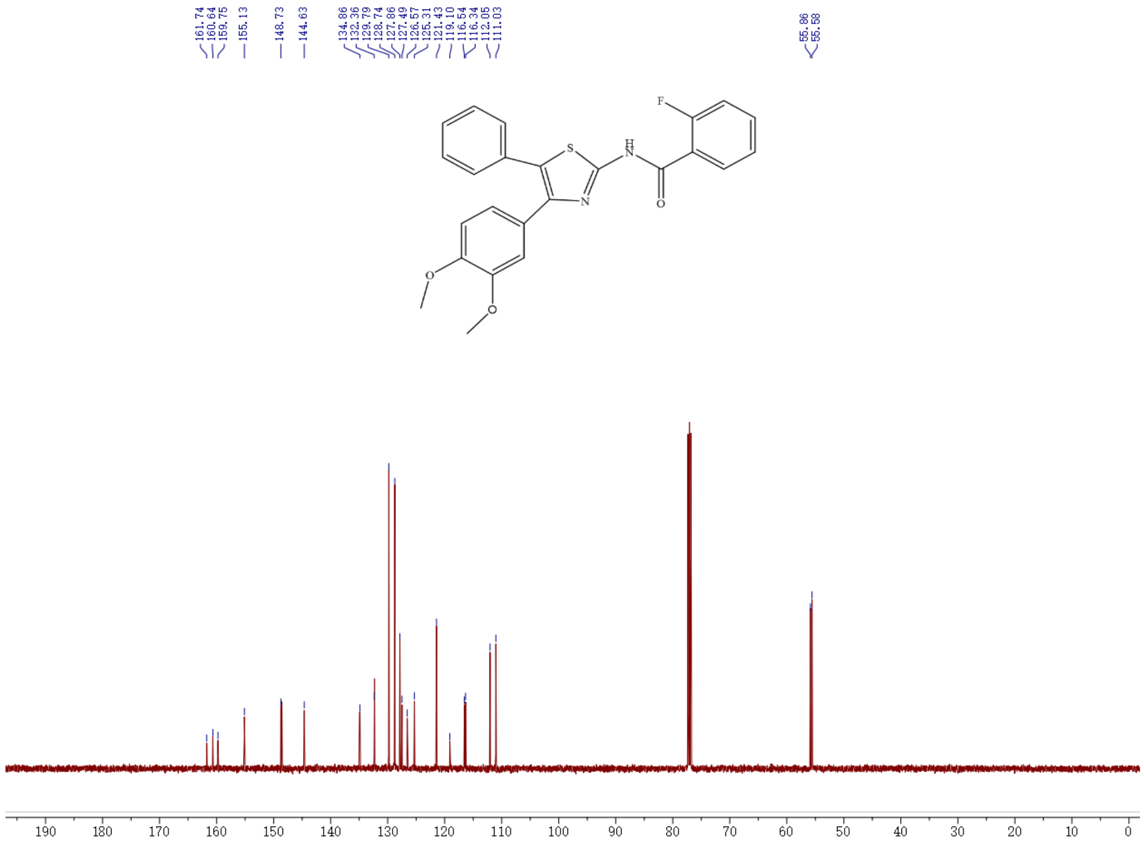

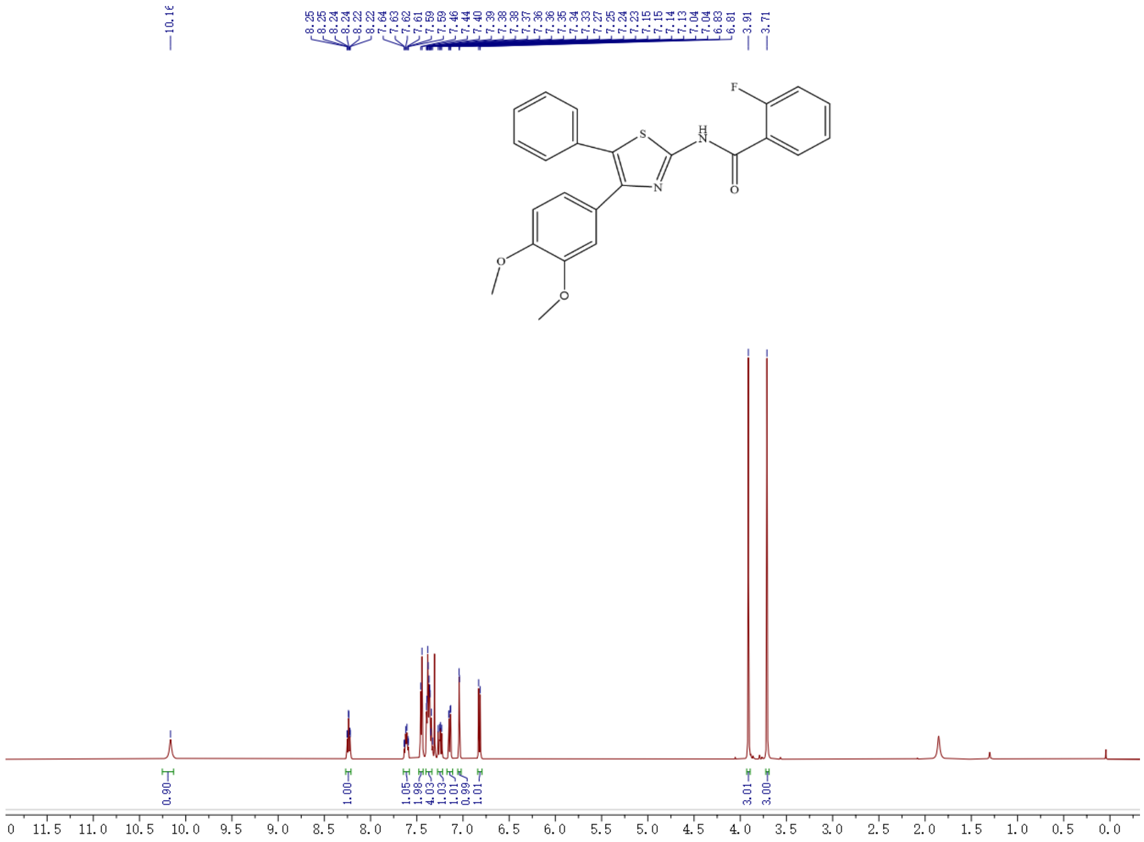

Fig.S22. 1H NMR And 13C NMR Spectrum of compound 4a11

***N*-[4-(3, 4-Dimethoxyphenyl)-5-phenyl-1, 3-thiazol-2-yl]-3-fluorobenzamide (4a12)**

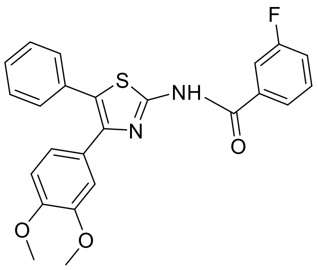

4a12, yield, 66%, white solid, m.p. 173.6-175.8℃. **^{1}H NMR** (500 MHz, $CDCl_3$) δ 12.08 (s, 1H), 7.55 (d, *J* = 7.8 Hz, 1H), 7.45 (d, *J* = 6.5 Hz, 3H), 7.41 – 7.34 (m, 3H), 7.29 – 7.23 (m, 1H), 7.12 (td, *J* = 8.3, 2.6 Hz, 1H), 6.94 (dd, *J* = 8.3, 2.0 Hz, 1H), 6.81 (d, *J* = 2.0 Hz, 1H), 6.58 (d, *J* = 8.4 Hz, 1H), 3.82 (s, 3H), 3.59 (s, 3H). **^{13}C NMR** (125 MHz, $CDCl_3$) δ 164.3 (d, *J* = 2.5 Hz), 163.4, 161.4, 157.5, 148.6, 148.2, 144.4, 134.1 (d, *J* = 7.0 Hz), 132.1, 130.1 (d, *J* = 7.9 Hz), 129.7, 128.8, 127.9, 126.8, 126.3, 123.2 (d, *J* = 3.0 Hz), 121.4, 119.4 (d, *J* = 21.2 Hz), 114.8 (d, *J* = 23.3 Hz), 111.9, 110.9, 55.7, 55.4. HRMS, cald. ($C_{24}H_{19}FN_2O_3S$+H) = 435.1179, Found, 435.1181.

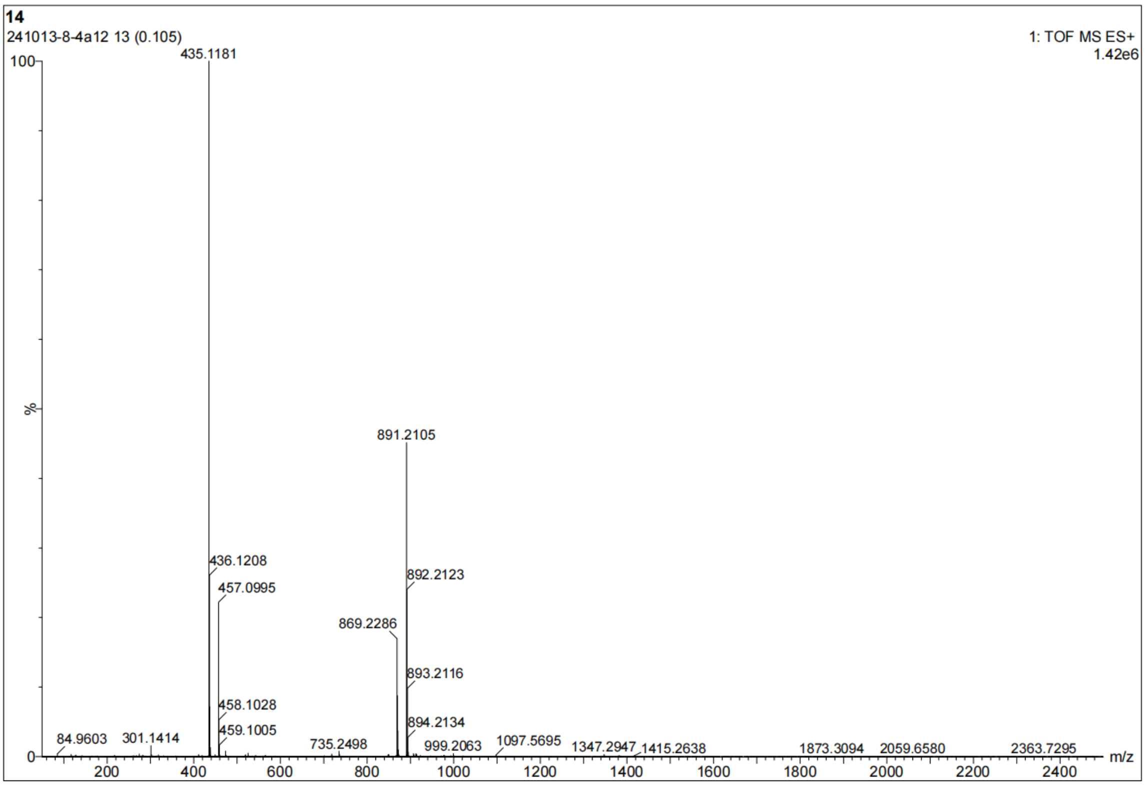

Fig.S23. Mass spectrum of compound 4a12

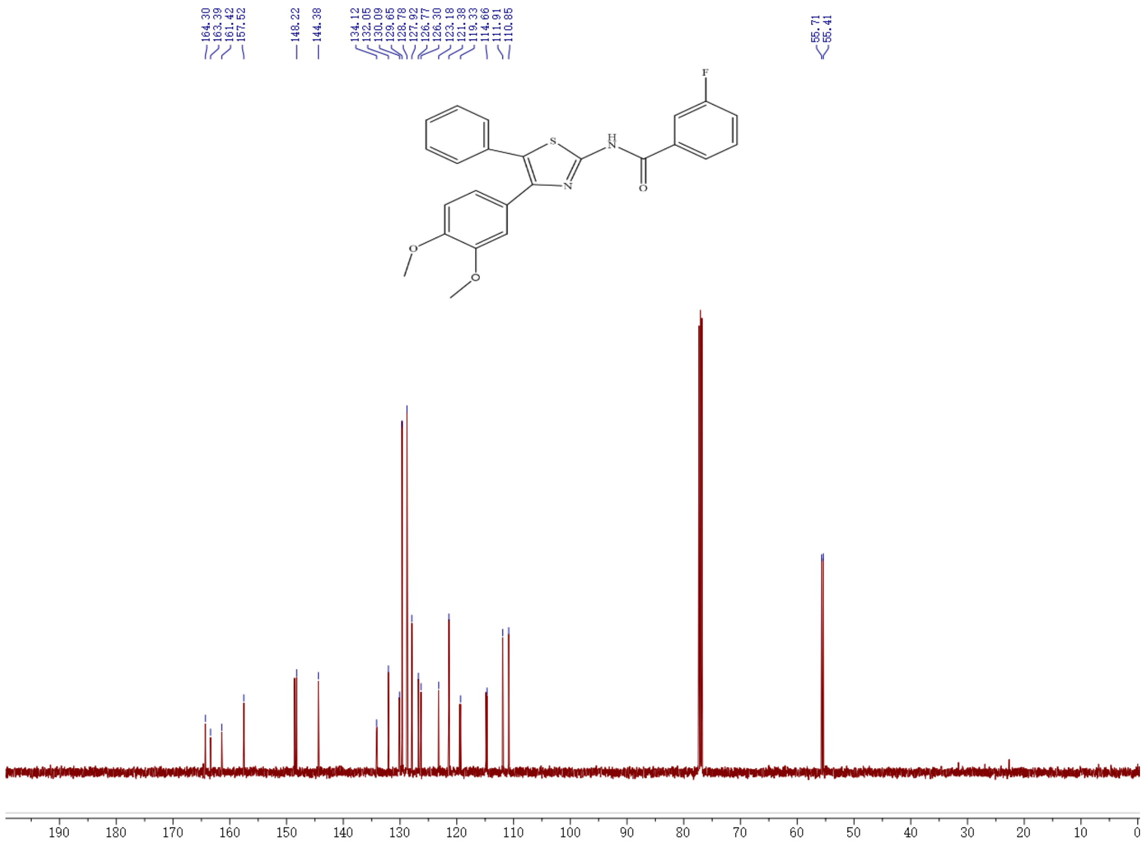

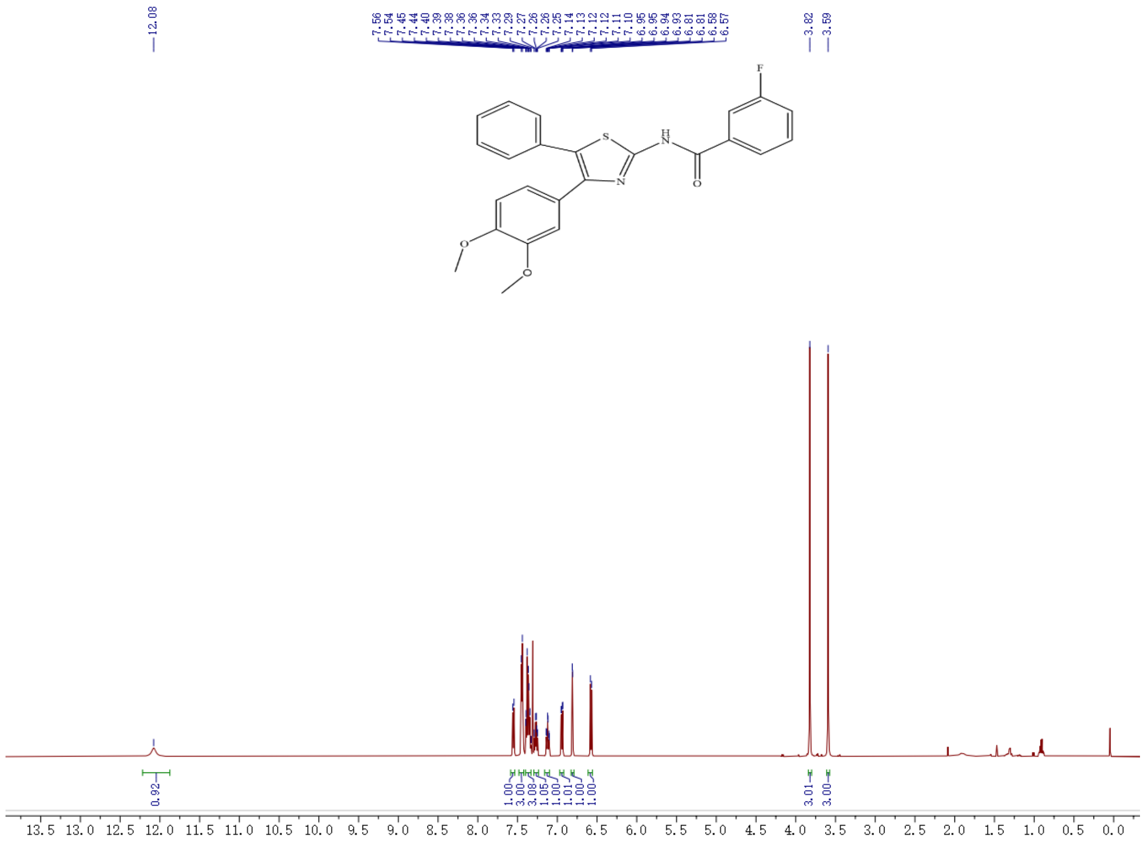

Fig.S24. 1H NMR And 13C NMR Spectrum of compound 4a12

_N_-[4-(3, 4-Dimethoxyphenyl)-5-phenyl-1, 3-thiazol-2-yl]-3-chlorobenzamide (4a13)

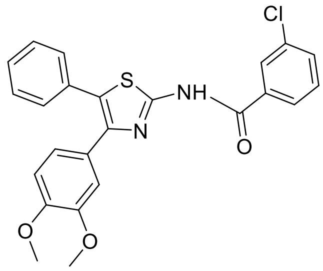

4a13, yield, 64%, white solid, m.p. 131.8-136.4℃. **^{1}H NMR** (500 MHz, $CDCl_3$) δ 12.17 (s, 1H), 7.71 (s, 1H), 7.66 (d, *J* = 7.8 Hz, 1H), 7.45 (d, *J* = 6.7 Hz, 2H), 7.40 – 7.33 (m, 4H), 7.23 (t, *J* = 7.9 Hz, 1H), 6.96 (d, *J* = 10.1 Hz, 1H), 6.81 (s, 1H), 6.58 (d, *J* = 8.4 Hz, 1H), 3.83 (s, 3H), 3.58 (s, 3H). **^{13}C NMR** (125 MHz, $CDCl_3$) δ 164.3, 157.5, 148.6, 148.1, 144.3, 134.7, 133.7, 132.4, 132.1, 129.8, 129.7, 128.8, 127.9, 127.8, 126.7, 126.3, 125.7, 121.3, 111.9, 110.8, 55.7, 55.4. HRMS, cald. ($C_{24}H_{19}ClN_2O_3S$+H) = 451.0883

, Found, 451.0888.

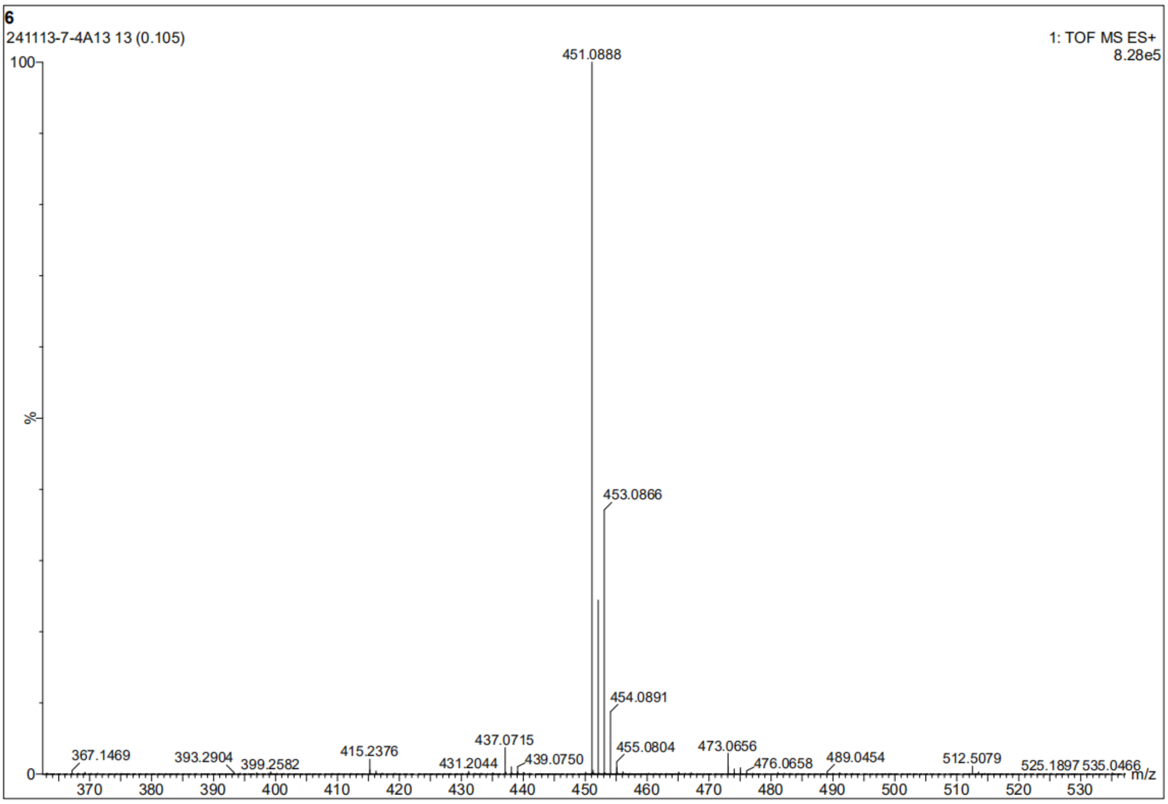

Fig.S25. Mass spectrum of compound 4a13

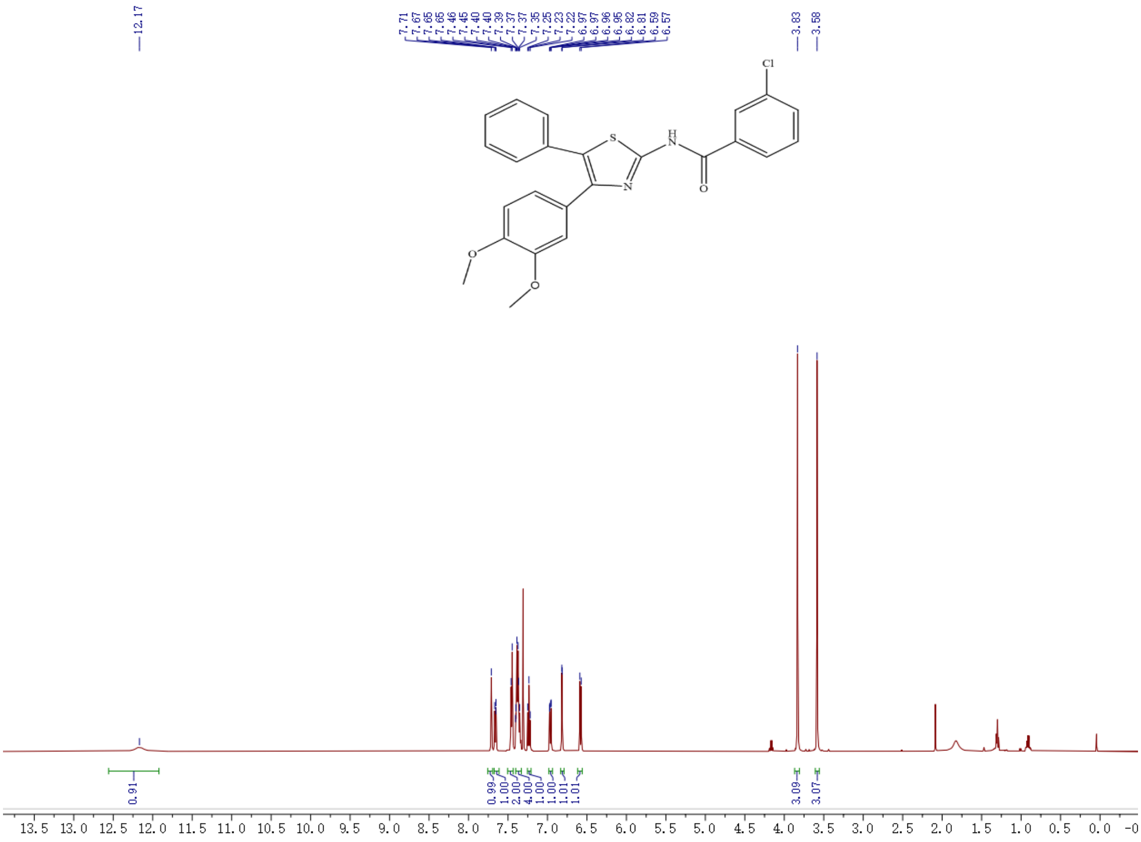
12.17
7.71 7.67 7.65 7.65 7.46 7.45 7.40 7.40 7.39 7.37 7.37 7.35 7.25 7.23 7.22 6.97 6.97 6.96 6.95 6.82 6.81 6.59 6.57
3.83 3.58
Cl
S
H
N
N
O
O
O
0.91
0.99 1.00 2.00 4.00 1.00 1.00 1.01 1.01
3.09 3.07
13.5 13.0 12.5 12.0 11.5 11.0 10.5 10.0 9.5 9.0 8.5 8.0 7.5 7.0 6.5 6.0 5.5 5.0 4.5 4.0 3.5 3.0 2.5 2.0 1.5 1.0 0.5 0.0 -0

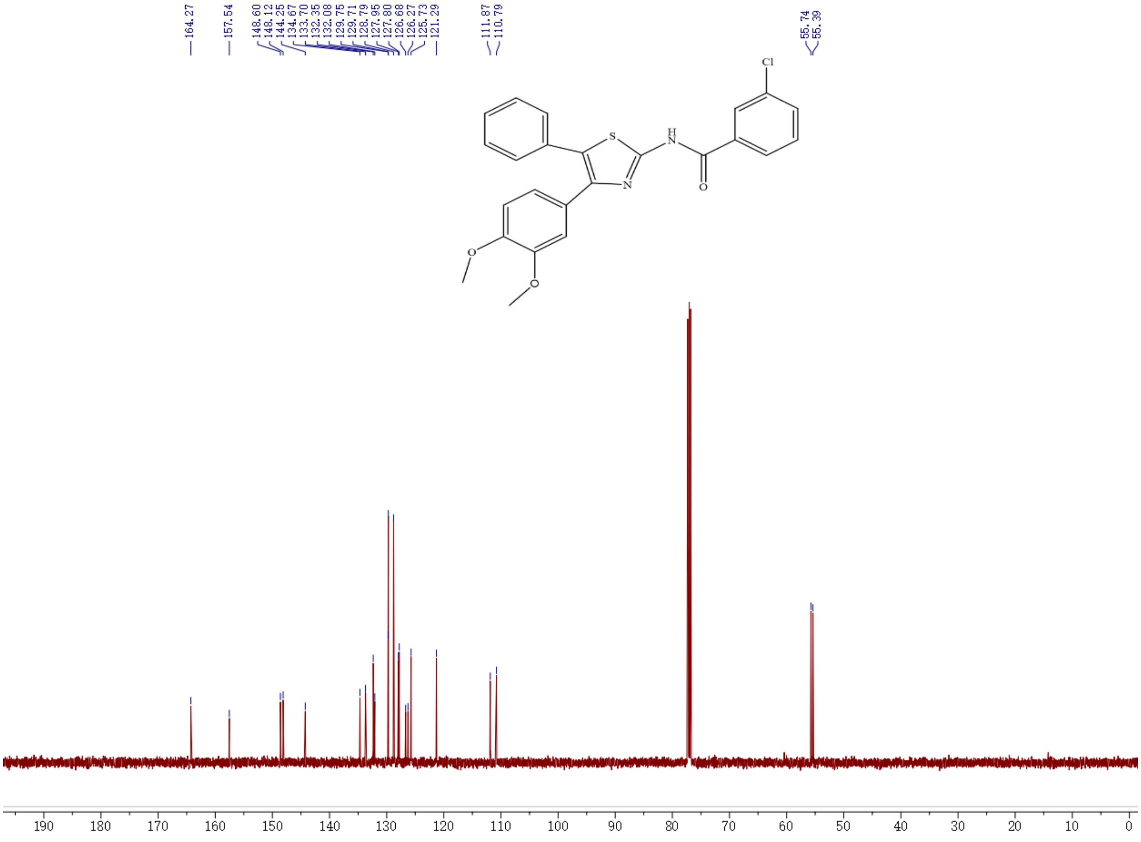
164.27
157.54
148.60 148.12 144.25 134.67 133.70 132.35 132.08 129.75 129.71 128.79 127.95 127.80 126.68 126.27 125.73 121.29
111.87 110.79
55.74 55.39
Cl
S
H
N
N
O
O
O
190 180 170 160 150 140 130 120 110 100 90 80 70 60 50 40 30 20 10 0

Fig.S26. 1H NMR And 13C NMR Spectrum of compound 4a13

***N*-[4-(3, 4-Dimethoxyphenyl)-5-phenyl-1, 3-thiazol-2-yl]-4-chlorobenzamide (4a14)**

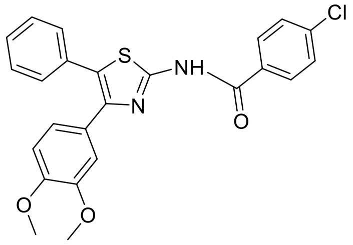

4a14, yield, 64%, white solid, m.p. 162.0-162.6℃. ^{1}**H NMR** (500 MHz, $CDCl_3$) δ 11.81 (s, 1H), 7.73 (d, *J* = 8.5 Hz, 2H), 7.43 (d, *J* = 7.7 Hz, 2H), 7.36 (dd, *J* = 15.4, 7.7 Hz, 3H), 7.24 (d, *J* = 8.5 Hz, 2H), 6.89 (d, *J* = 8.3 Hz, 1H), 6.78 (d, *J* = 1.8 Hz, 1H), 6.61 (d, *J* = 8.4 Hz, 1H), 3.85 (s, 3H), 3.61 (s, 3H).^{13}**C NMR** (125 MHz, $CDCl_3$) δ 164.8, 158.0, 148.5, 148.2, 144.3, 138.7, 131.9, 130.2, 129.6, 129.0, 128.8, 128.7, 127.9, 126.5, 126.2, 121.7, 111.6, 110.6, 55.7, 55.4. HRMS, cald. ($C_{24}H_{19}ClN_2O_3S$+H) = 451.0883, Found, 451.0884.

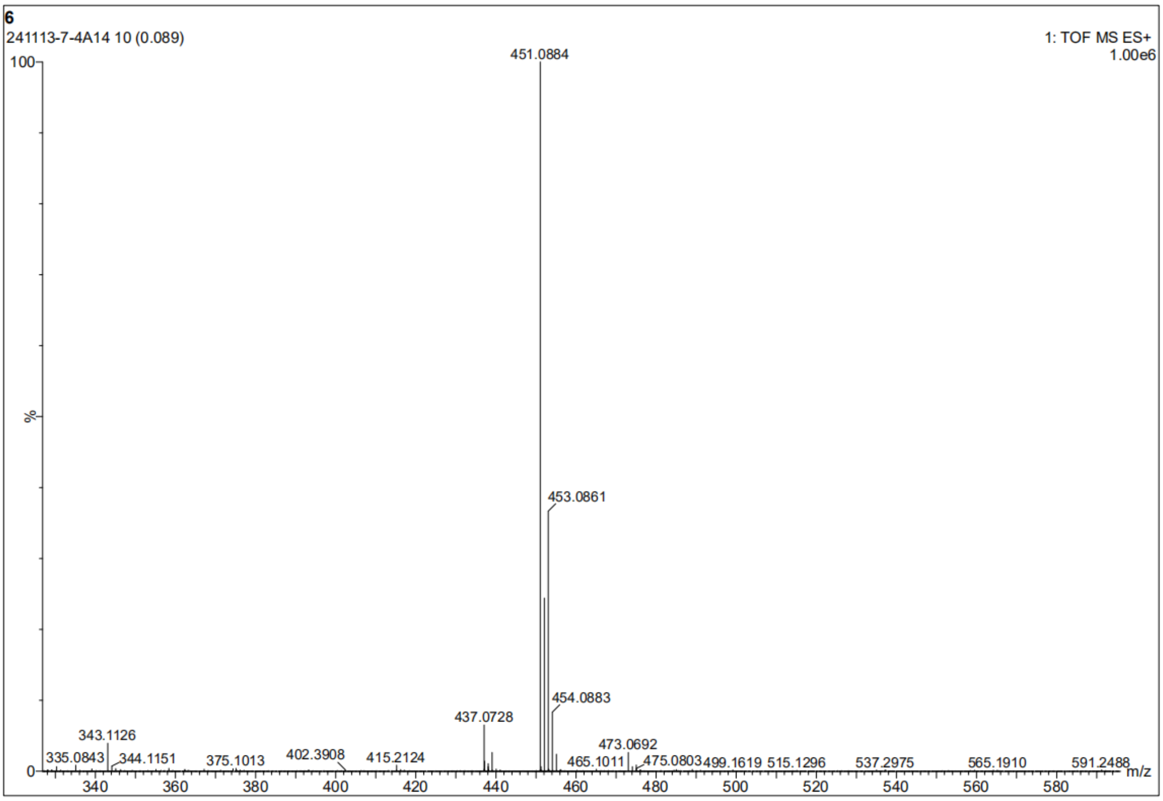

Fig.S27. Mass spectrum of compound 4a14

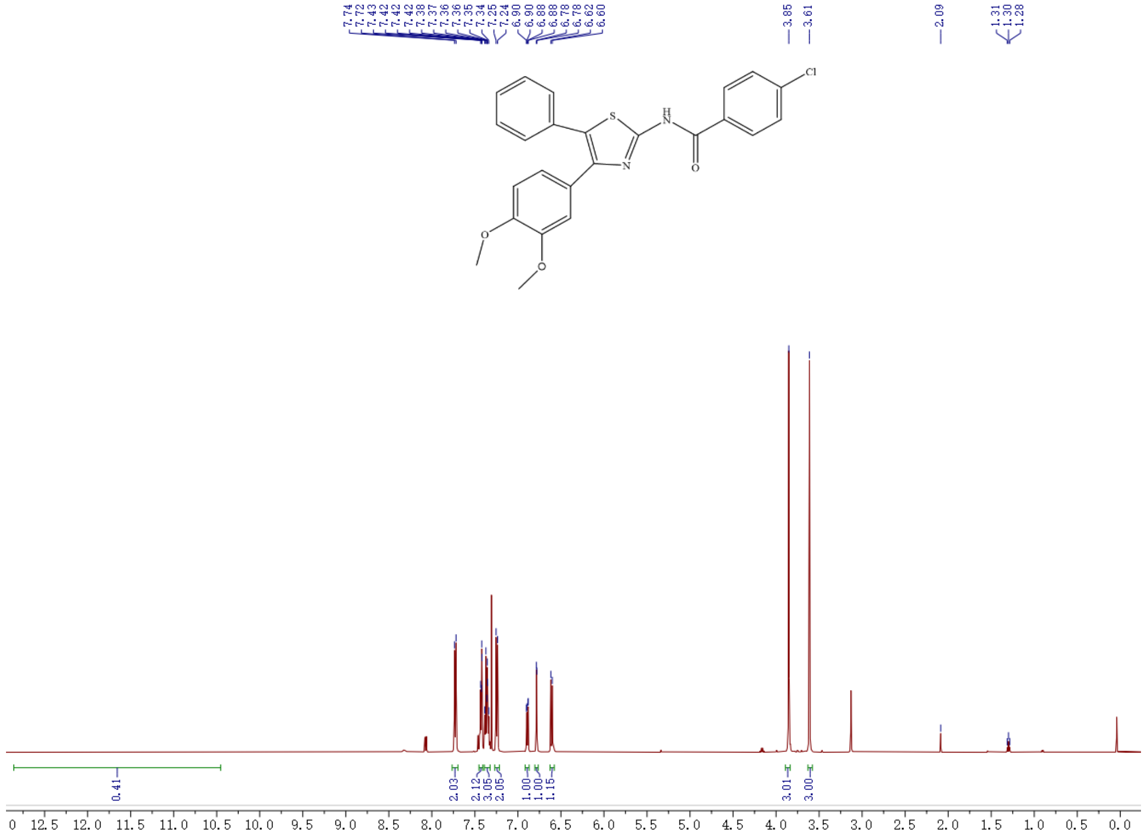

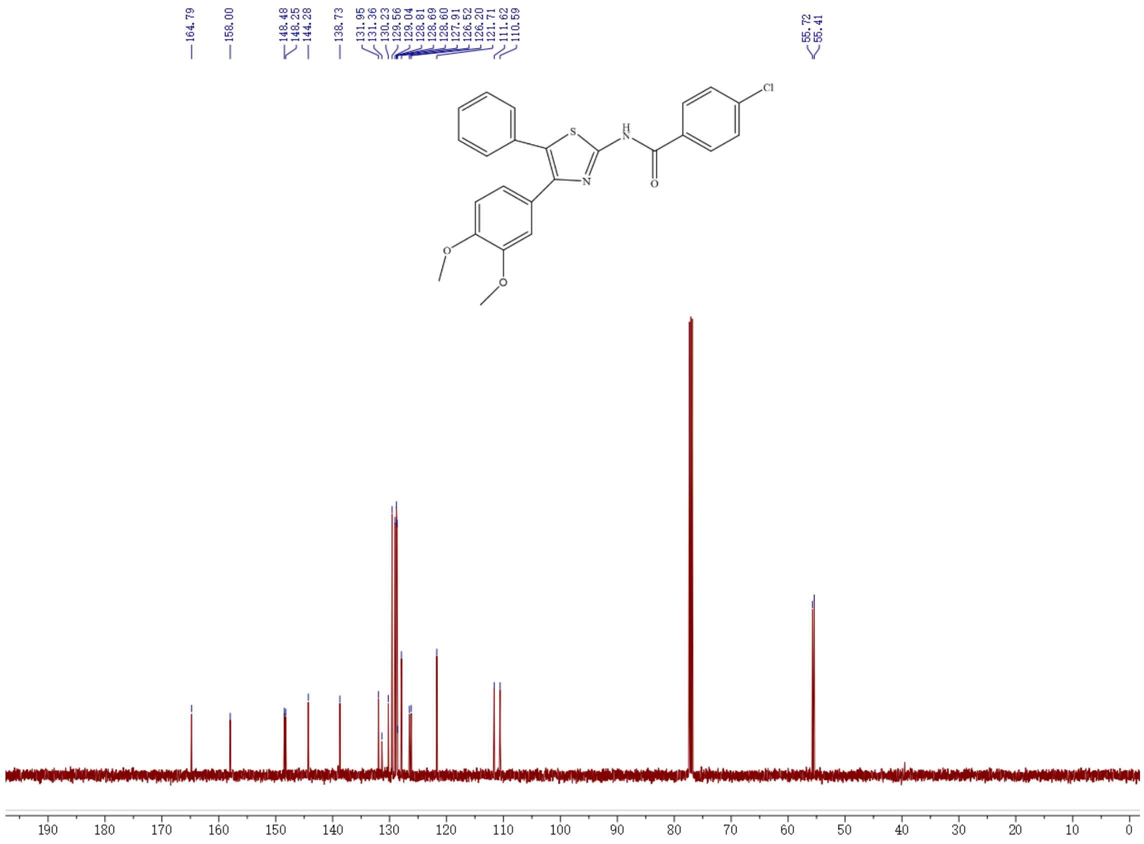

Fig.S28. 1H NMR And 13C NMR Spectrum of compound 4a14

_N_-[4-(3, 4-Dimethoxyphenyl)-5-phenyl-1, 3-thiazol-2-yl]-4-iodobenzamide (4a15)

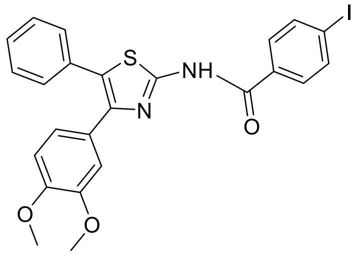

4a15, yield, 63%, white solid, m.p. 172.5-175.9℃. **^{1}H NMR** (500 MHz, $CDCl_3$) δ 12.38 (s, 1H), 7.59 (d, J = 7.8 Hz, 2H), 7.45 – 7.36 (m, 7H), 6.87 (d, J = 7.4 Hz, 1H), 6.78 (s, 1H), 6.60 (d, J = 8.1 Hz, 1H), 3.86 (s, 3H), 3.61 (s, 3H). **^{13}C NMR** (125 MHz, $CDCl_3$) δ 165.1, 157.8, 148.5, 148.3, 144.4, 137.6, 132.0, 131.2, 129.6, 129.0, 128.8, 127.9, 126.6, 126.3, 121.7, 111.6, 110.7, 100.1, 55.8, 55.5. HRMS, cald. ($C_{24}H_{19}IN_2O_3S$+H) = 543.0239, Found, 543.0244.

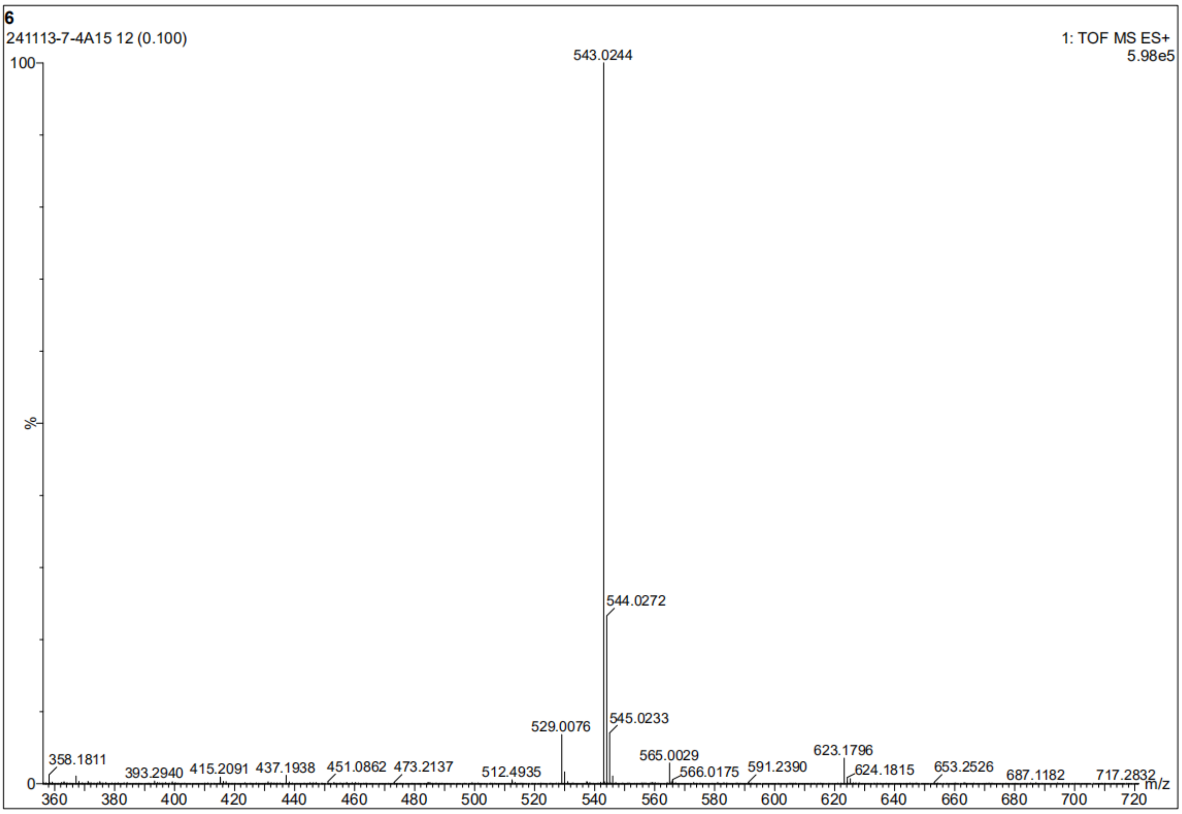

Fig.S29. Mass spectrum of compound 4a15

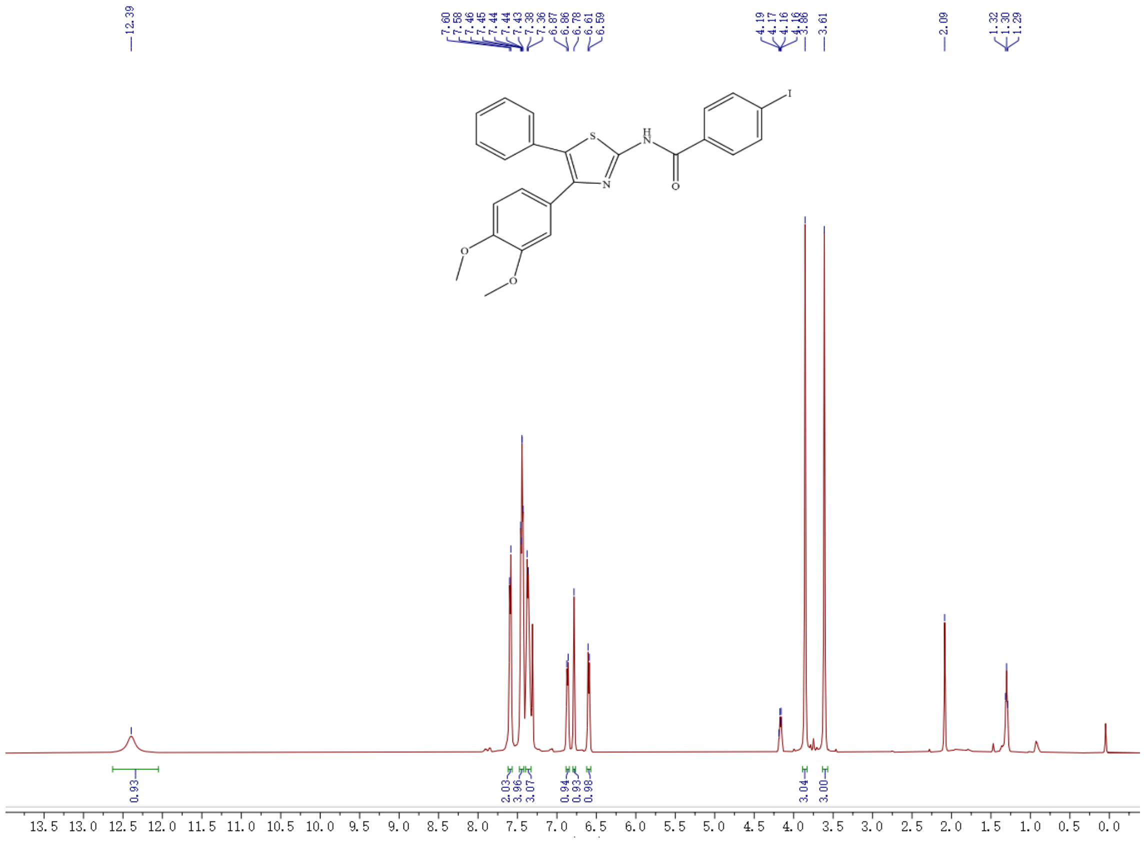
12.39
7.60 7.58 7.46 7.45 7.44 7.44 7.43 7.38 7.36 6.87 6.86 6.78 6.61 6.59
4.19 4.17 4.16 4.16 3.86
3.61
2.09
1.32 1.30 1.29
S
N
H
O
I
0.93
2.03 3.96 3.07
0.94 0.93 0.98
3.04 3.00
13.5 13.0 12.5 12.0 11.5 11.0 10.5 10.0 9.5 9.0 8.5 8.0 7.5 7.0 6.5 6.0 5.5 5.0 4.5 4.0 3.5 3.0 2.5 2.0 1.5 1.0 0.5 0.0

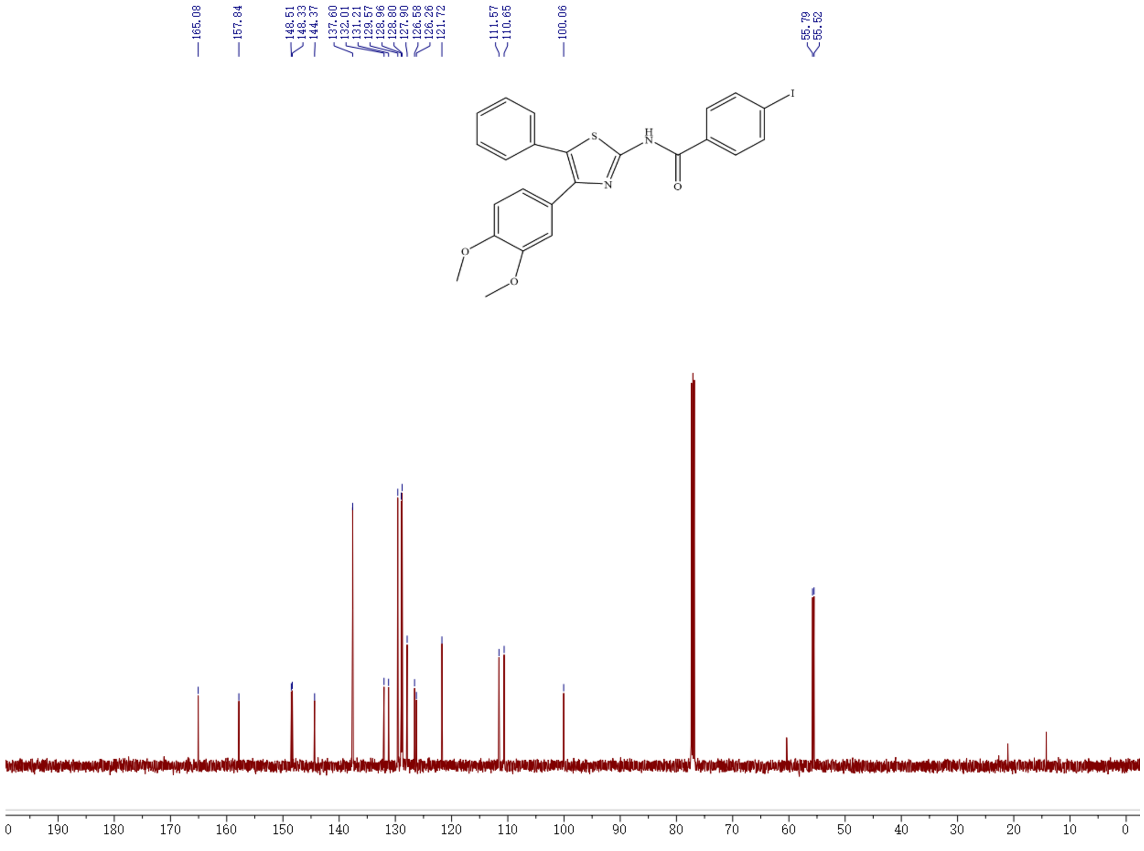
165.08
157.84
148.51 148.33 144.37 137.60 132.01 131.21 129.57 128.96 128.80 127.90 126.58 126.26 121.72
111.57 110.65
100.06
55.79 55.52
S
N
H
O
I
0 190 180 170 160 150 140 130 120 110 100 90 80 70 60 50 40 30 20 10 0

Fig.S30. 1H NMR And 13C NMR Spectrum of compound 4a15

_N_-[4-(3, 4-dimethoxyphenyl)-5-phenyl-1, 3-thiazol-2-yl]-4-trifluoromethylbenzamide (4a16)

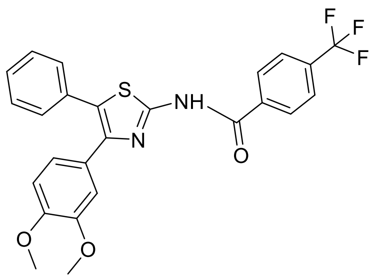

4a16, yield, 64%, white solid, m.p. 180.5-181.6°C. **^{1}H NMR** (500 MHz, $CDCl_3$) δ 12.67 (s, 1H), 7.82 (d, J = 8.1 Hz, 2H), 7.47 (d, J = 8.2 Hz, 2H), 7.41 – 7.35 (m, 2H), 7.35 – 7.26 (m, 3H), 6.76 (dd, J = 8.2, 2.0 Hz, 1H), 6.74 (d, J = 2.0 Hz, 1H), 6.45 (d, J = 8.3 Hz, 1H), 3.75 (s, 3H), 3.54 (s, 3H). **^{13}C NMR** (125 MHz, $CDCl_3$) δ 164.5, 157.9, 148.4 (d, J = 27.2 Hz), 144.3, 134.9, 133.8, 133.5, 131.8, 129.5, 128.9, 128.1 (d, J = 3.6 Hz), 126.4 (d, J = 21.3 Hz), 125.27 (q, J = 2.8 Hz), 124.58, 122.41, 121.63, 111.35, 110.68, 55.58, 55.35. HRMS, cald. ($C_{25}H_{19}F_3N_2O_3S$+H) = 485.1147, Found, 485.1143.

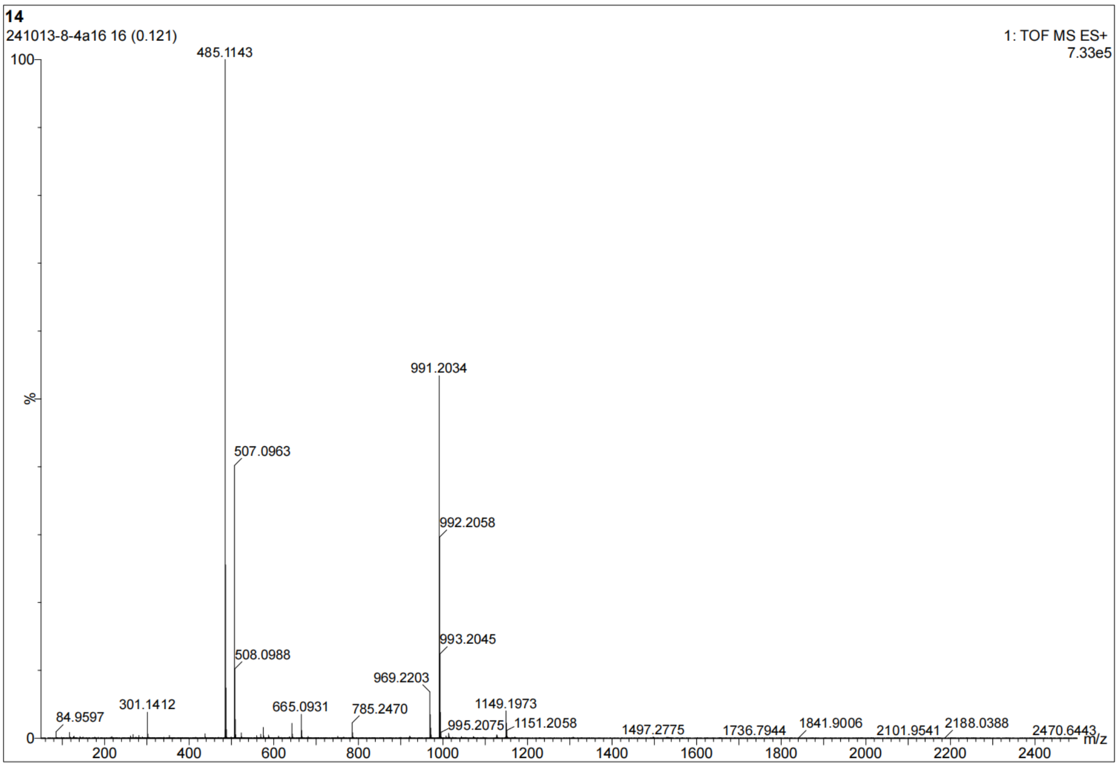

Fig.S31. Mass spectrum of compound 4a16

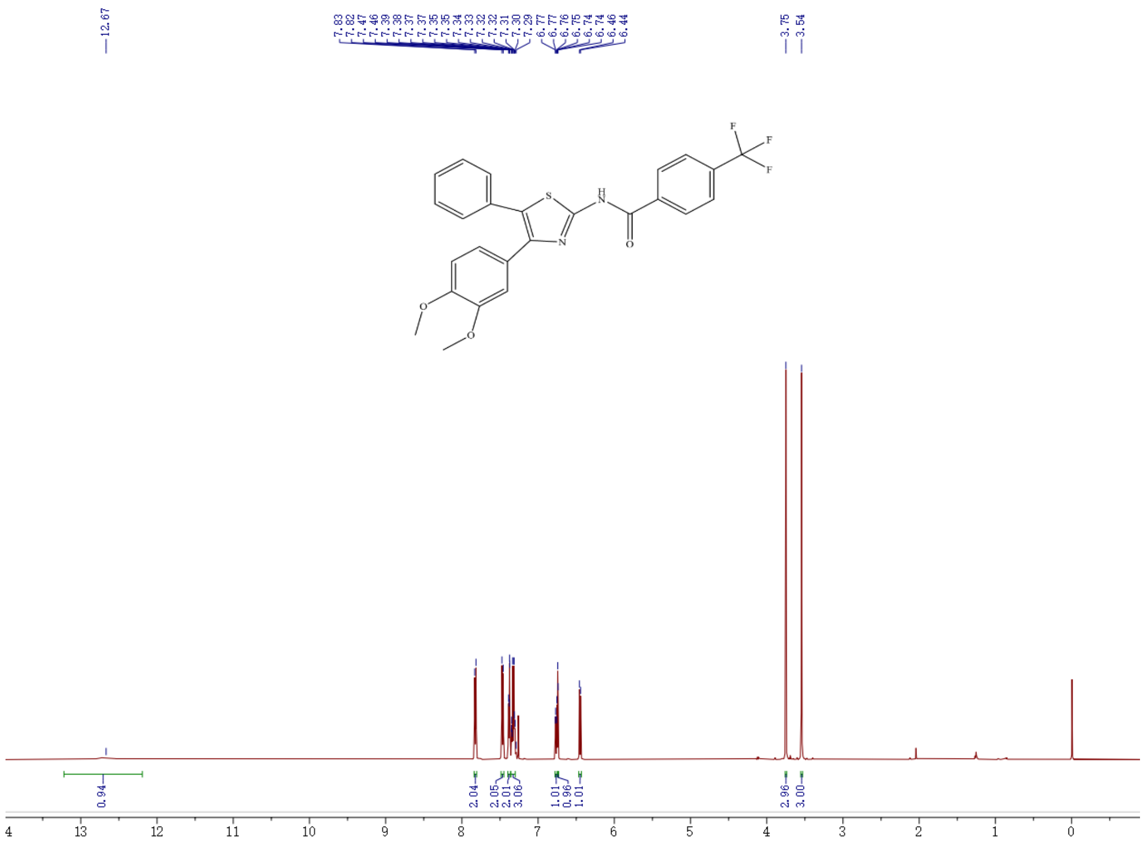

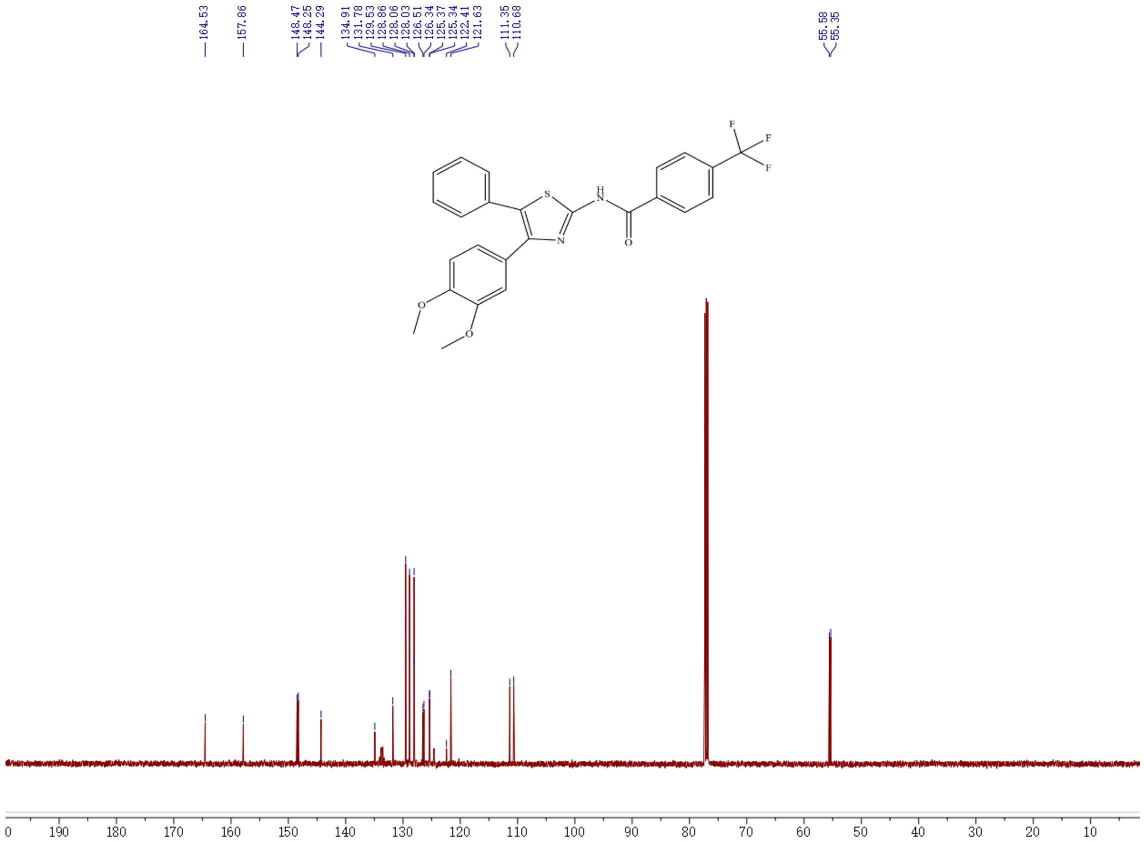

Fig.S32. 1H NMR And 13C NMR Spectrum of compound 4a16

***N*-[4-(3, 4-Dimethoxyphenyl)-5-phenyl-1, 3-thiazol-2-yl]thiophene-2-carboxamide (4a17)**

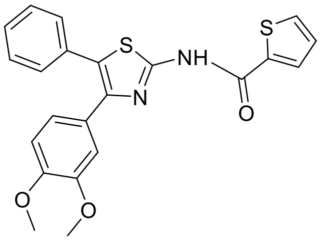

4a17, yield, 68%, white solid, m.p. 158.4-161.9℃. **^{1}H NMR** (500 MHz, $CDCl_3$) δ 11.51 (s, 1H), 7.46 (dd, *J* = 4.9, 1.1 Hz, 1H), 7.42 – 7.38 (m, 3H), 7.34 (d, *J* = 1.9 Hz, 1H), 7.33 (s, 1H), 7.32 – 7.28 (m, 1H), 6.99 (dd, *J* = 8.3, 2.0 Hz, 1H), 6.85 (dd, *J* = 5.0, 3.8 Hz, 1H), 6.81 (d, *J* = 2.0 Hz, 1H), 6.62 (d, *J* = 8.3 Hz, 1H), 3.79 (s, 3H), 3.56 (s, 3H).**^{13}C NMR** (125 MHz, $CDCl_3$) δ 160.0, 157.3, 148.6, 148.3, 144.2, 136.5, 132.2, 132.1, 130.0, 129.6, 128.8, 127.9, 127.9, 126.9, 126.2, 121.5, 111.9, 110.9, 55.8, 55.5. HRMS, cald. ($C_{22}H_{18}N_2O_3S_2$+H) = 423.0837, Found, 423.0839.

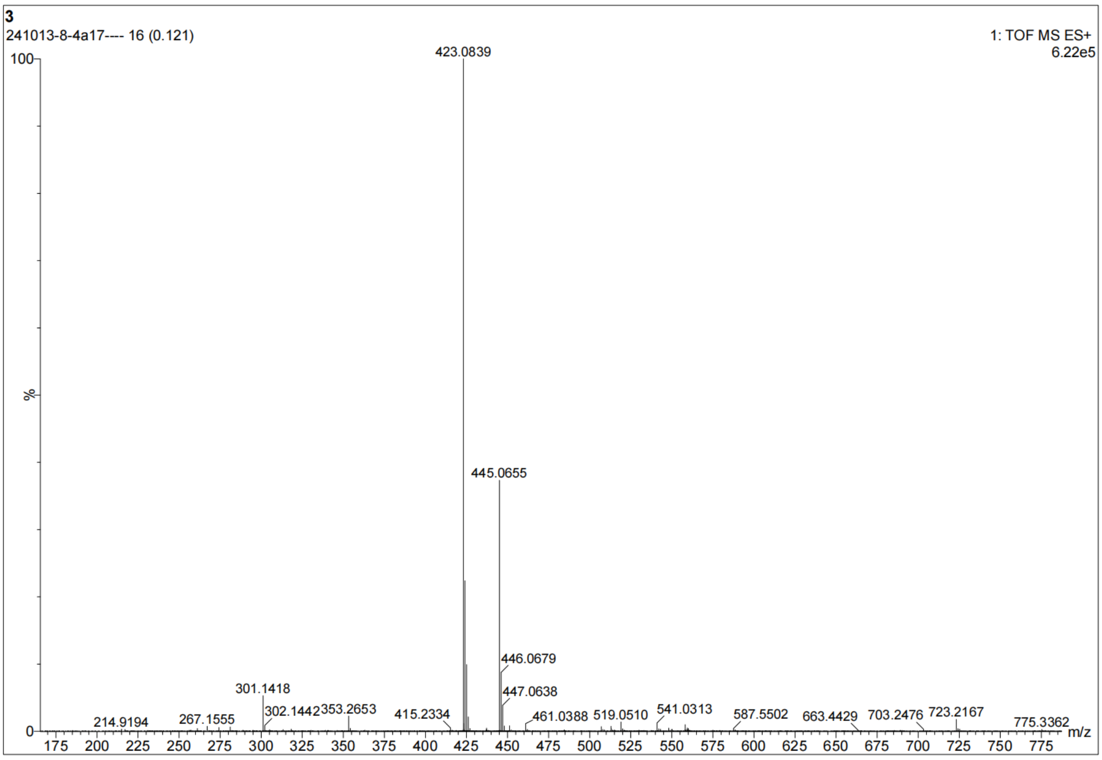

Fig.S33. Mass spectrum of compound 4a17

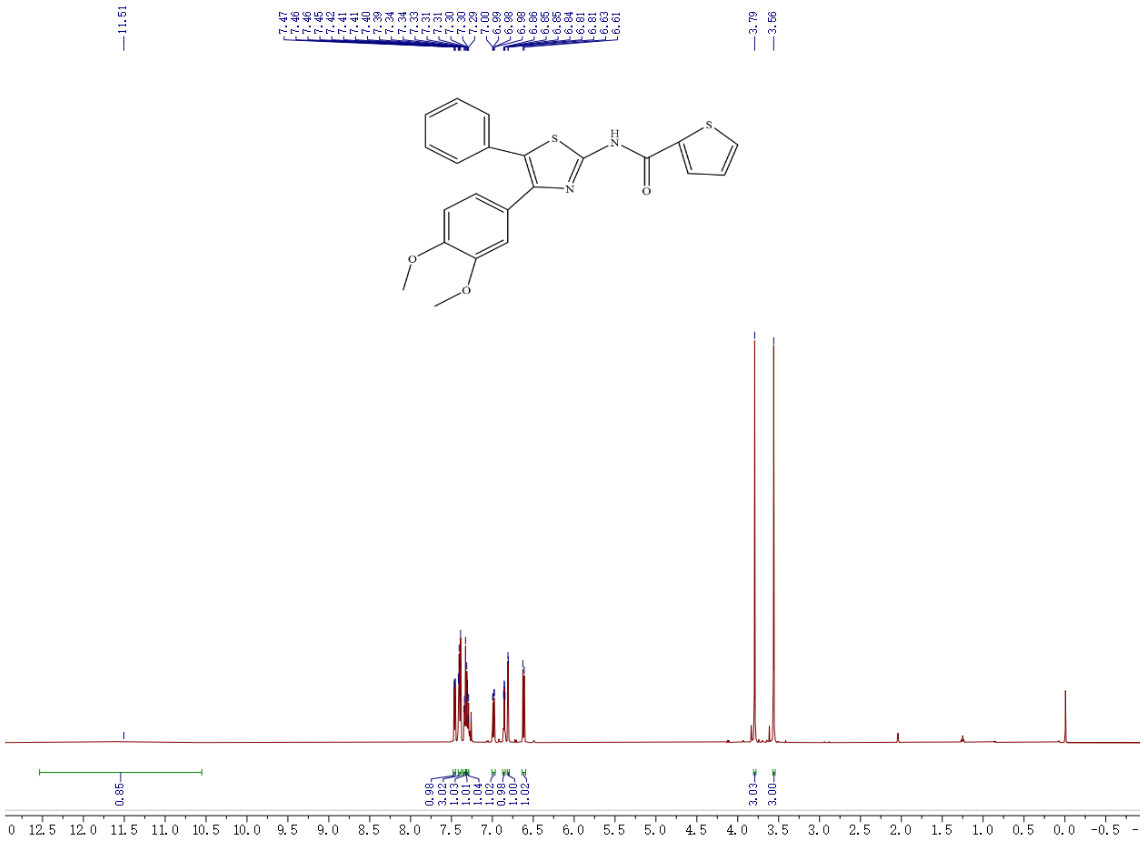

11.51
7.47
7.46
7.46
7.45
7.42
7.41
7.41
7.40
7.39
7.34
7.34
7.33
7.31
7.31
7.30
7.30
7.29
7.00
6.99
6.98
6.98
6.86
6.85
6.85
6.84
6.81
6.81
6.63
6.61
3.79
3.56
0.85
0.98
3.02
1.03
1.01
1.04
1.02
0.98
1.00
1.02
3.03
3.00
12.5
12.0
11.5
11.0
10.5
10.0
9.5
9.0
8.5
8.0
7.5
7.0
6.5
6.0
5.5
5.0
4.5
4.0
3.5
3.0
2.5
2.0
1.5
1.0
0.5
0.0
-0.5

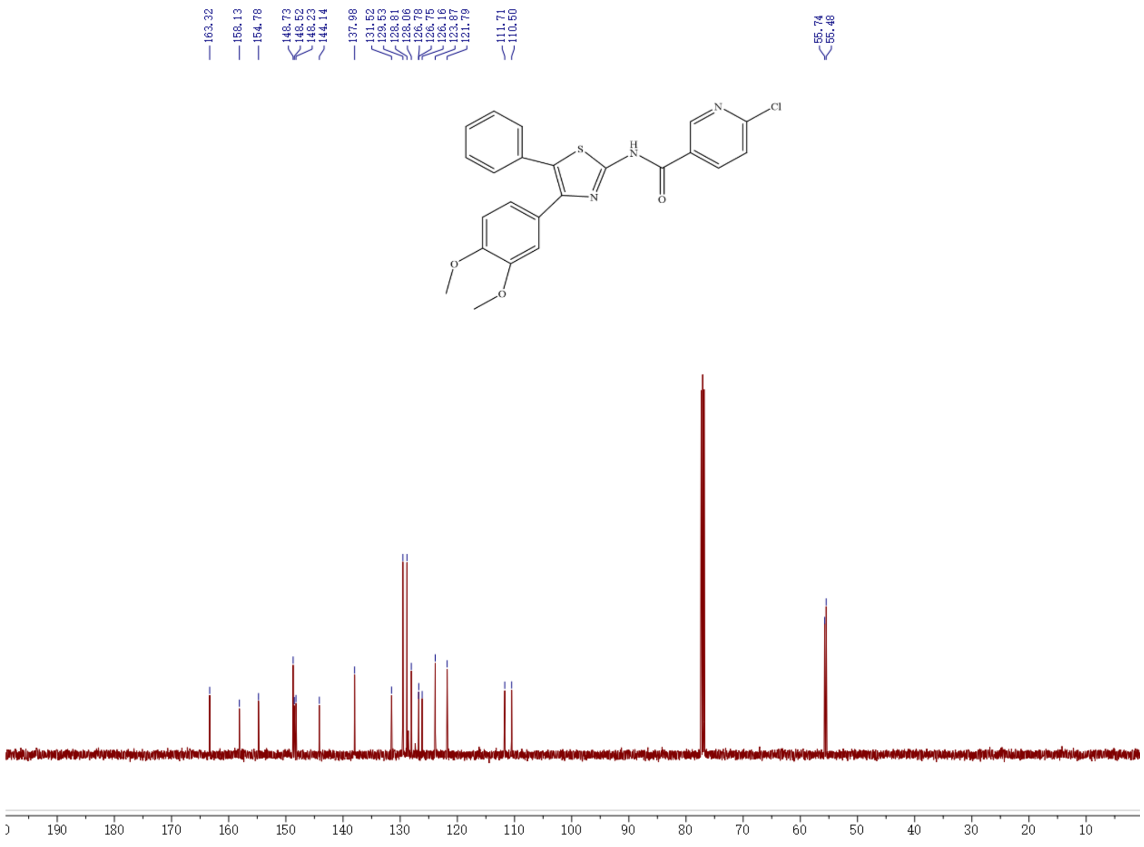

Fig.S34. 1H NMR And 13C NMR Spectrum of compound 4a17

6-Chloro-*N*-[4-(3, 4-dimethoxyphenyl)-5-phenyl-1, 3-thiazol-2-yl] pyridine-3-carboxamide (4a18)

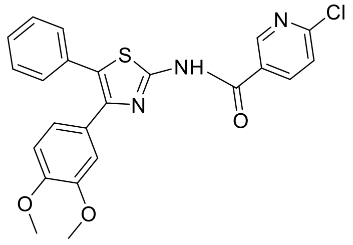

4a18, yield, 66%, yellowish powder, m.p. 220.6-221.5℃. **^{1}H NMR** (500 MHz, $CDCl_3$) δ 13.33 (s, 1H), 8.61 (d, J = 2.4 Hz, 1H), 7.91 (dd, J = 8.3, 2.5 Hz, 1H), 7.37 (d, J = 2.0 Hz, 1H), 7.35 (d, J = 1.5 Hz, 1H), 7.34 – 7.28 (m, 3H), 7.12 (d, J = 8.2 Hz, 1H), 6.80 (dd, J = 8.3, 2.0 Hz, 1H), 6.67 (d, J = 2.0 Hz, 1H), 6.51 (d, J = 8.3 Hz, 1H), 3.79 (s, 3H), 3.54 (s, 3H).**^{13}C NMR** (125 MHz, $CDCl_3$) δ 163.3, 158.1, 154.8, 148.7, 148.5, 148.2, 144.1, 138.0, 131.5, 129.5, 128.8, 128.1, 126.8, 126.8, 126.2, 123.9, 121.8, 111.7, 110.5, 55.7, 55.5. HRMS, cald. ($C_{23}H_{18}ClN_3O_3S$+H) = 452.0836, Found, 452.0836.

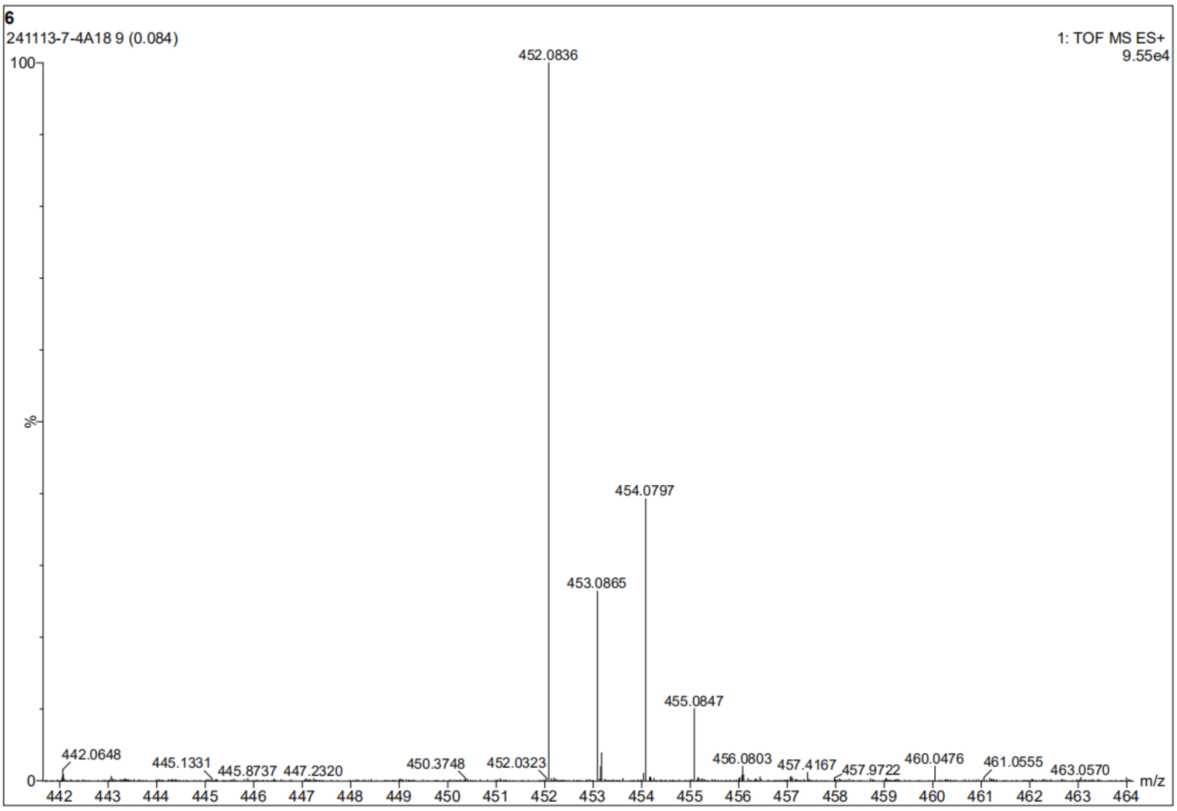

Fig.S35. Mass spectrum of compound 4a18

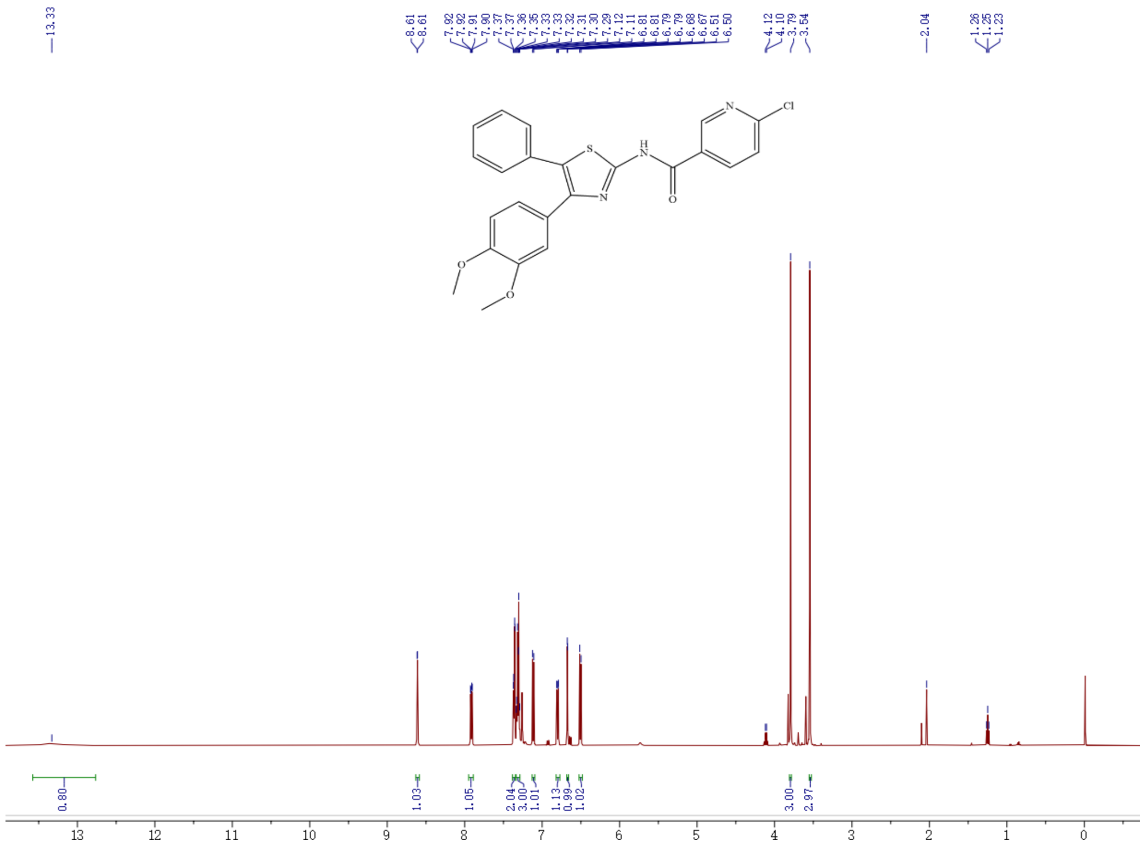
13.33
8.61 8.61
7.92 7.92 7.91 7.90 7.37 7.37 7.36 7.35 7.33 7.33 7.32 7.31 7.30 7.29 7.12 7.11 6.81 6.81 6.79 6.79 6.68 6.67 6.51 6.50
4.12 4.10 3.79 3.54
2.04
1.26 1.25 1.23
S
N
H
O
Cl
0.80
1.03
1.05
2.04 3.00 1.01
1.13 0.99 1.02
3.00 2.97
13 12 11 10 9 8 7 6 5 4 3 2 1 0

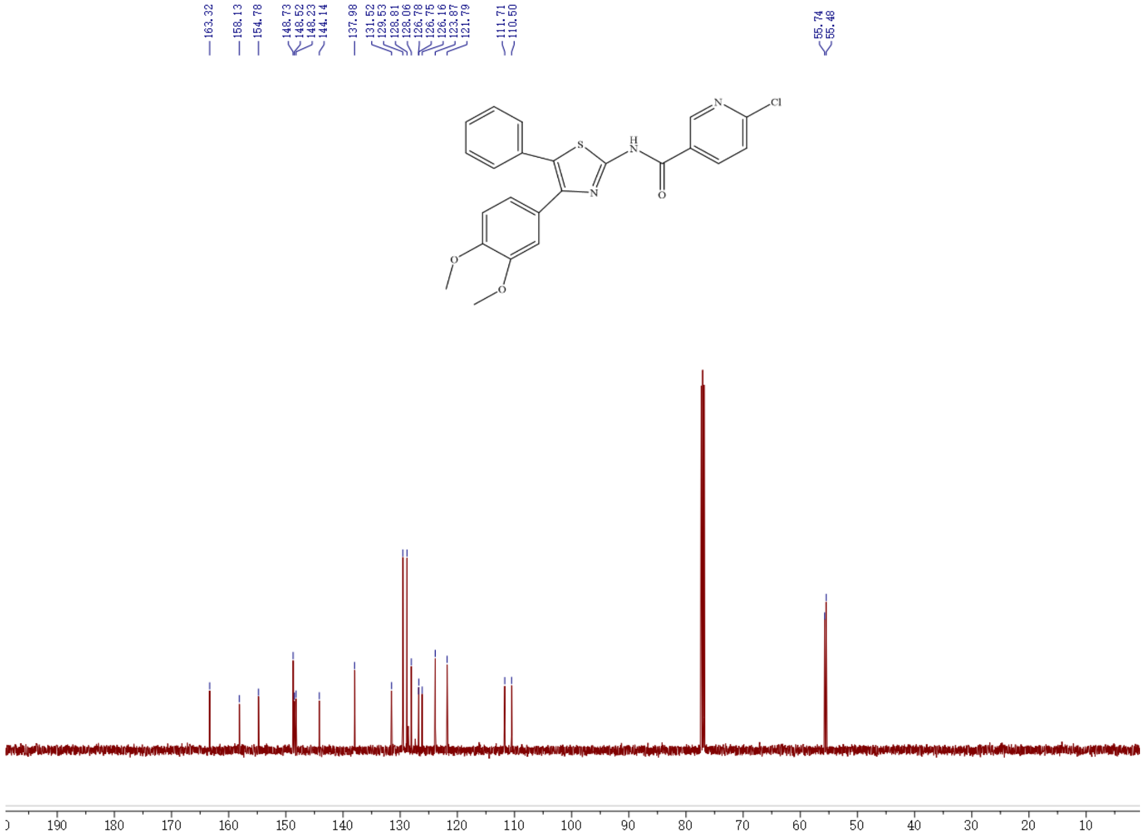
163.32
158.13
154.78
148.73 148.52 148.23 144.14
137.98
131.52 129.53 128.81 128.06 126.78 126.75 126.16 123.87 121.79
111.71 110.50
55.74 55.48
S
N
H
O
Cl
190 180 170 160 150 140 130 120 110 100 90 80 70 60 50 40 30 20 10

Fig.S36. 1H NMR And 13C NMR Spectrum of compound 4a18

2-Methoxy-*N*-[4-(4-methoxyphenyl)-5-phenyl-1, 3-thiazol-2-yl]benzamide (4b19)

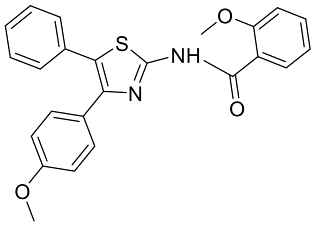

4b19, yield, 68%, white solid, m.p. 132.6-135.6℃. **^{1}H NMR** (500 MHz, $CDCl_3$) δ 11.13 (s, 1H), 8.37 (d, *J* = 7.5 Hz, 1H), 7.59 (t, *J* = 7.4 Hz, 1H), 7.52 (d, *J* = 8.4 Hz, 2H), 7.44 (d, *J* = 6.9 Hz, 2H), 7.35 (d, *J* = 7.5 Hz, 3H), 7.19 (t, *J* = 7.4 Hz, 1H), 7.10 (d, *J* = 8.3 Hz, 1H), 6.88 (d, *J* = 8.4 Hz, 2H), 4.13 (s, 3H), 3.85 (s, 3H).**^{13}C NMR** (125 MHz, $CDCl_3$) δ 162.7, 159.2, 157.8, 155.6, 144.7, 134.5, 132.7, 132.6, 130.2, 129.5, 128.7, 127.8, 127.6, 126.1, 121.7, 119.2, 113.8, 111.7, 56.4, 55.3. HRMS, cald. ($C_{24}H_{20}N_2O_3S$+H) = 417.1273, Found, 417.1267.

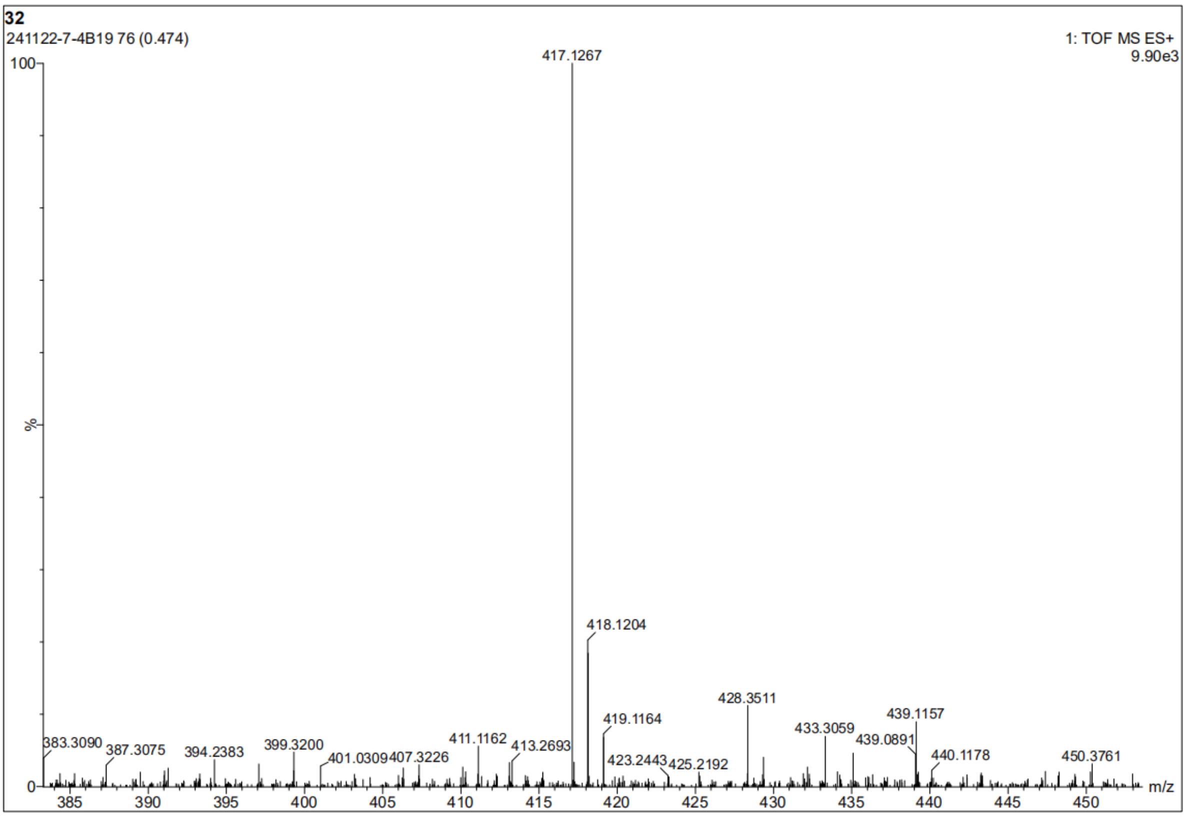

Fig.S37. Mass spectrum of compound 4b19

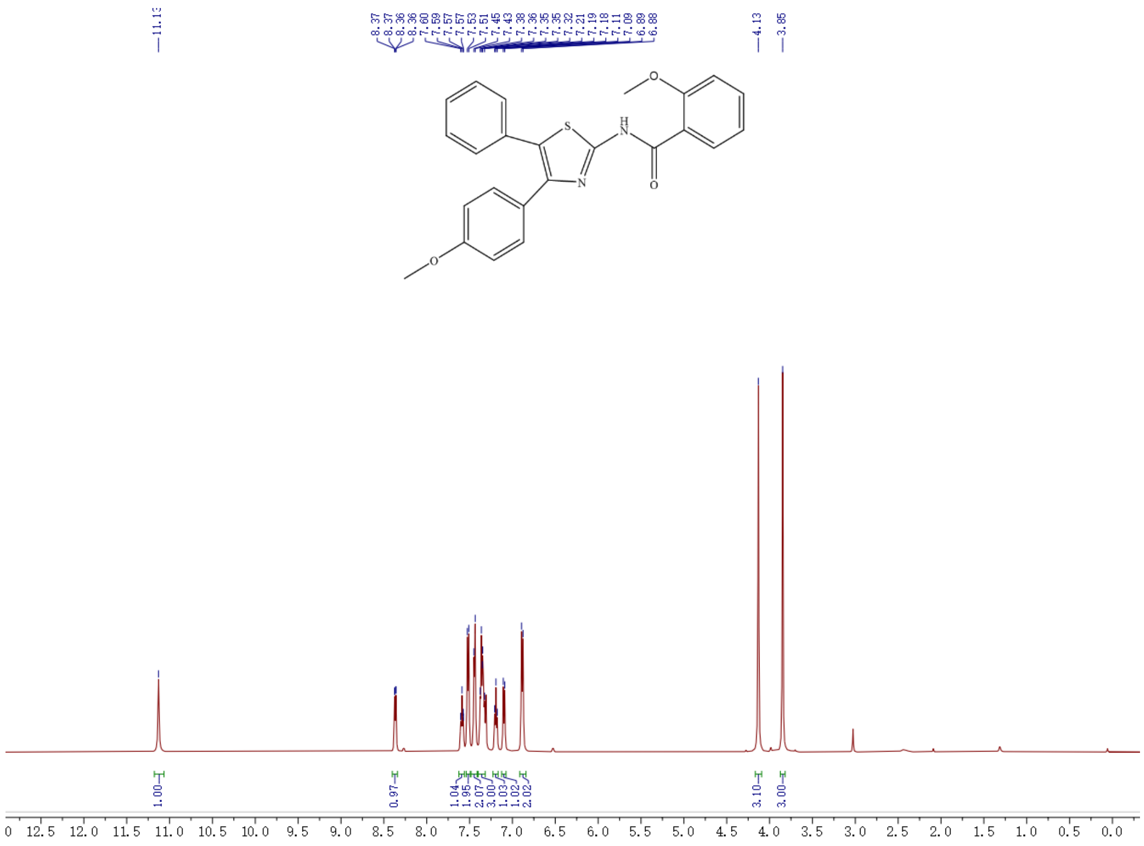

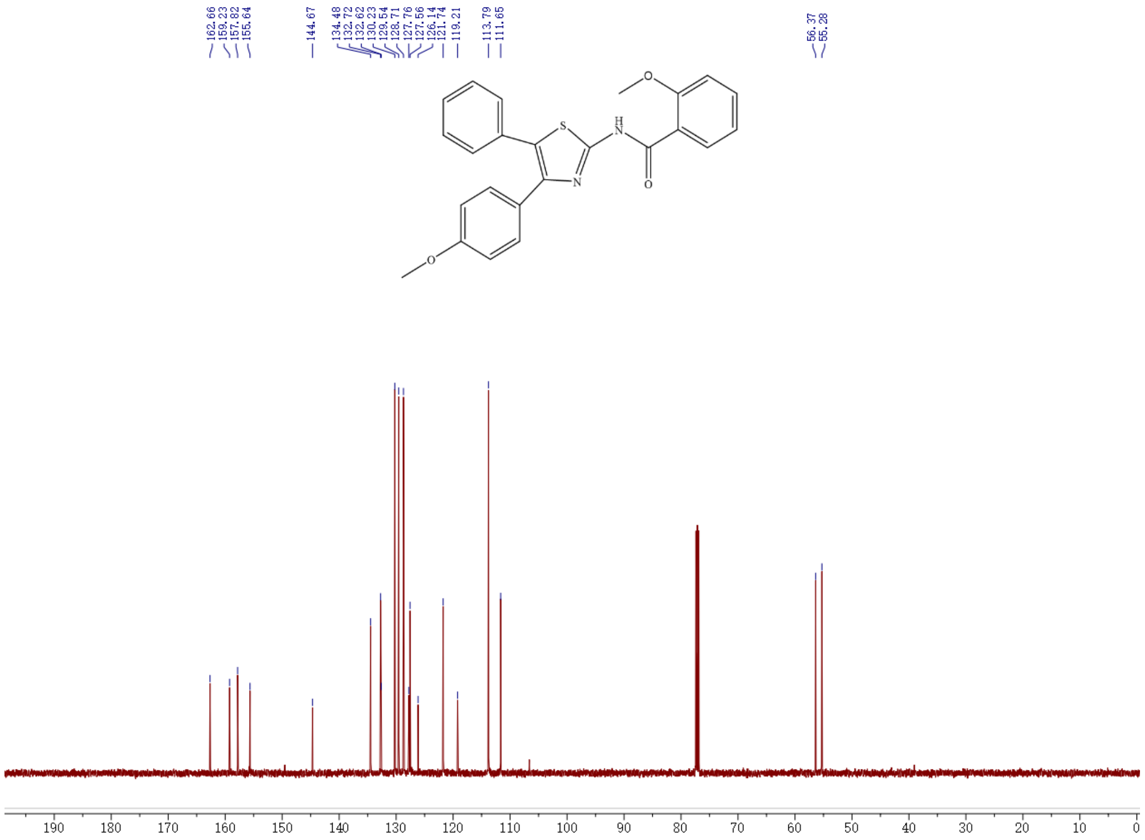

Fig.S38. 1H NMR And 13C NMR Spectrum of compound 4b19

***N*-[4-(4-Chlorophenyl)-5-phenyl-1, 3-thiazol-2-yl]-3-chlorobenzamide (4b20)**

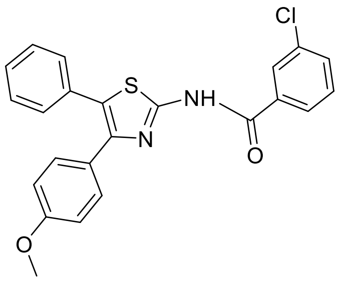

4b20, yield, 64%, white solid, m.p. 186.9-188.1°C. **^{1}H NMR** (500 MHz, $CDCl_3$) δ 12.67 (s, 1H), 7.67 (s, 1H), 7.56 (d, J = 7.8 Hz, 1H), 7.44 (dd, J = 7.8, 1.4 Hz, 2H), 7.37 (t, J = 7.8 Hz, 4H), 7.24 (d, J = 8.7 Hz, 2H), 7.17 (t, J = 7.9 Hz, 1H), 6.60 (d, J = 8.7 Hz, 2H), 3.76 (s, 3H).**^{13}C NMR** (125 MHz, $CDCl_3$) δ 164.5, 159.1, 157.8, 144.2, 134.6, 133.8, 132.4, 132.1, 129.9, 129.7, 129.5, 128.8, 128.0, 127.9, 126.5, 126.1, 125.6, 113.6, 55.2.HRMS, cald. ($C_{23}H_{17}ClN_2O_2S$+H) = 421.0778, Found, 421.0782.

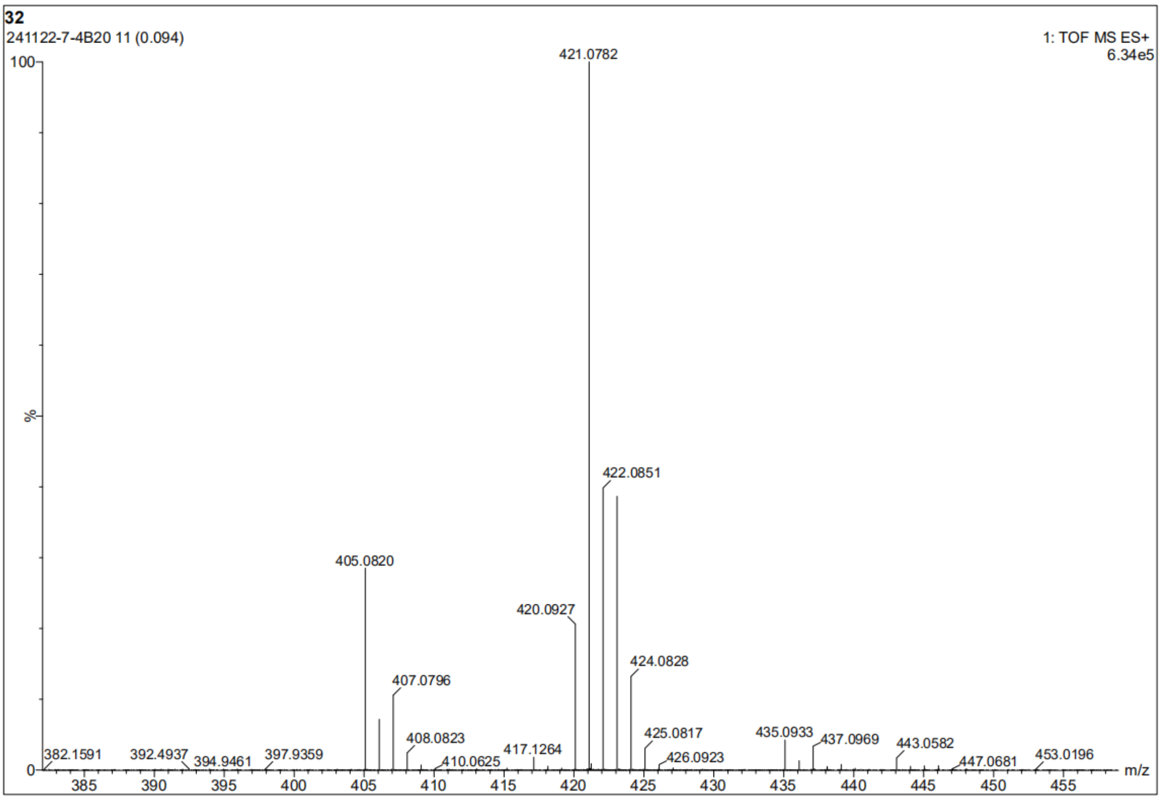

Fig.S39. Mass spectrum of compound 4b20

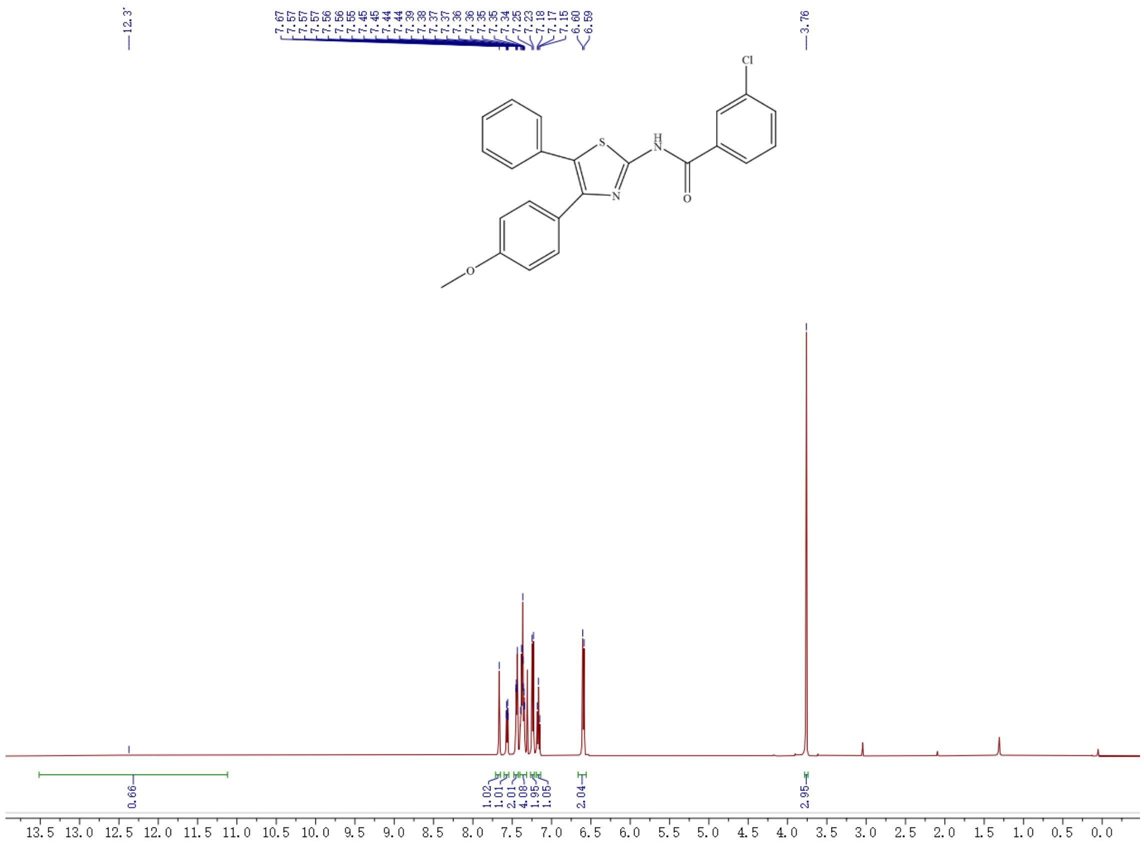
12.3[illegible]
7.67
7.57
7.57
7.57
7.56
7.55
7.45
7.45
7.44
7.44
7.39
7.38
7.37
7.37
7.36
7.36
7.35
7.35
7.34
7.25
7.23
7.18
7.17
7.15
6.60
6.59
3.76
Cl
S
H
N
N
O
O
0.66
1.02
1.01
2.01
4.08
1.95
1.06
2.04
2.95
13.5 13.0 12.5 12.0 11.5 11.0 10.5 10.0 9.5 9.0 8.5 8.0 7.5 7.0 6.5 6.0 5.5 5.0 4.5 4.0 3.5 3.0 2.5 2.0 1.5 1.0 0.5 0.0

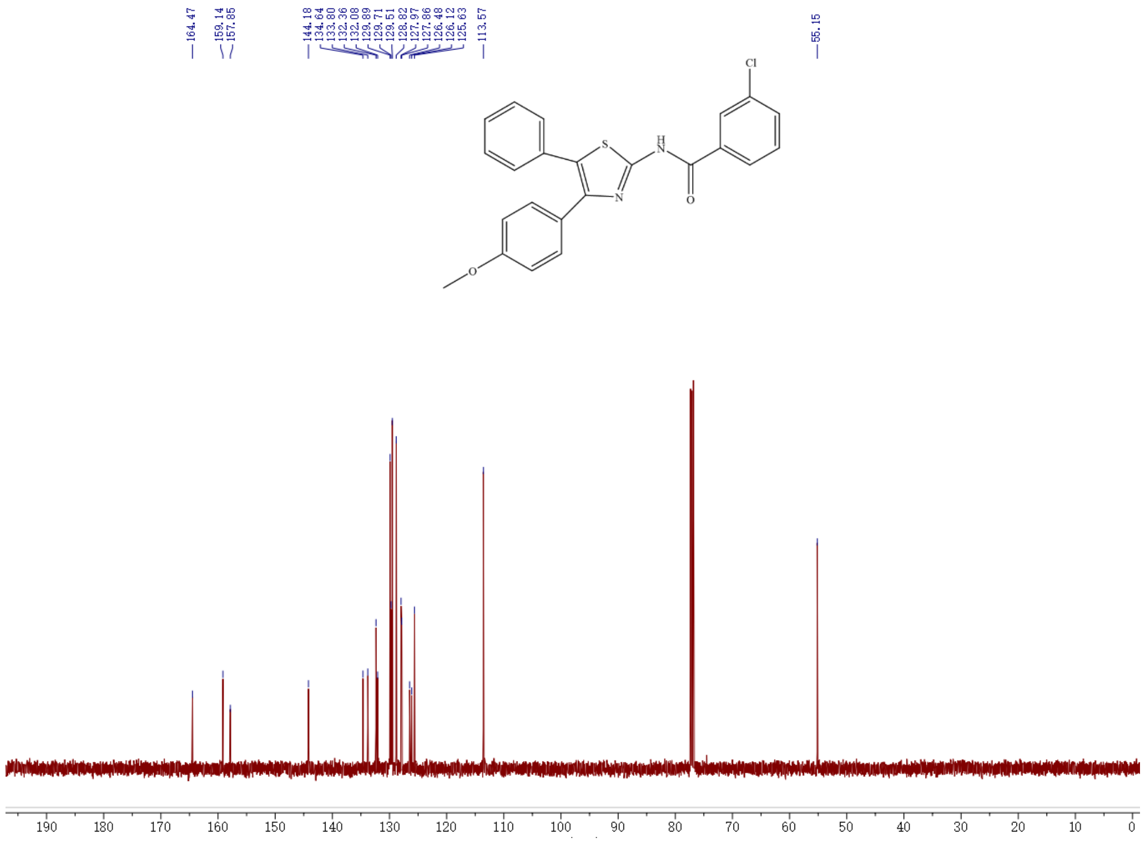

Fig.S40. 1H NMR And 13C NMR Spectrum of compound 4b20

_N_-[4-(4-Methoxyphenyl)-5-phenyl-1, 3-thiazol-2-yl]-4-chlorobenzamide (4b21)

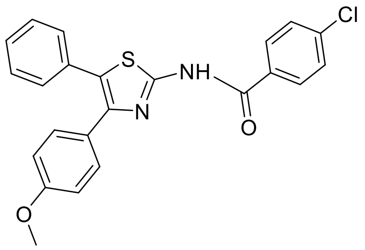

4b21, yield, 63%, white solid, m.p. 221.2-222.8℃. **^{1}H NMR** (500 MHz, $CDCl_3$) δ 12.36 (s, 1H), 7.63 (d, J = 8.1 Hz, 2H), 7.43 (d, J = 7.2 Hz, 2H), 7.37 (d, J = 7.7 Hz, 3H), 7.20 (dd, J = 13.2, 8.5 Hz, 4H), 6.61 (d, J = 8.3 Hz, 2H), 3.76 (s, 3H).**^{13}C NMR** (125 MHz, $CDCl_3$) δ 164.8, 159.1, 158.0, 144.3, 138.7, 132.1, 130.4, 130.1, 129.4, 129.0, 128.8, 128.7, 127.8, 126.5, 126.1, 113.6, 55.1. HRMS, cald. ($C_{23}H_{17}ClN_2O_2S$+H) = 421.0778, Found, 421.0773.

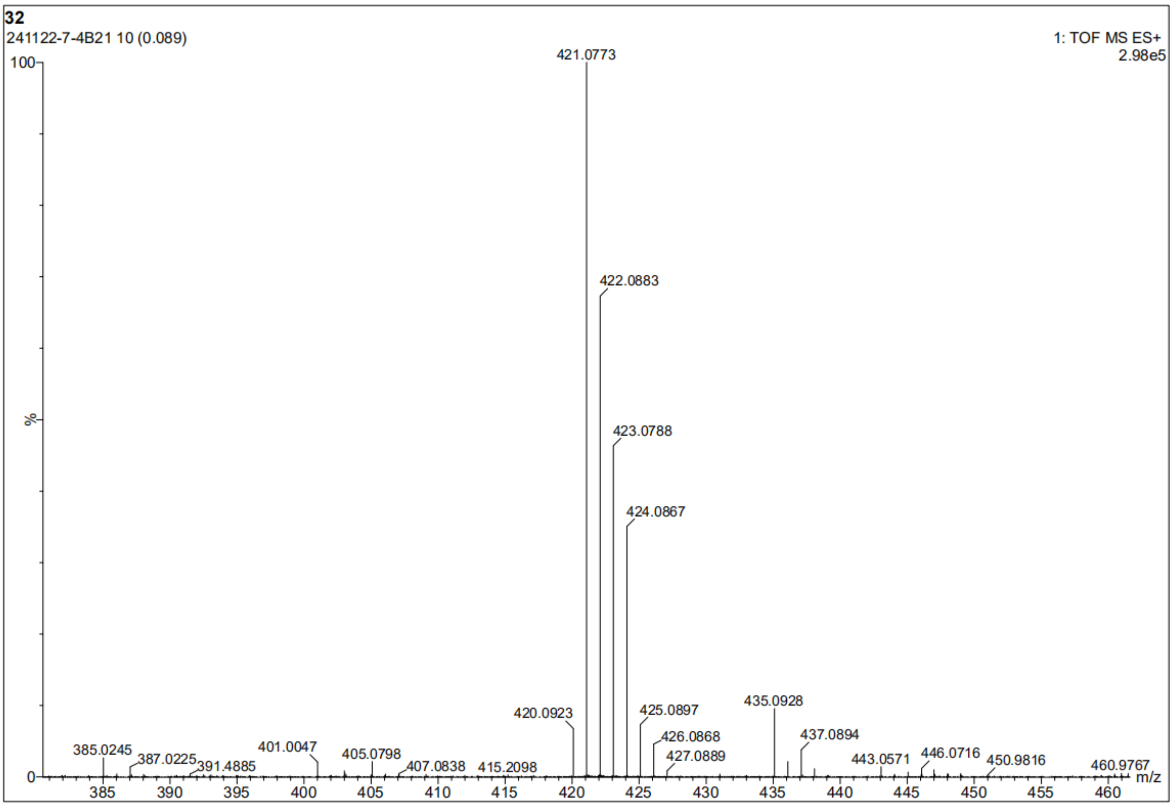

Fig.S41. Mass spectrum of compound 4b21

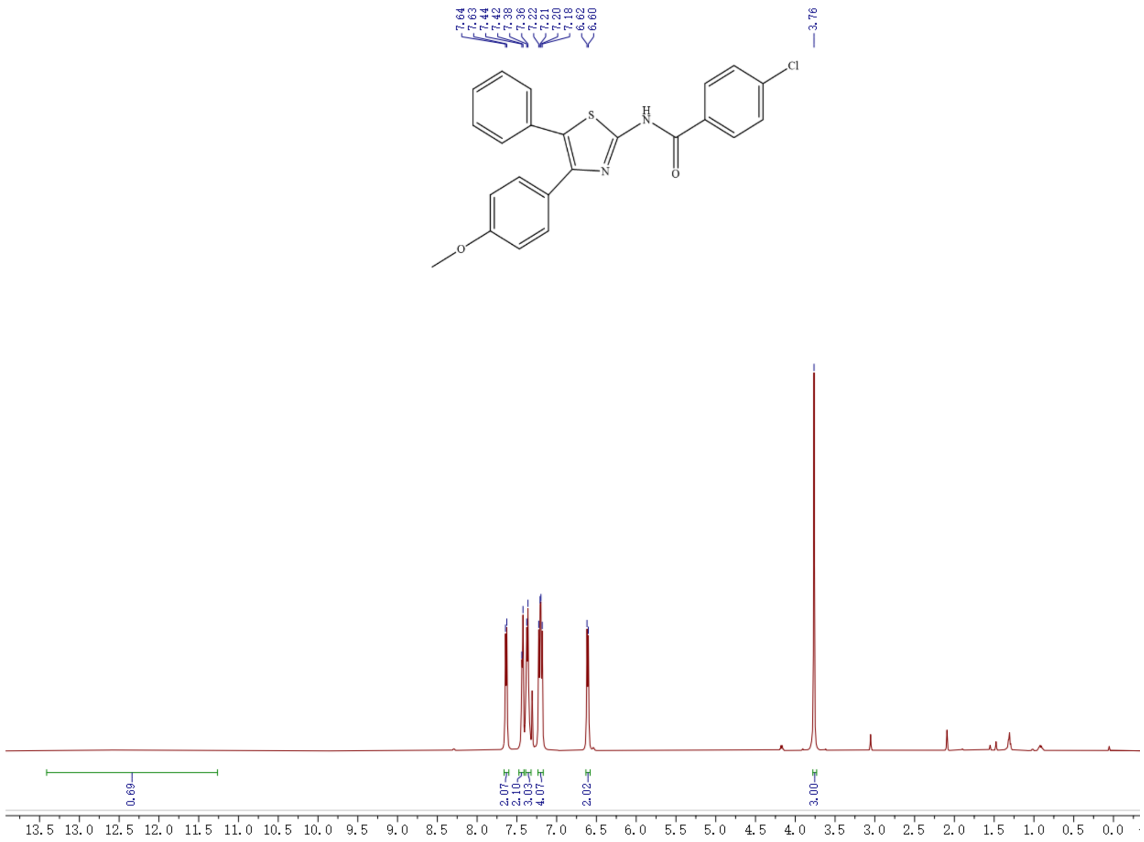
7.64
7.63
7.44
7.42
7.38
7.36
7.22
7.21
7.20
7.18
6.62
6.60
3.76
S
H
N
N
O
Cl
O
0.69
2.07
2.10
3.03
4.07
2.02
3.00
13.5 13.0 12.5 12.0 11.5 11.0 10.5 10.0 9.5 9.0 8.5 8.0 7.5 7.0 6.5 6.0 5.5 5.0 4.5 4.0 3.5 3.0 2.5 2.0 1.5 1.0 0.5 0.0

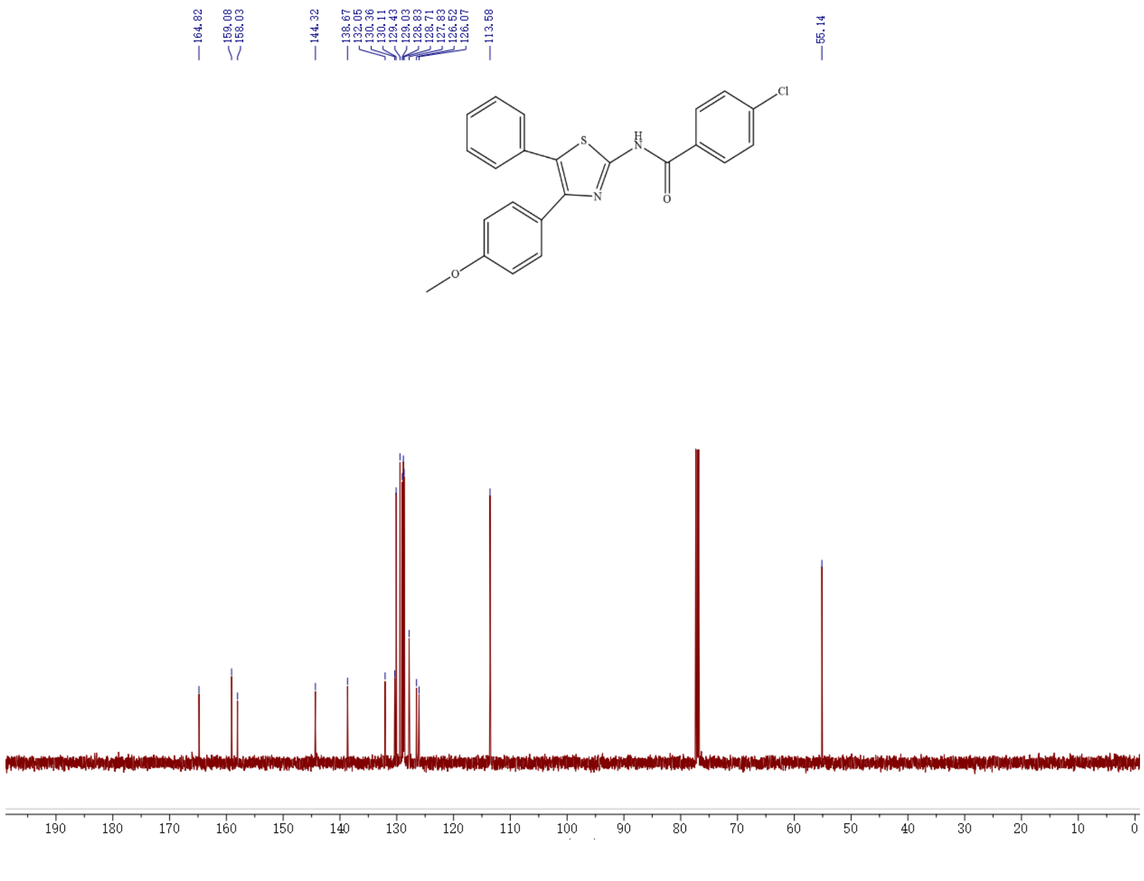
164.82
159.08
158.03
144.32
138.67
132.05
130.36
130.11
129.43
129.03
128.83
128.71
127.83
126.52
126.07
113.58
55.14
S
H
N
N
O
Cl
O
190 180 170 160 150 140 130 120 110 100 90 80 70 60 50 40 30 20 10 0

Fig.S42. 1H NMR And 13C NMR Spectrum of compound 4b21

6-Chloro-*N*-[4-(4-methoxyphenyl)-5-phenyl-1,3-thiazol-2-yl]pyridine-3-carboxamide (4b22)

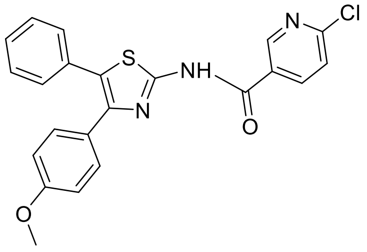

4b22, yield, 63%, yellowish solid, m.p. 180.0-183.4℃. **^{1}H NMR** (500 MHz, $CDCl_3$) δ 12.97 (s, 1H), 8.72 (d, *J* = 2.5 Hz, 1H), 7.94 (dd, *J* = 8.4, 2.5 Hz, 1H), 7.41 (dd, *J* = 4.6, 2.8 Hz, 2H), 7.39 – 7.34 (m, 3H), 7.23 (s, 1H), 7.21 (s, 1H), 7.18 (d, *J* = 8.4 Hz, 1H), 6.64 (s, 1H), 6.63 (s, 1H), 3.79 (s, 3H).**^{13}C NMR** (125 MHz, $CDCl_3$) δ 163.1, 159.3, 158.2, 157.8, 155.0, 149.0, 137.8, 131.7, 130.2, 129.4, 128.8, 128.0, 126.9, 126.7, 126.2, 124.1, 113.6, 55.2. HRMS, cald. ($C_{22}H_{16}ClN_3O_2S$+H) =422.0730, Found, 422.0729.

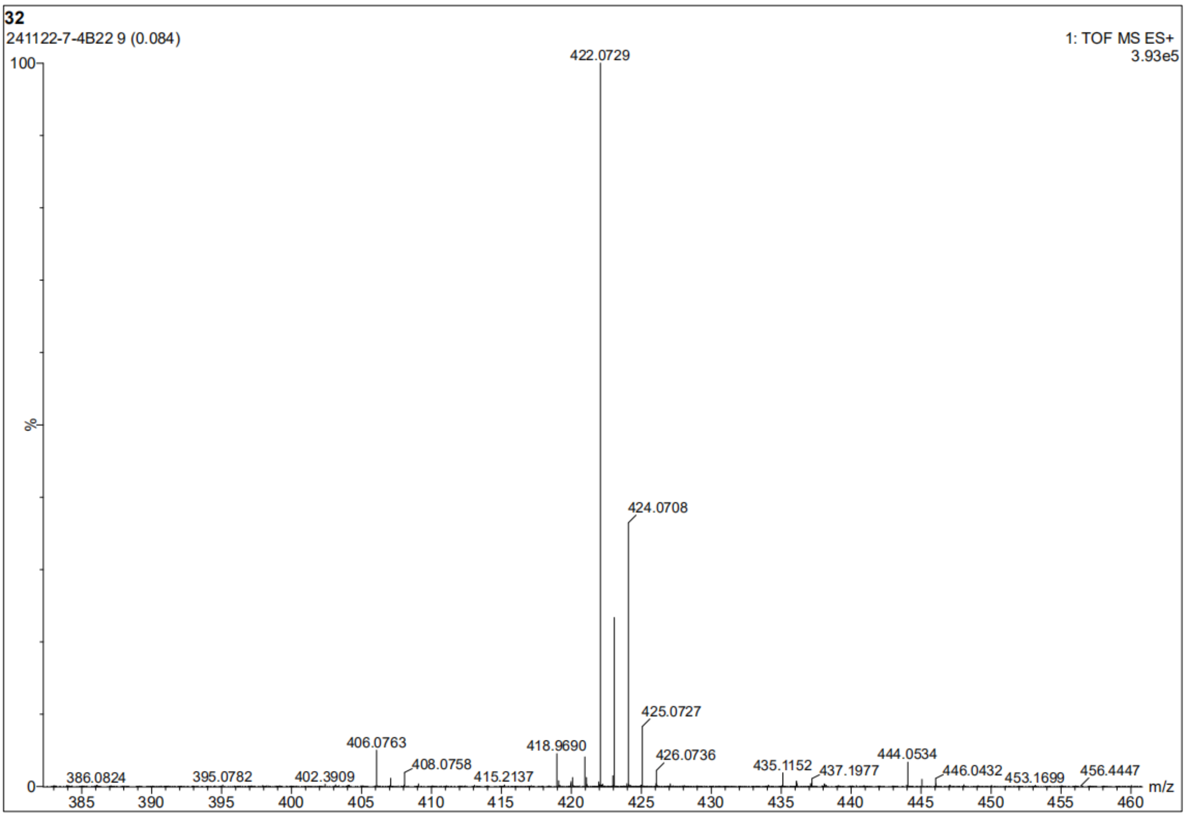

Fig.S43. Mass spectrum of compound 4b22

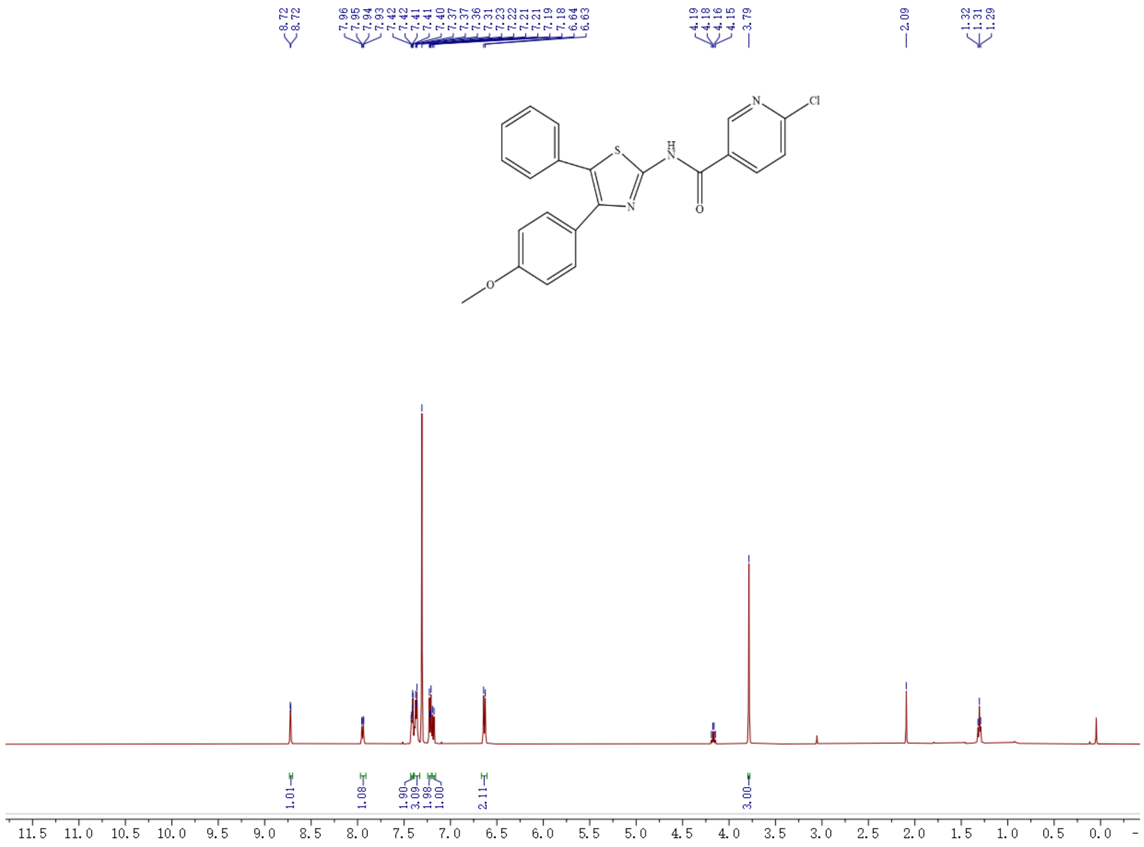
8.72
8.72
7.96
7.95
7.94
7.93
7.42
7.42
7.41
7.41
7.40
7.37
7.37
7.36
7.31
7.23
7.22
7.21
7.21
7.19
7.18
6.64
6.63
4.19
4.18
4.16
4.15
3.79
2.09
1.32
1.31
1.29
S
H
N
N
O
Cl
O
1.01
1.08
1.90
3.09
1.98
1.00
2.11
3.00
11.5 11.0 10.5 10.0 9.5 9.0 8.5 8.0 7.5 7.0 6.5 6.0 5.5 5.0 4.5 4.0 3.5 3.0 2.5 2.0 1.5 1.0 0.5 0.0

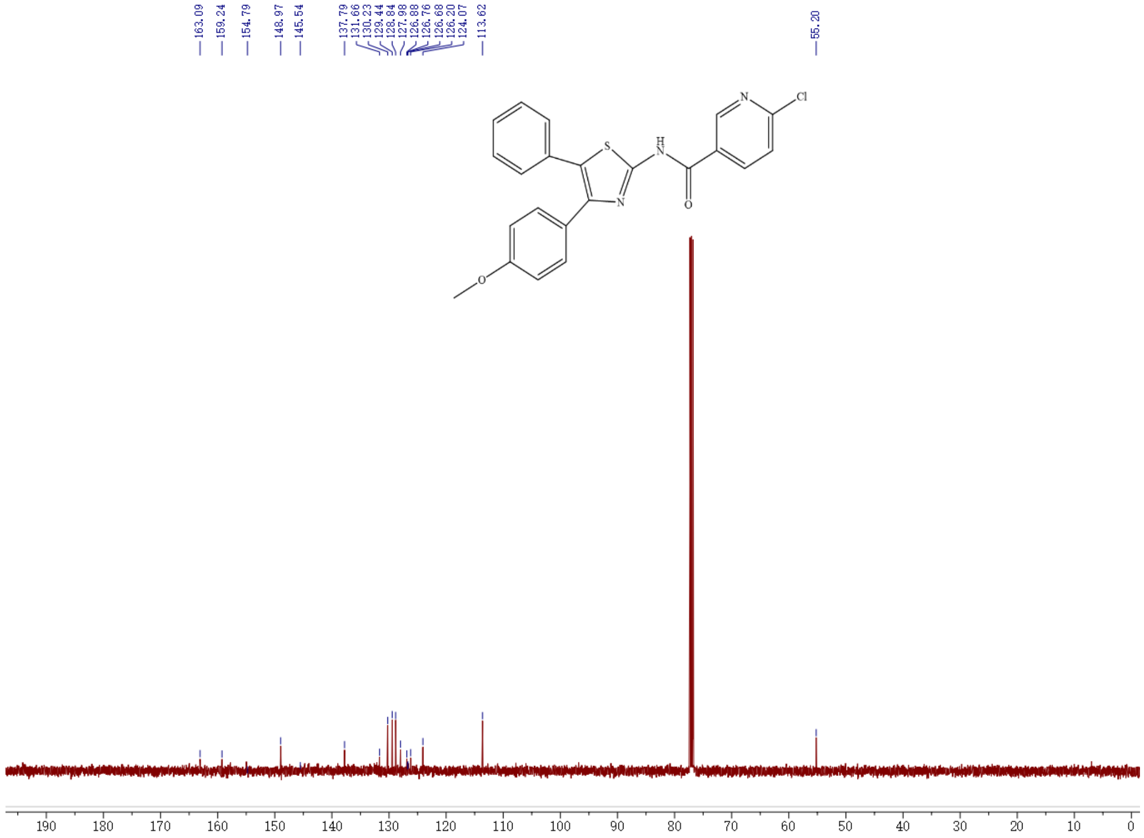
163.09
159.24
154.79
148.97
145.54
137.79
131.66
130.23
129.44
128.84
127.98
126.88
126.76
126.68
126.20
124.07
113.62
55.20
S
H
N
N
O
Cl
O
190 180 170 160 150 140 130 120 110 100 90 80 70 60 50 40 30 20 10 0

Fig.S44. 1H NMR And 13C NMR Spectrum of compound 4b22

N-(4-(4-Methoxyphenyl)-5-phenylthiazol-2-yl)-1-naphthamide (4b23)

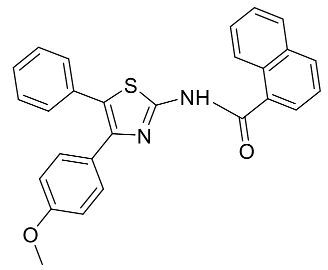

4b23, yield, 64%, white solid, m.p. 183.7-185.3℃. **^{1}H NMR** (500 MHz, $CDCl_3$) *δ* 12.35 (s, 1H), 8.43 (d, *J* = 9.1 Hz, 1H), 7.85 (d, *J* = 8.0 Hz, 2H), 7.63 – 7.54 (m, 3H), 7.42 – 7.31 (m, 6H), 7.04 (d, *J* = 8.8 Hz, 2H), 6.38 (d, *J* = 8.8 Hz, 2H), 3.61 (s, 3H). **^{13}C NMR** (125 MHz, $CDCl_3$) *δ* 167.1, 158.6, 157.5, 143.9, 133.8, 132.3, 132.3, 130.8, 130.2, 129.5, 129.4, 128.8, 128.3, 127.8, 127.2, 126.5, 126.5, 126.2, 125.4, 125.2, 124.3, 113.1, 54.8. HRMS, cald. ($C_{27}H_{20}N_2O_2S$+H) =437.1324, Found, 437.1328.

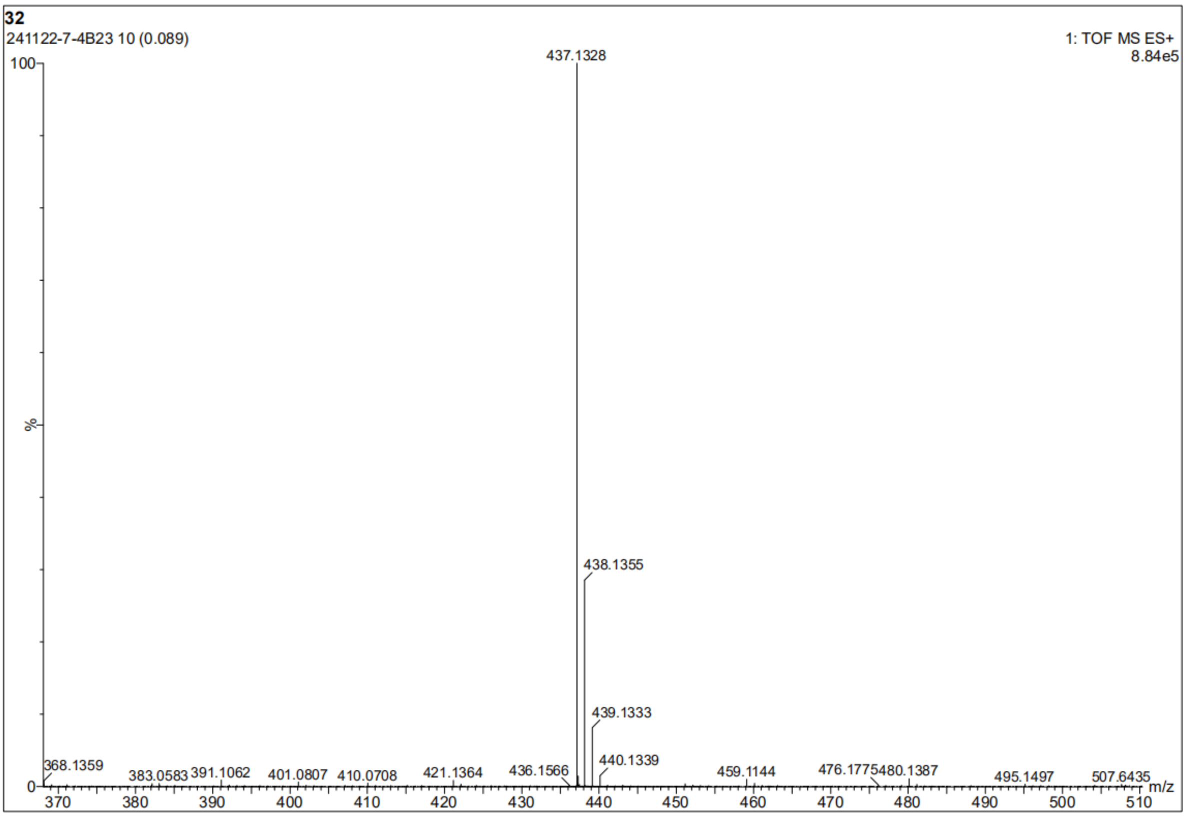

Fig.S45. Mass spectrum of compound 4b23

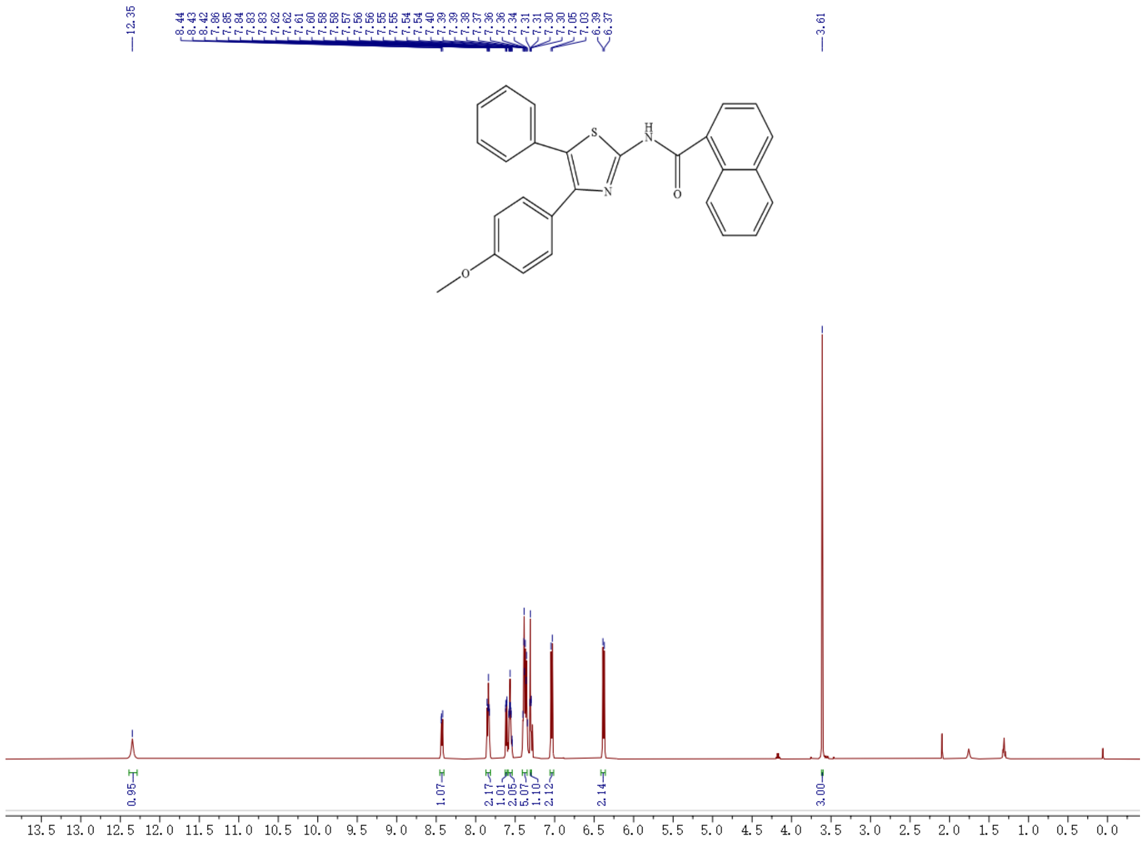

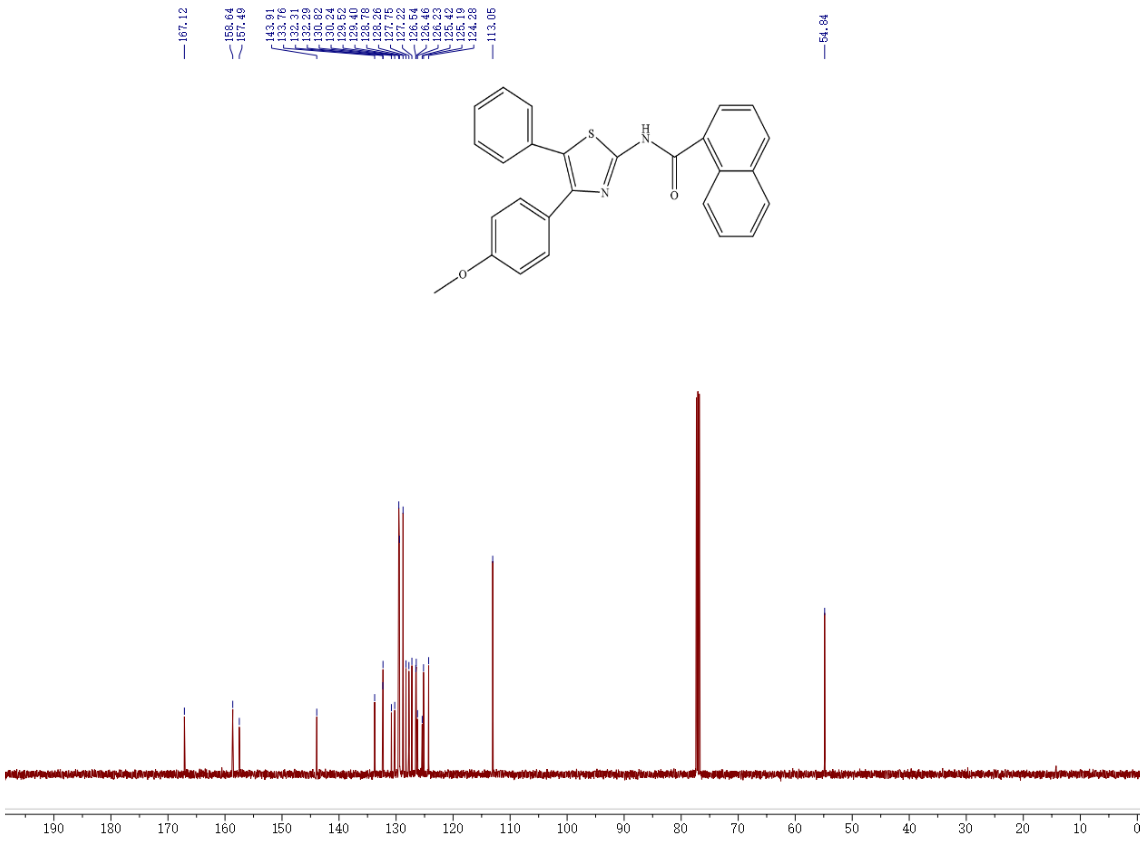

Fig.S46. 1H NMR And 13C NMR Spectrum of compound 4b23

N-(4,5-Bis(4-methoxyphenyl)thiazol-2-yl)-3-fluorobenzamide（4c24）

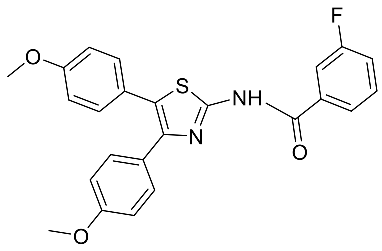

4c24, yield, 68%, white solid, m.p. 102.3-105.4℃. **^{1}H NMR** (500 MHz, $CDCl_3$) δ 12.15 (s, 1H), 7.47 (d, J = 7.8 Hz, 1H), 7.41 (d, J = 9.0 Hz, 1H), 7.36 – 7.30 (m, 3H), 7.24 (s, 2H), 7.09 (d, J = 8.2 Hz, 1H), 6.91 (d, J = 8.7 Hz, 2H), 6.61 (s, 2H), 3.87 (s, 3H), 3.76 (s, 3H).**^{13}C NMR** (125 MHz, $CDCl_3$) δ 164.4, 163.4, 161.5, 159.2 (d, J = 42.7 Hz), 157.2, 143.6, 134.3(d, J = 7.0 Hz), 130.7, 130.1 (d, J = 7.8 Hz), 129.9, 126.7, 126.0, 124.2, 123.1 (d, J = 3.0 Hz), 119.4 (d, J = 21.4 Hz), 114.9 (d, J = 23.4 Hz), 114.3, 113.6, 55.3, 55.1. HRMS, cald. ($C_{24}H_{19}FN_2O_3S$+H) = 435.1179, Found, 435.1182.

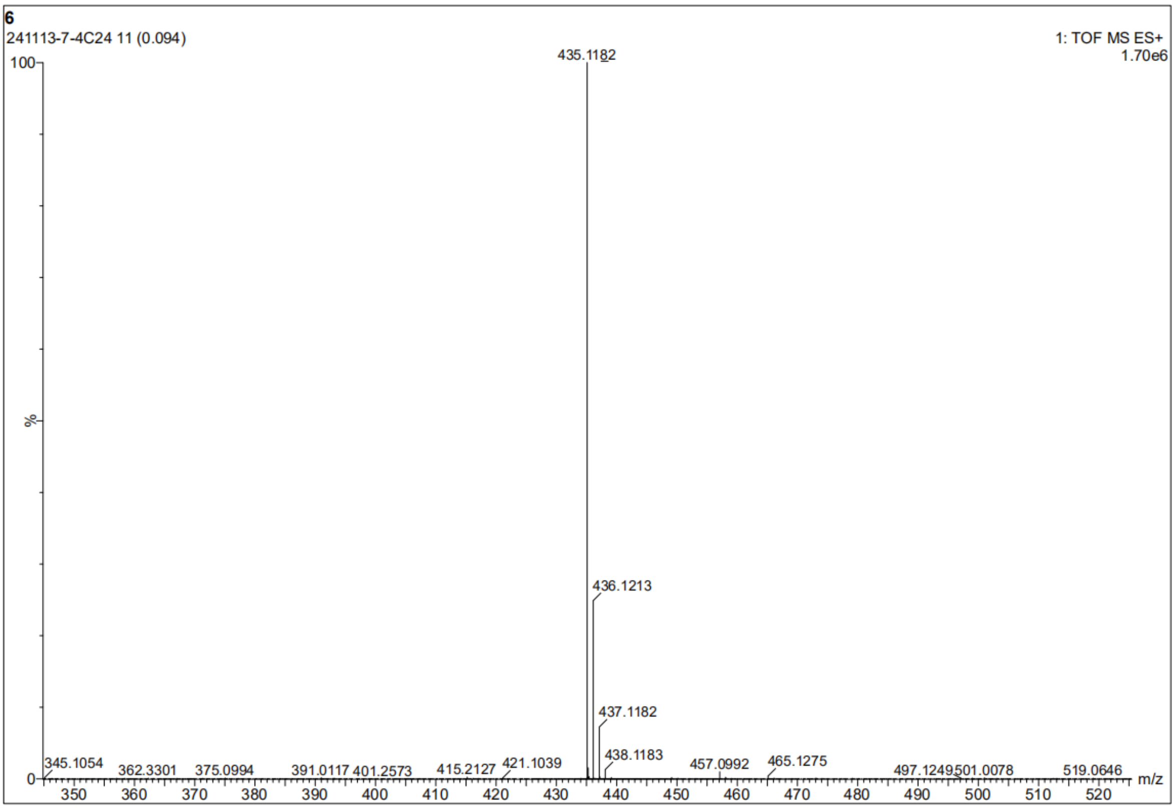

Fig.S47. Mass spectrum of compound 4c24

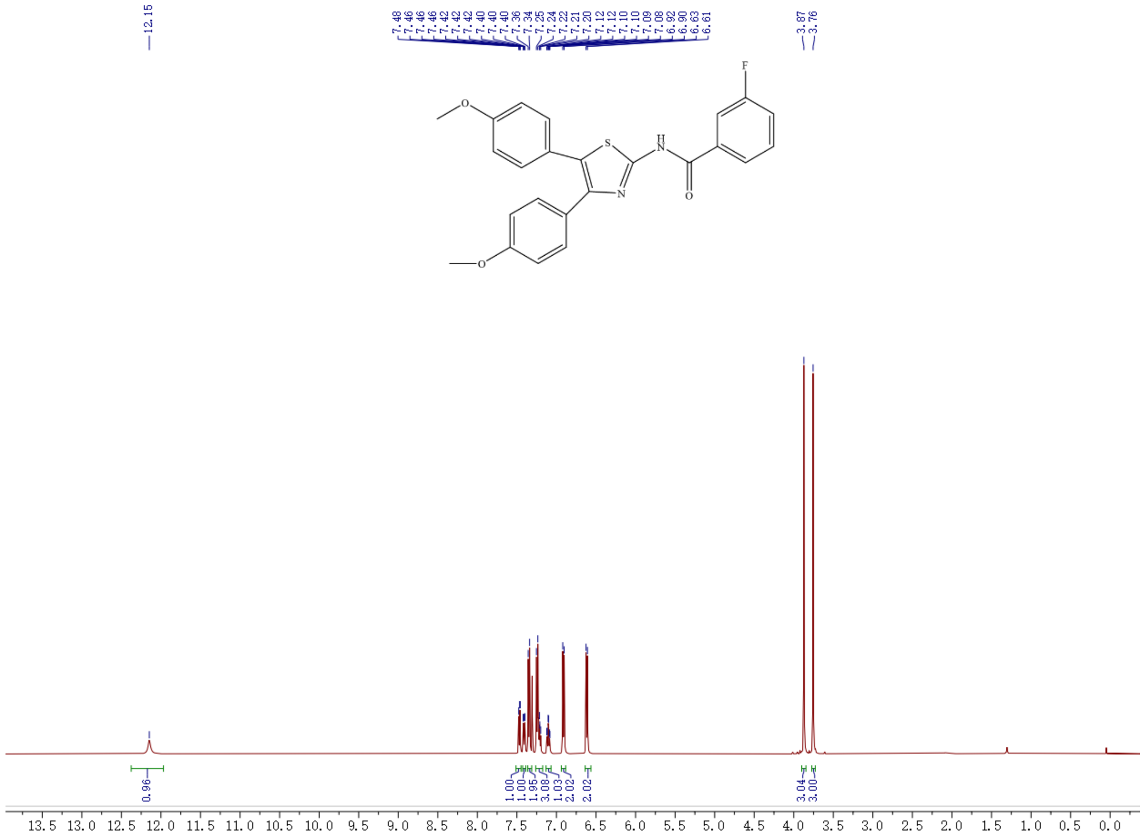

12.15
7.48
7.46
7.46
7.42
7.42
7.40
7.40
7.36
7.34
7.25
7.24
7.22
7.21
7.20
7.12
7.10
7.09
7.08
6.92
6.90
6.63
6.61
3.87
3.76
F
O
S
H
N
N
O
O
0.96
1.00
1.00
1.96
3.08
1.03
2.02
2.02
3.04
3.00
13.5 13.0 12.5 12.0 11.5 11.0 10.5 10.0 9.5 9.0 8.5 8.0 7.5 7.0 6.5 6.0 5.5 5.0 4.5 4.0 3.5 3.0 2.5 2.0 1.5 1.0 0.5 0.0

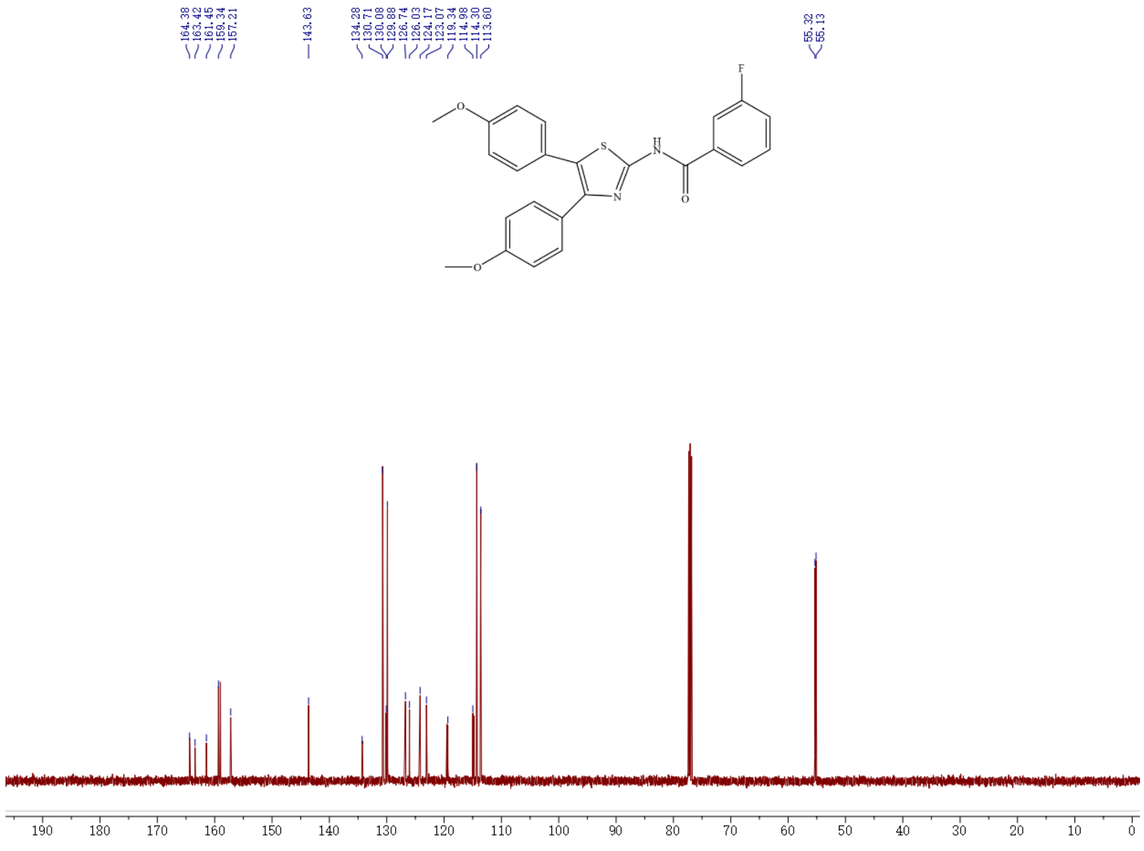

164.38
163.42
161.45
159.34
157.21
143.63
134.28
130.71
130.08
129.88
126.74
126.03
124.17
123.07
119.34
114.98
114.30
113.60
55.32
55.13
F
O
S
H
N
N
O
O
190 180 170 160 150 140 130 120 110 100 90 80 70 60 50 40 30 20 10 0

Fig.S48. 1H NMR And 13C NMR Spectrum of compound 4c24

N-(4,5-Bis(4-methoxyphenyl)thiazol-2-yl)-4-fluorobenzamide (4c25)

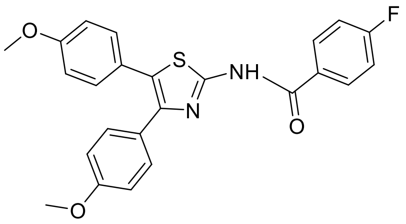

4c25, yield, 68%, white solid, m.p. 132.6-134.6℃. **^{1}H NMR** (500 MHz, $CDCl_3$) δ 12.02 (s, 1H), 7.74 (dd, J = 8.7, 5.3 Hz, 2H), 7.34 (d, J = 8.7 Hz, 2H), 7.25 (d, J = 8.7 Hz, 2H), 6.93 (dd, J = 14.4, 8.6 Hz, 4H), 6.63 (d, J = 8.7 Hz, 2H), 3.87 (s, 3H), 3.76 (s, 3H).**^{13}C NMR** δ 166.2, 164.5, 164.1, 159.2 (d, J = 43.3 Hz), 157.3, 143.6, 130.7, 130.1 (d, J = 9.3 Hz), 129.9, 128.2 (d, J = 3.0 Hz), 126.8, 125.9, 124.2, 115.6 (d, J = 22.1 Hz), 114.3, 113.6, 55.3, 55.2. HRMS, cald. ($C_{24}H_{19}FN_2O_3S$+H) = 435.1179, Found, 435.1182.

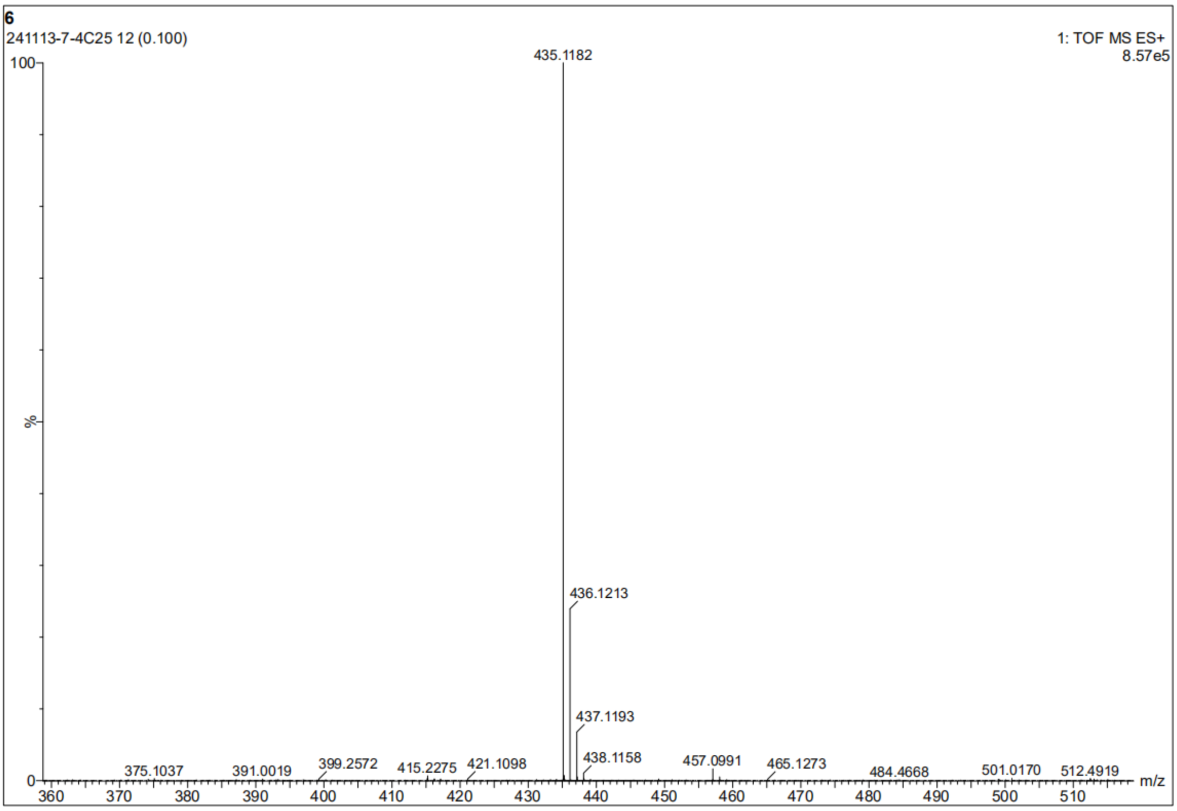

Fig.S49. Mass spectrum of compound 4c25

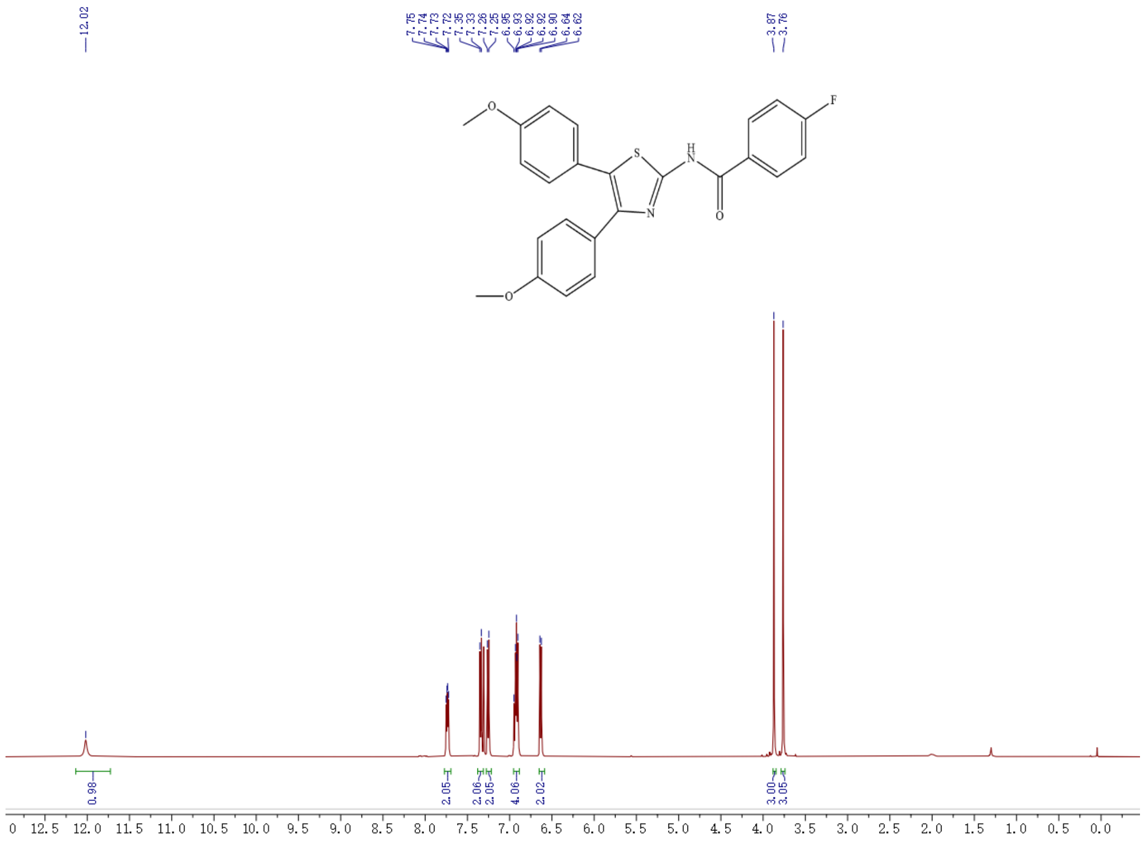

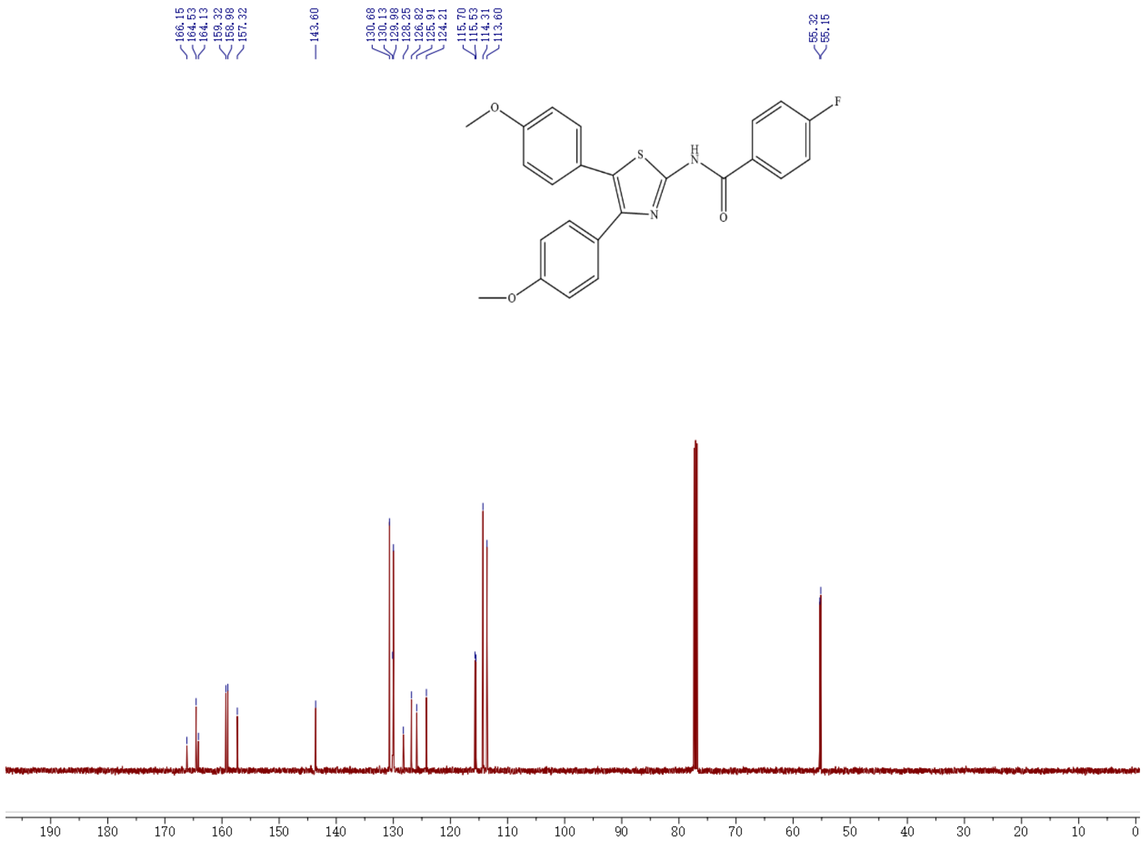

Fig.S50. 1H NMR And 13C NMR Spectrum of compound 4c25

N-(4,5-Bis(4-methoxyphenyl)thiazol-2-yl)-3-chlorobenzamide(4c26)

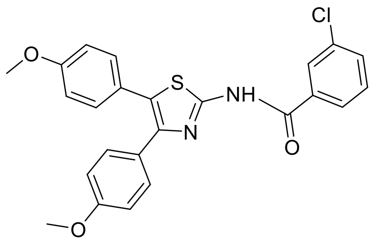

4c26, yield, 66%, white solid, m.p. 194.1-196.9°C. **^{1}H NMR** (500 MHz, $CDCl_3$) δ 12.46 (s, 1H), 7.64 (s, 1H), 7.55 (d, J = 7.8 Hz, 1H), 7.35 (t, J = 7.9 Hz, 3H), 7.23 (d, J = 8.7 Hz, 2H), 7.15 (t, J = 7.9 Hz, 1H), 6.92 (d, J = 8.7 Hz, 2H), 6.59 (d, J = 8.8 Hz, 2H), 3.87 (s, 3H), 3.75 (s, 3H).**^{13}C NMR** (125 MHz, $CDCl_3$) δ 164.5, 159.4, 159.0, 157.4, 143.5, 134.6, 133.9, 132.3, 130.7, 129.8, 129.7, 128.0, 126.6, 126.0, 125.6, 124.2, 114.3, 113.5, 55.3, 55.2.HRMS, cald. ($C_{24}H_{19}ClN_2O_3S$+H) = 451.0883, Found, 451.0886.

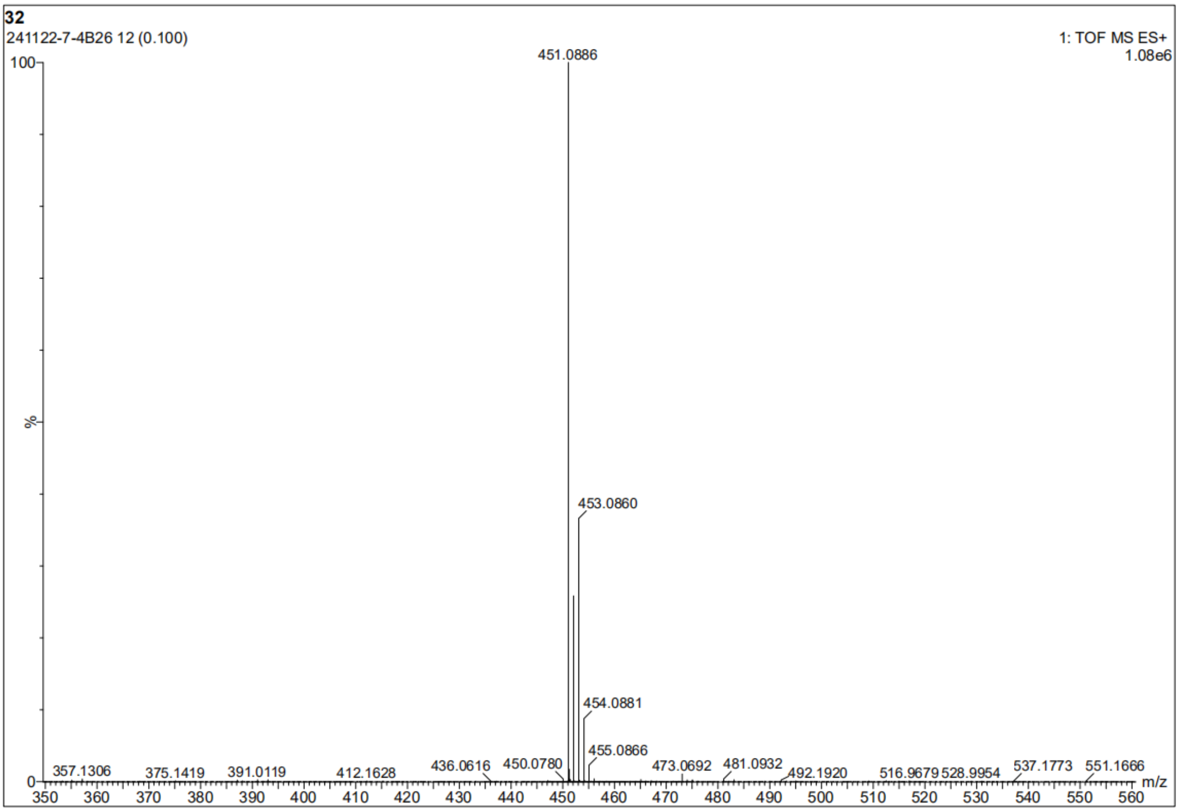

Fig.S51. Mass spectrum of compound 4c26

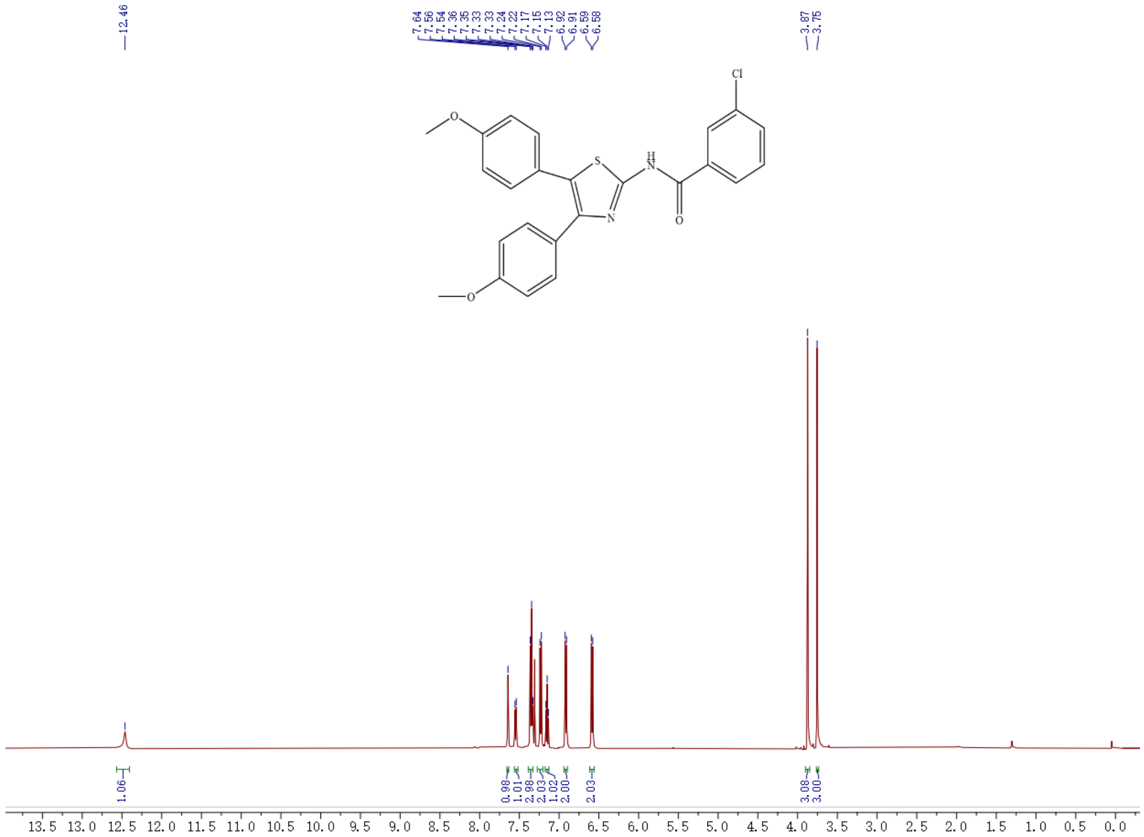
12.46
7.64 7.56 7.54 7.36 7.35 7.33 7.24 7.22 7.17 7.15 7.13 6.92 6.91 6.59 6.58
3.87 3.75
Cl
O
S
H
N
N
O
O
1.06
0.98 1.01 2.98 2.03 1.02 2.00 2.03
3.08 3.00
13.5 13.0 12.5 12.0 11.5 11.0 10.5 10.0 9.5 9.0 8.5 8.0 7.5 7.0 6.5 6.0 5.5 5.0 4.5 4.0 3.5 3.0 2.5 2.0 1.5 1.0 0.5 0.0

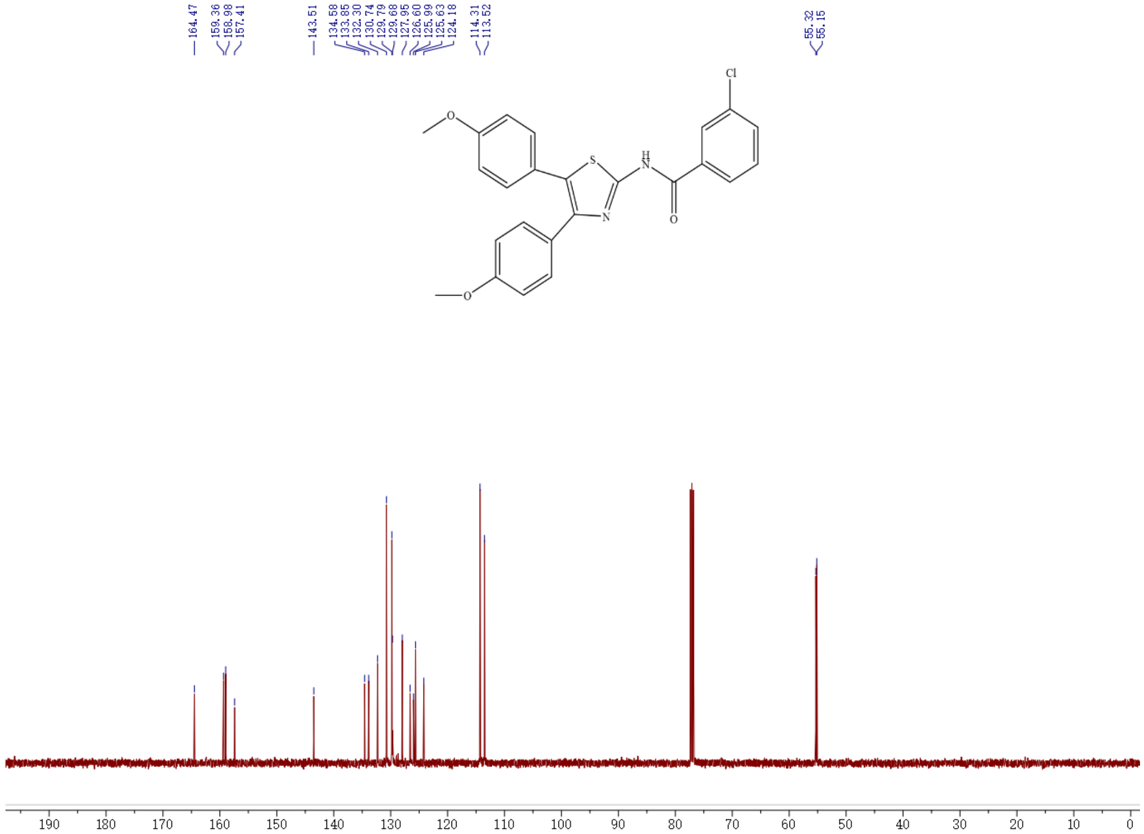
164.47
159.36 158.98 157.41
143.51
134.58 133.85 132.30 130.74 129.79 129.68 127.95 126.60 125.99 125.63 124.18
114.31 113.52
55.32 55.15
Cl
O
S
H
N
N
O
O
190 180 170 160 150 140 130 120 110 100 90 80 70 60 50 40 30 20 10 0

Fig.S52. 1H NMR And 13C NMR Spectrum of compound 4c26

N-(4,5-Bis(4-methoxyphenyl)thiazol-2-yl)-4-chlorobenzamide (4c27)

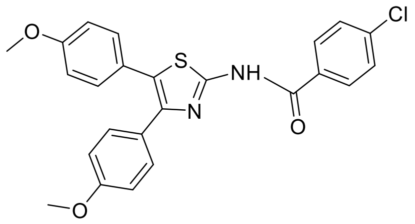

4c27, yield, 66%, white solid, m.p. 130.5-131.4℃. **^{1}H NMR** (500 MHz, $CDCl_3$) *δ* 12.21 (s, 1H), 7.63 (d, *J* = 8.5 Hz, 2H), 7.34 (d, *J* = 8.7 Hz, 2H), 7.21 (d, *J* = 3.6 Hz, 4H), 6.91 (d, *J* = 8.7 Hz, 2H), 6.62 (d, *J* = 8.7 Hz, 2H), 3.87 (s, 3H), 3.77 (s, 3H).**^{13}C NMR** (125 MHz, $CDCl_3$) δ 164.7, 159.3, 158.9, 157.4, 143.6, 138.7, 130.7, 130.4, 130.0, 129.0, 128.7, 126.7, 126.0, 124.2, 114.3, 113.6, 55.3, 55.2. HRMS, cald. ($C_{24}H_{19}ClN_2O_3S$+H) = 451.0883, Found, 451.0887.

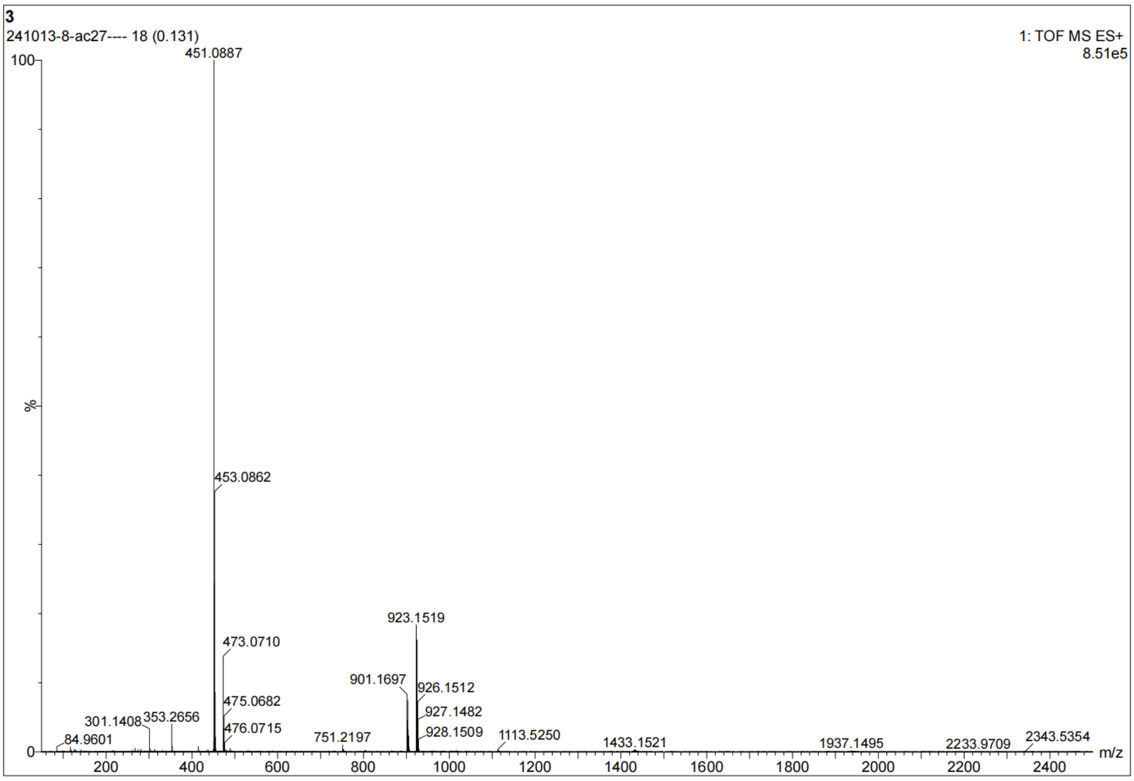

Fig.S53. Mass spectrum of compound 4c27

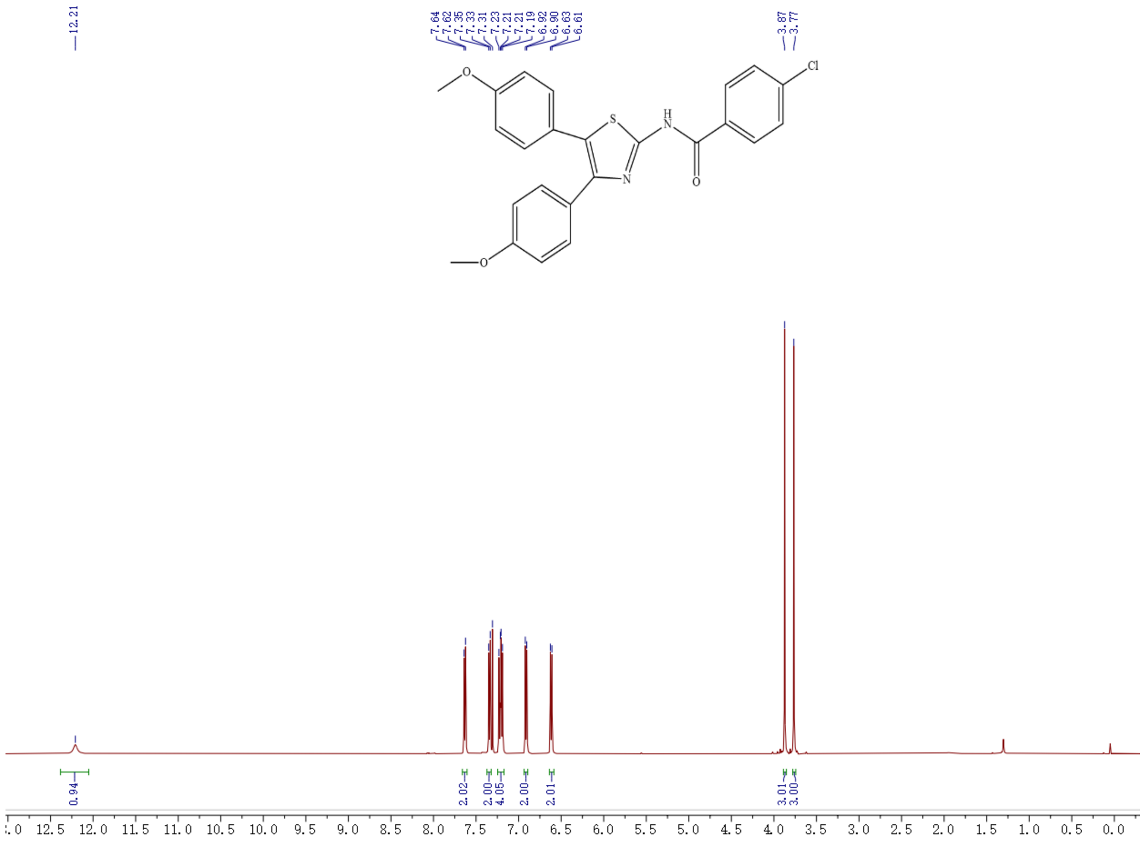

12.21
7.64
7.62
7.35
7.33
7.31
7.23
7.21
7.21
7.19
6.92
6.90
6.63
6.61
3.87
3.77
O
S
H
N
Cl
N
O
O
0.94
2.02
2.00
4.05
2.00
2.01
3.01
3.00
12.5
12.0
11.5
11.0
10.5
10.0
9.5
9.0
8.5
8.0
7.5
7.0
6.5
6.0
5.5
5.0
4.5
4.0
3.5
3.0
2.5
2.0
1.5
1.0
0.5
0.0

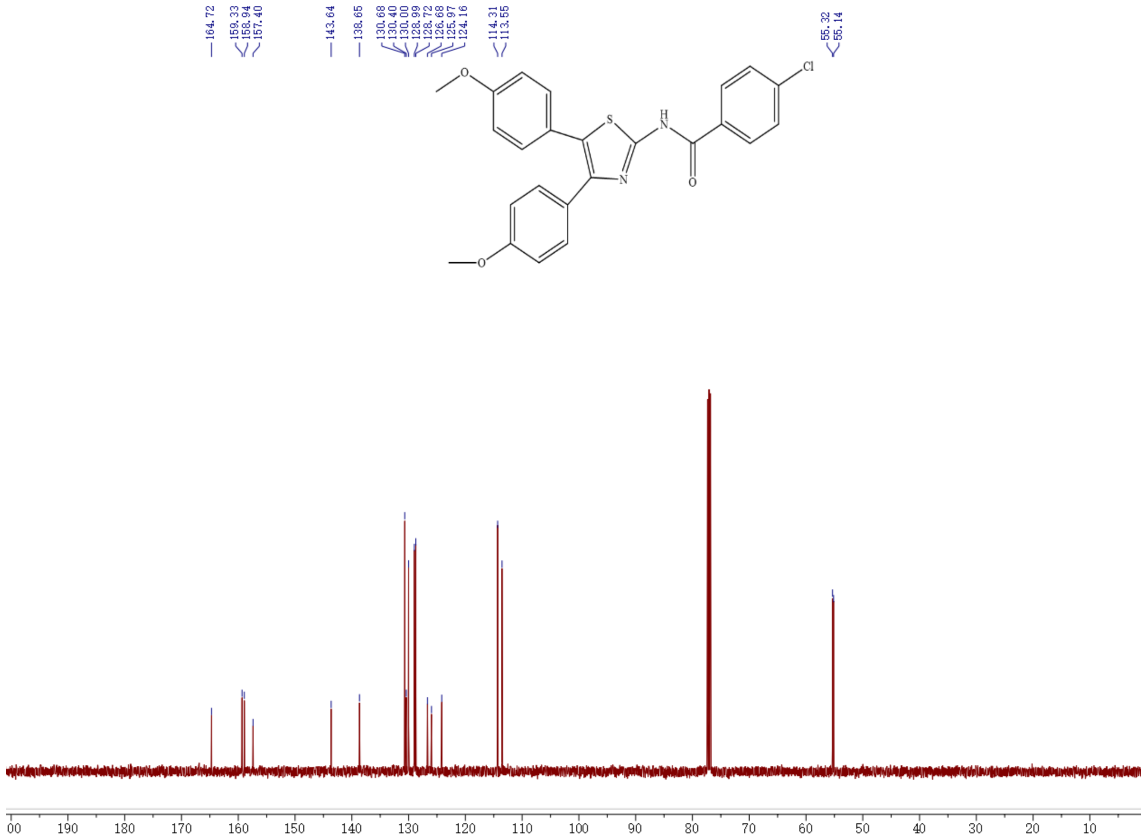

Fig.S54. 1H NMR And 13C NMR Spectrum of compound 4c27

N-(4,5-Bis(4-methoxyphenyl)thiazol-2-yl)-3-(trifluoromethyl)benzamide (4c28)

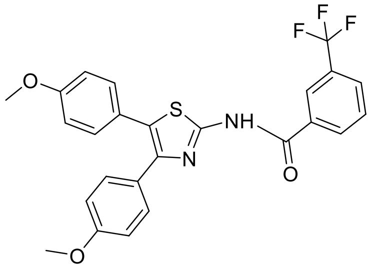

4c28, yield, 64%, white solid, m.p. 228.3-229.5℃. ^{1}**H NMR** (500 MHz, $CDCl_3$) δ 12.63 (s, 1H), 7.79 (d, J = 8.1 Hz, 2H), 7.48 (d, J = 8.2 Hz, 2H), 7.34 (d, J = 8.6 Hz, 2H), 7.18 (d, J = 8.7 Hz, 2H), 6.92 (d, J = 8.7 Hz, 2H), 6.55 (d, J = 8.7 Hz, 2H), 3.88 (s, 3H), 3.73 (s, 3H). ^{13}**C NMR** (125 MHz, $CDCl_3$) δ 164.5, 159.4, 158.9, 157.6, 143.4, 132.9, 131.0, 130.7 (d, J = 7.4 Hz), 130.5, 129.8, 129.1, 128.8 (q, J = 3.8 Hz), 126.3 (d, J = 31.9 Hz), 124.8 (d, J = 4.0 Hz), 124.6, 124.0, 122.4, 114.3, 113.5, 55.3, 55.0. HRMS, cald. ($C_{25}H_{19}F_3N_2O_3S$+H) = 485.1147, Found, 485.1152.

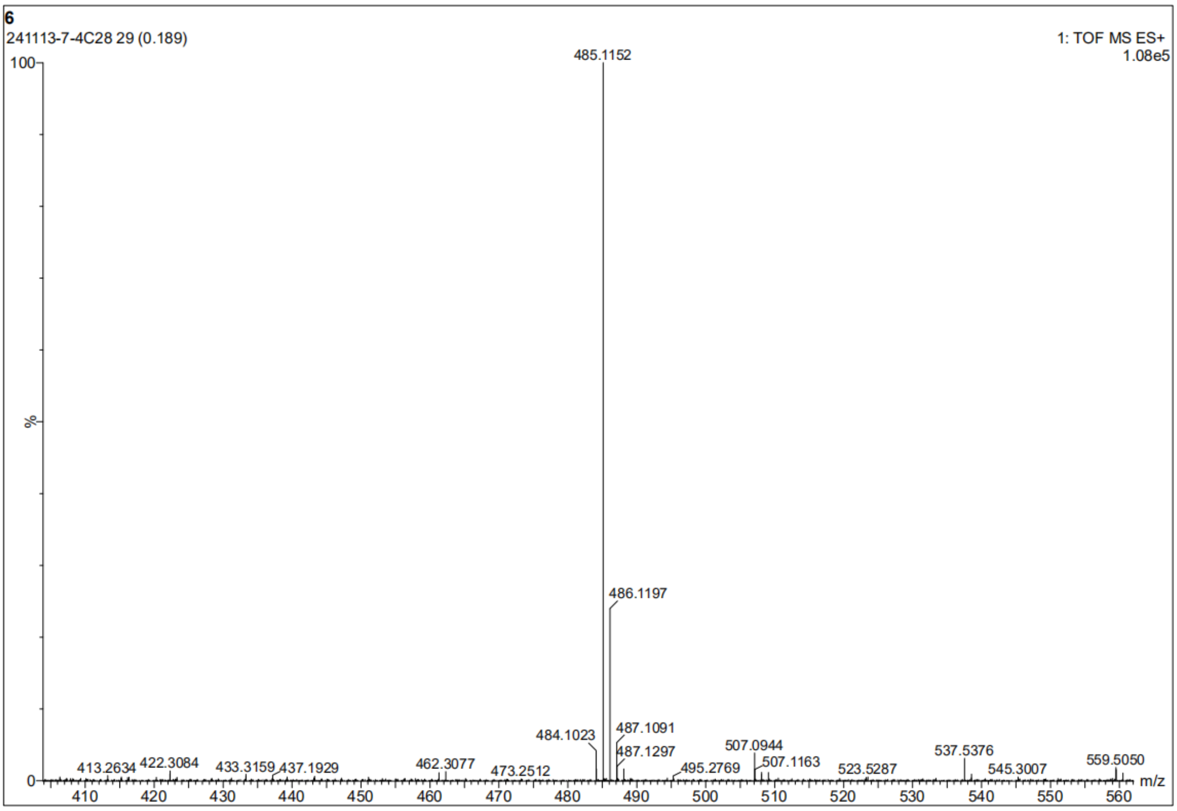

Fig.S55. Mass spectrum of compound 4c28

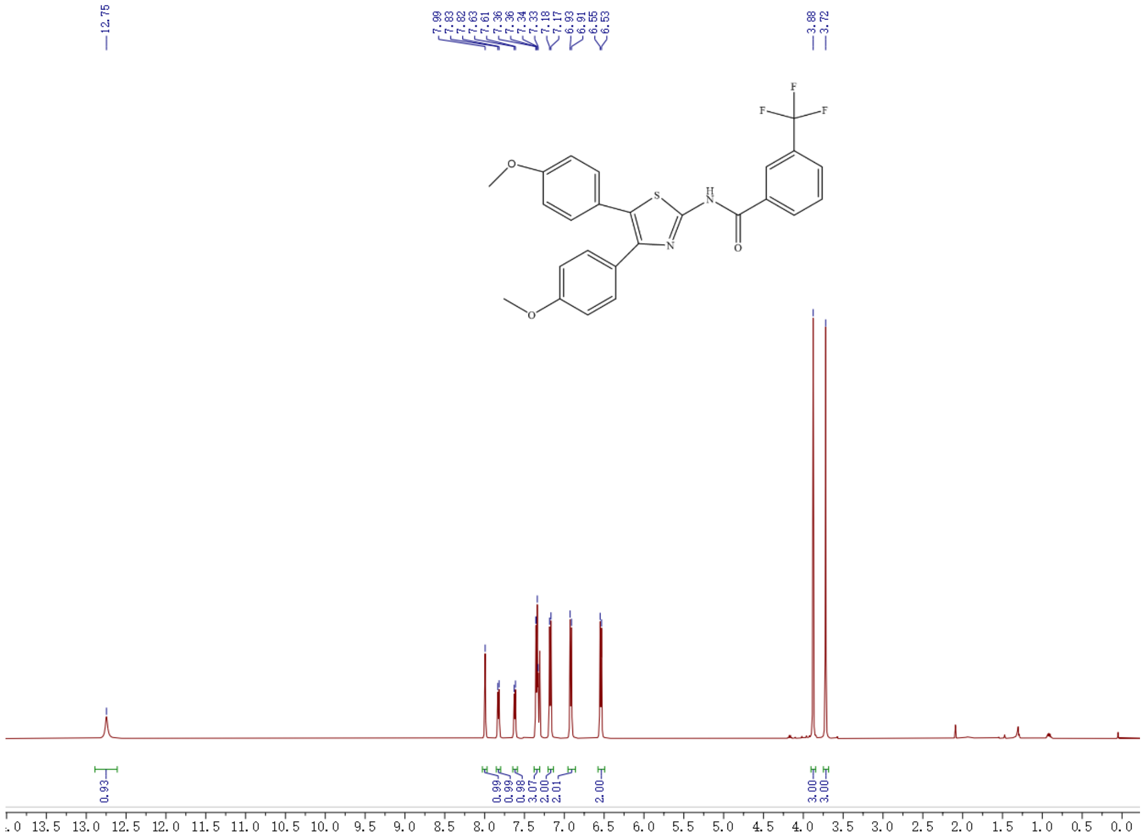
12.75
7.99
7.83
7.82
7.63
7.61
7.36
7.36
7.34
7.33
7.18
7.17
6.93
6.91
6.55
6.53
3.88
3.72
F
F
F
O
S
H
N
N
O
O
0.93
0.99
0.99
0.98
3.07
2.00
2.01
2.00
3.00
3.00
13.5 13.0 12.5 12.0 11.5 11.0 10.5 10.0 9.5 9.0 8.5 8.0 7.5 7.0 6.5 6.0 5.5 5.0 4.5 4.0 3.5 3.0 2.5 2.0 1.5 1.0 0.5 0.0

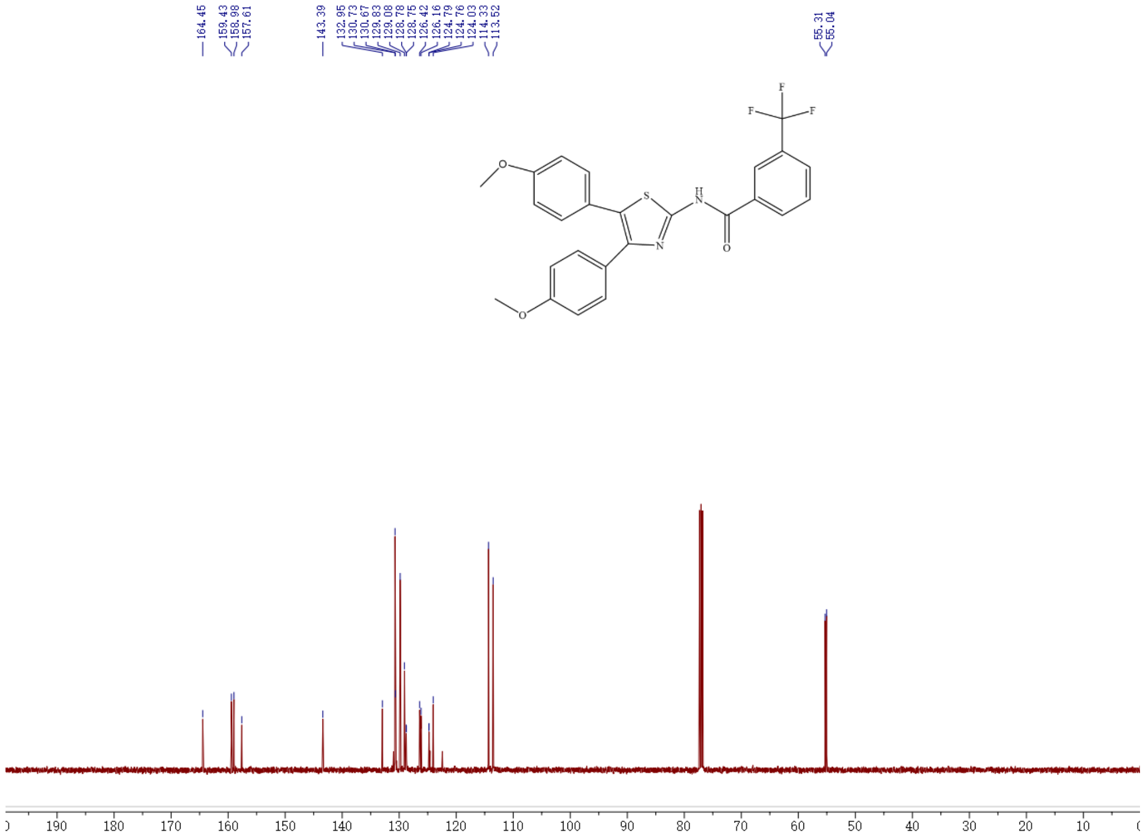
164.45
159.43
158.98
157.61
143.39
132.95
130.73
130.67
129.83
129.08
128.78
128.75
126.42
126.16
124.79
124.76
124.03
114.33
113.52
55.31
55.04
F
F
F
O
S
H
N
N
O
O
190 180 170 160 150 140 130 120 110 100 90 80 70 60 50 40 30 20 10

Fig.S56. 1H NMR And 13C NMR Spectrum of compound 4c28

***N*-[4,5-bis(4-methoxyphenyl)-1,3-thiazol-2-yl]-4-(trifluoromethyl)benzamide (4c29)**

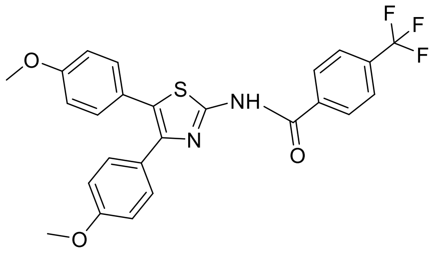

4c29, yield, 64%, white solid, m.p. 175.3-181.5℃. **^{1}H NMR** (500 MHz, $CDCl_3$) δ 12.75 (s, 1H), 7.99 (s, 1H), 7.83 (d, J = 7.7 Hz, 1H), 7.62 (d, J = 7.7 Hz, 1H), 7.35 (d, J = 8.6 Hz, 3H), 7.18 (d, J = 8.7 Hz, 2H), 6.92 (d, J = 8.6 Hz, 2H), 6.54 (d, J = 8.7 Hz, 2H), 3.88 (s, 3H), 3.72 (s, 3H).**^{13}C NMR** (125 MHz, $CDCl_3$) δ 164.5, 159.4, 158.9, 157.5, 143.6, 135.2, 133.5 (d, J = 32.6 Hz), 130.6, 129.9, 128.1, 126.3 (d, J = 32.6 Hz), 125.3 (q, J = 32.6 Hz), 124.6, 123.9, 122.4, 114.3, 113.6, 55.3, 55.0. HRMS, cald. ($C_{25}H_{19}F_3N_2O_3S$+H) = 485.1147, Found, 485.1150.

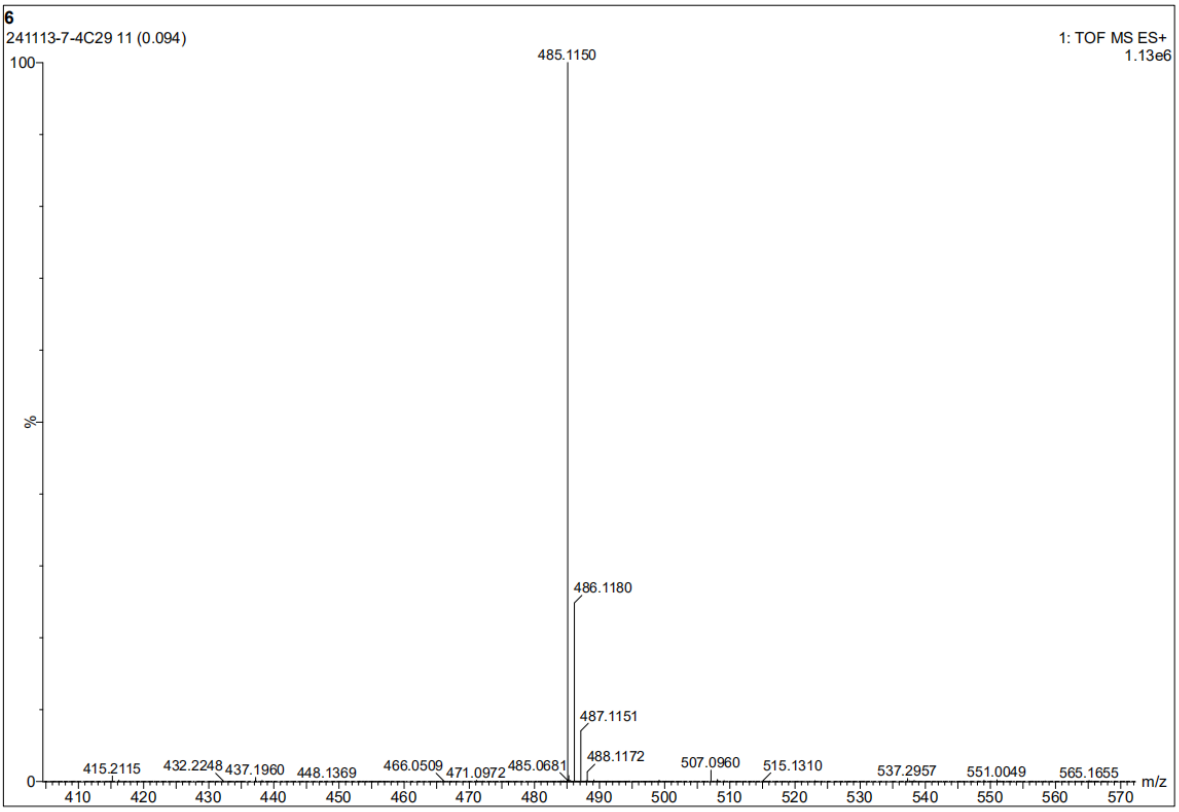

Fig.S57. Mass spectrum of compound 4c29

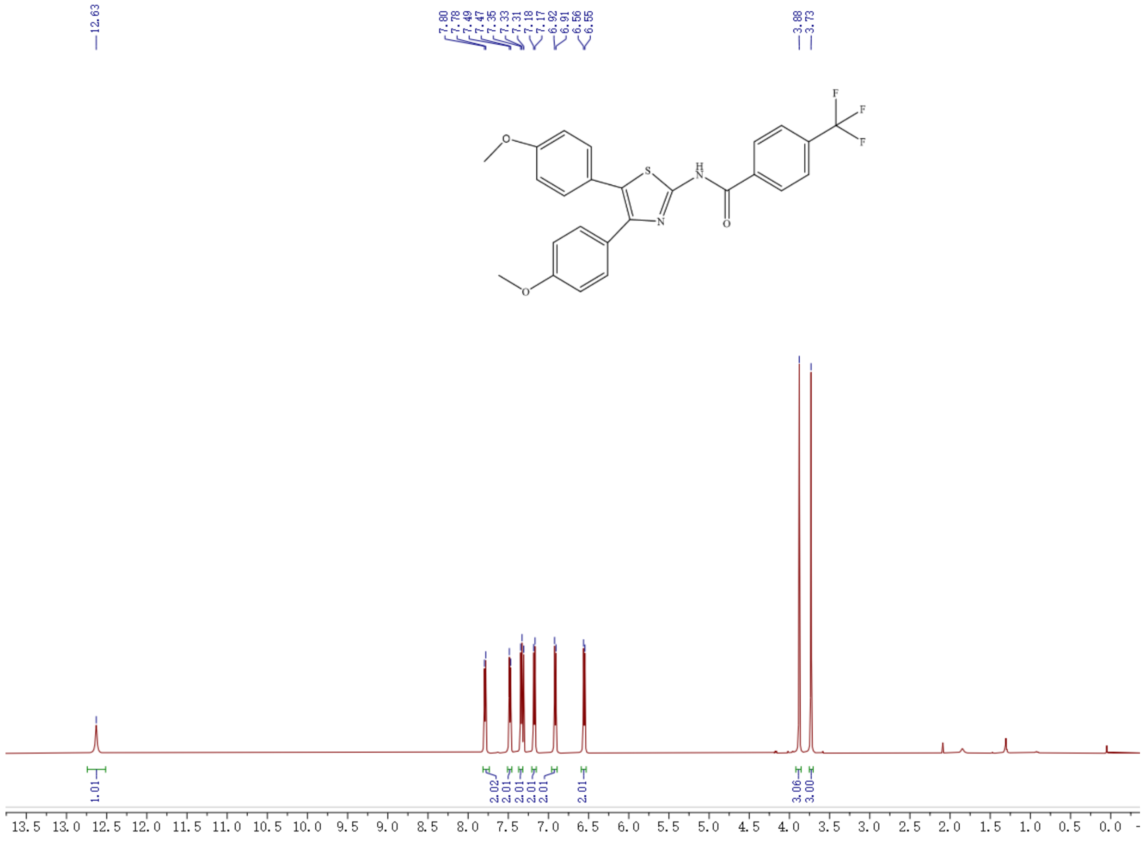

12.63
7.80
7.78
7.49
7.47
7.35
7.33
7.31
7.18
7.17
6.92
6.91
6.56
6.55
3.88
3.73
1.01
2.02
2.01
2.01
2.01
2.01
2.01
3.06
3.00
13.5 13.0 12.5 12.0 11.5 11.0 10.5 10.0 9.5 9.0 8.5 8.0 7.5 7.0 6.5 6.0 5.5 5.0 4.5 4.0 3.5 3.0 2.5 2.0 1.5 1.0 0.5 0.0

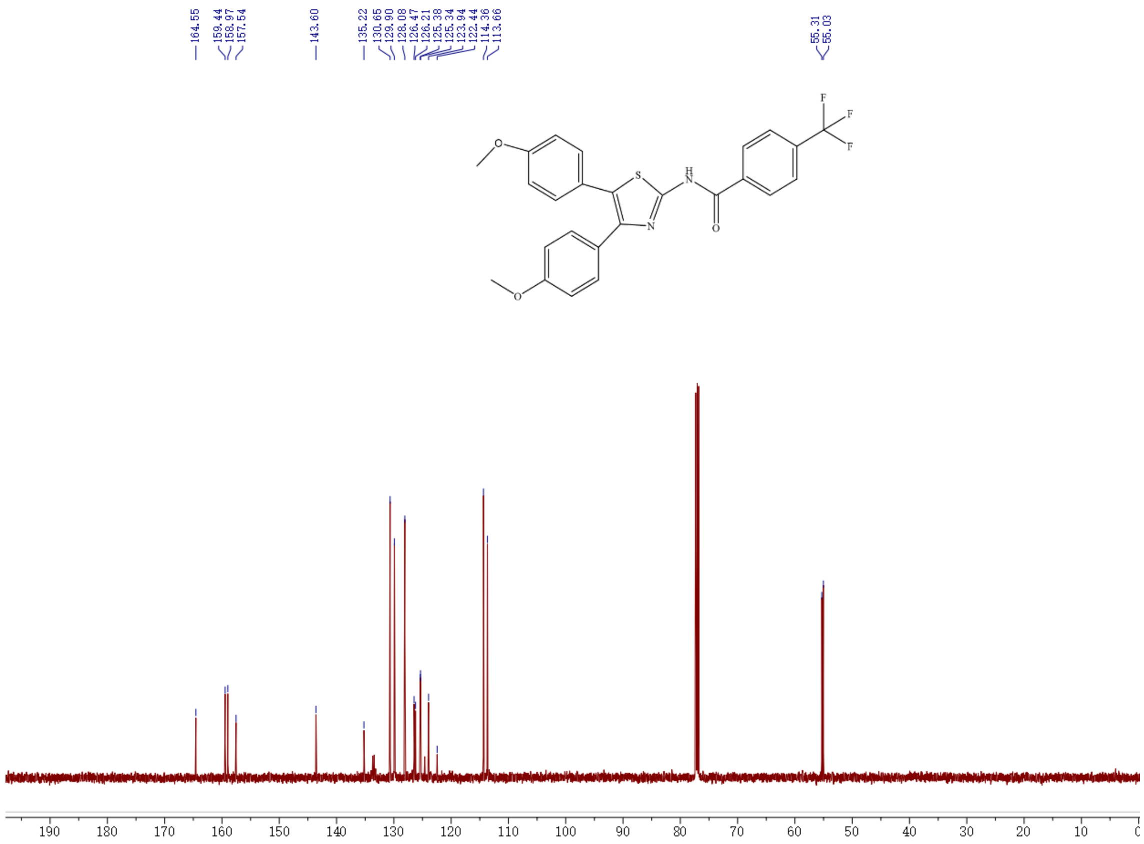

Fig.S58. 1H NMR And 13C NMR Spectrum of compound 4c29

***N*-[4-(3,4-dihydroxyphenyl)-5-phenyl-1,3-thiazol-2-yl]-4-methylbenzamide (5a8)**

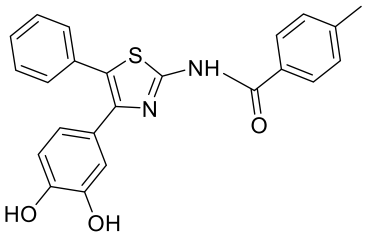

5a8, yield, 70%, yellowish solid, m.p. 123.6-125.5℃. **^{1}H NMR** (400 MHz, DMSO-*d*$_6$) δ 12.71 (s, 1H), 8.04 (d, *J* = 8.0 Hz, 2H), 7.53 – 7.26 (m, 9H), 6.92 (s, 1H), 6.73 (d, *J* = 8.7 Hz, 1H), 6.67 (d, *J* = 8.2 Hz, 1H), 2.40 (s, 3H). **^{13}C NMR** (100 MHz, DMSO-*d6*) δ 165.6, 146.4, 145.7, 145.4, 143.3, 132.9, 129.7, 129.7, 129.3, 128.7, 128.4, 128.0, 123.8, 120.6, 116.8, 115.9, 115.4, 109.9, 21.6. HRMS, cald. ($C_{23}H_{18}N_2O_3S$+H) = 403.1116, Found, 403.1119.

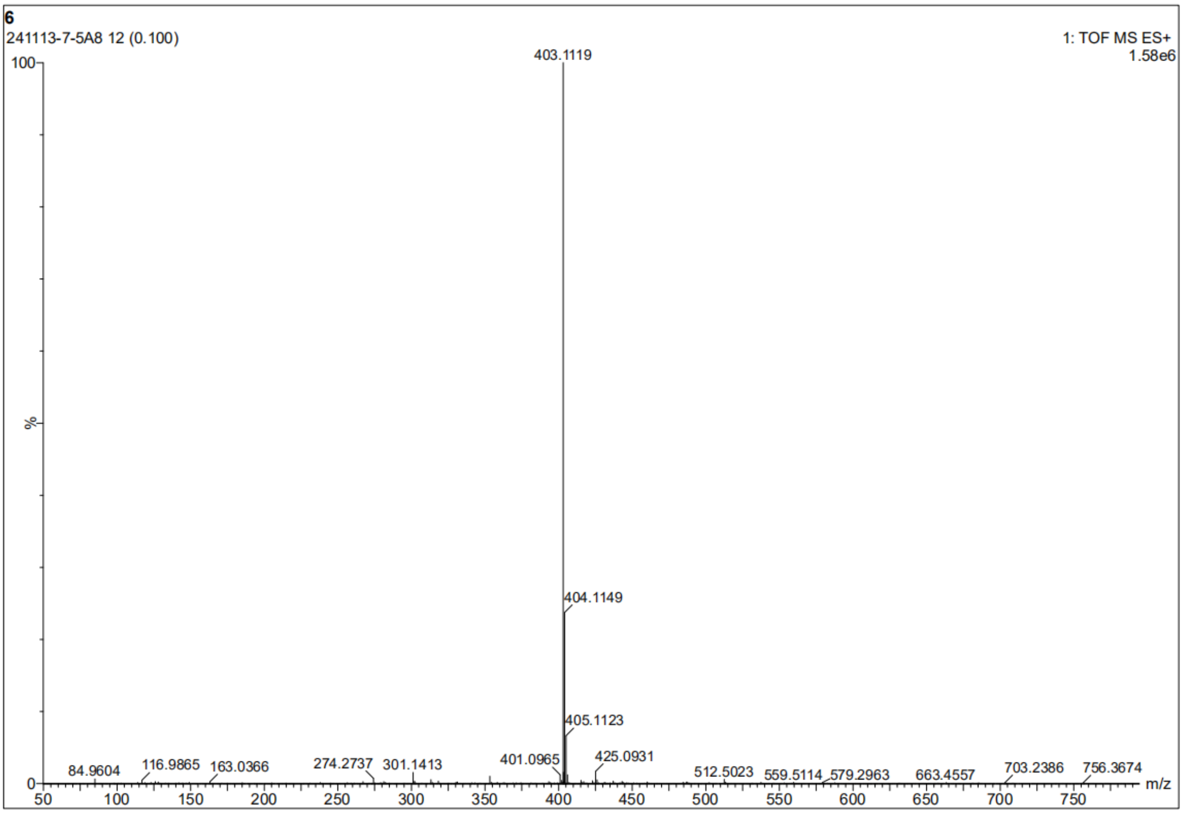

Fig.S59. Mass spectrum of compound 5a8

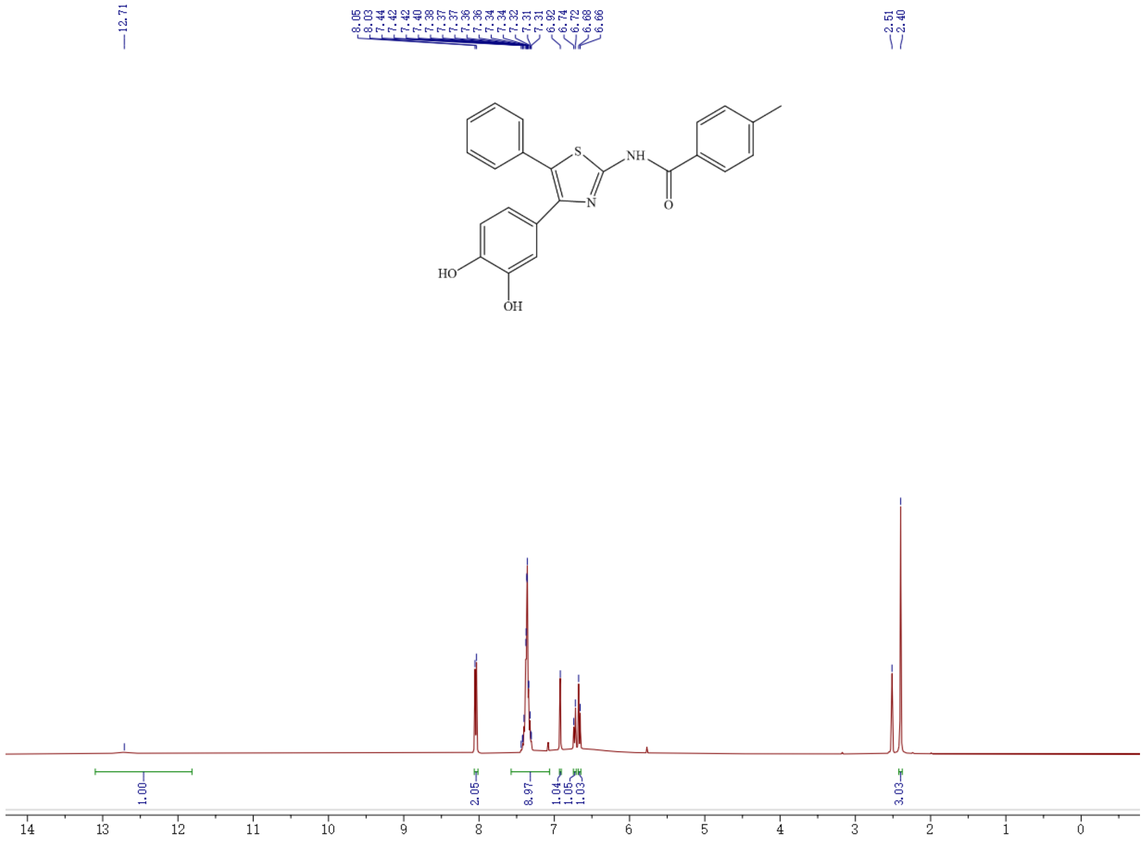

12.71
8.05
8.03
7.44
7.42
7.40
7.38
7.37
7.37
7.36
7.36
7.34
7.34
7.32
7.31
7.31
6.92
6.74
6.72
6.68
6.66
2.51
2.40
S
NH
N
O
HO
OH
1.00
2.05
8.97
1.04
1.05
1.03
3.03
14
13
12
11
10
9
8
7
6
5
4
3
2
1
0

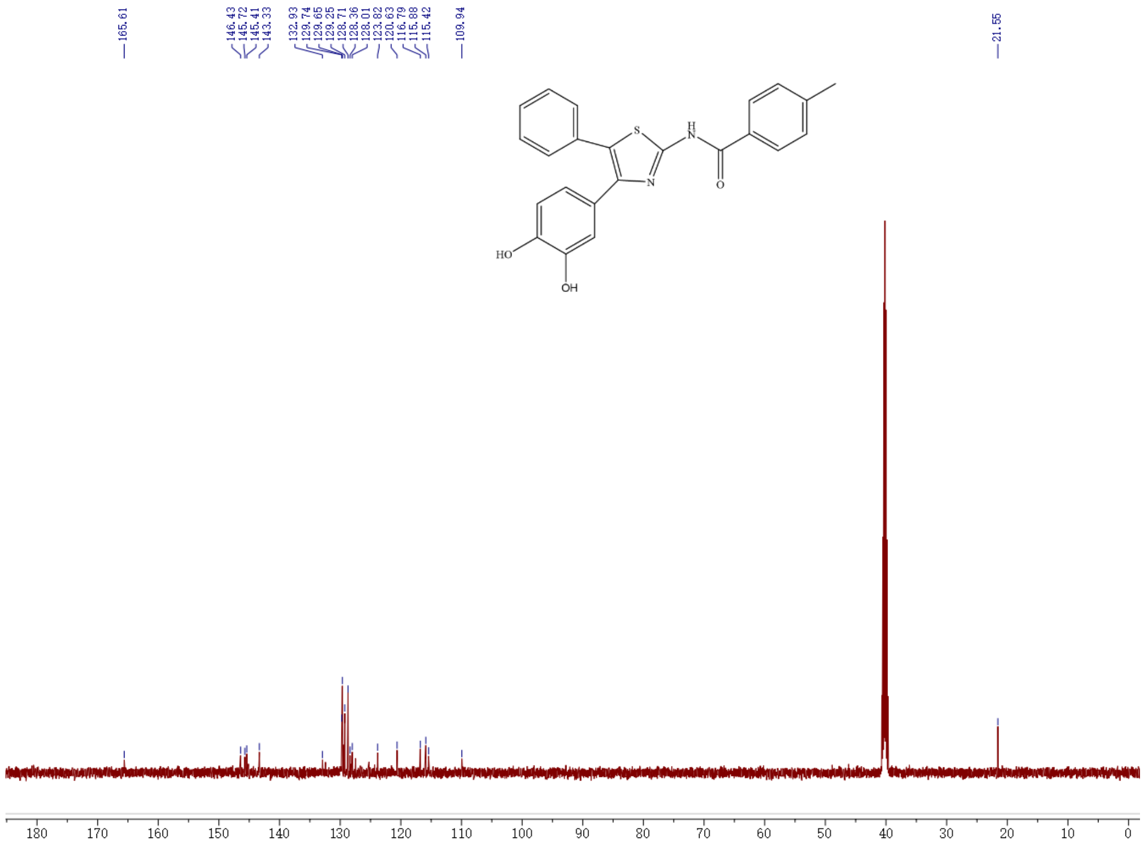

Fig.S60. 1H NMR And 13C NMR Spectrum of compound 5a8

4-(3,4-dimethoxyphenyl)-5-phenylthiazol-2-amine (3a)

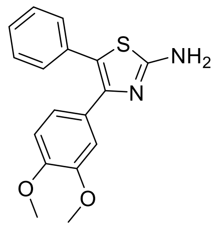

3a, yield,70%, white solid. **^{1}H NMR**(400 MHz, DMSO-*d6*) δ 7.29-7.23 (m, 7H), 6.96 (m, 2H), 6.84 (d, *J* = 8.1 Hz,1H), 3.72 (s, 3H), 3.52 (s, 3H). **^{13}C NMR** (101 MHz, DMSO-*d6*) δ 166.46, 148.64, 148.44, 144.57, 133.39, 129.64, 129.16, 127.95, 127.53, 121.43, 118.10, 112.53, 111.71, 55.83, 55.45.

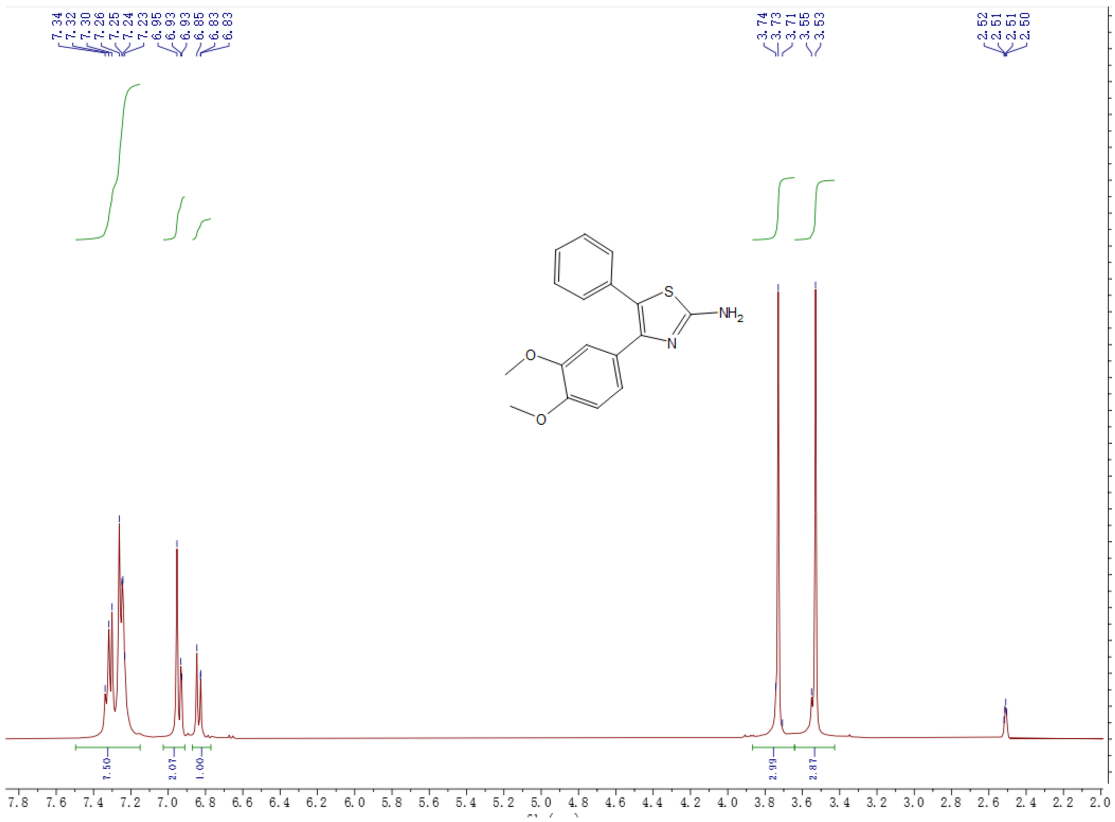

Fig.S61. 1H NMR   Spectrum of compound 3a

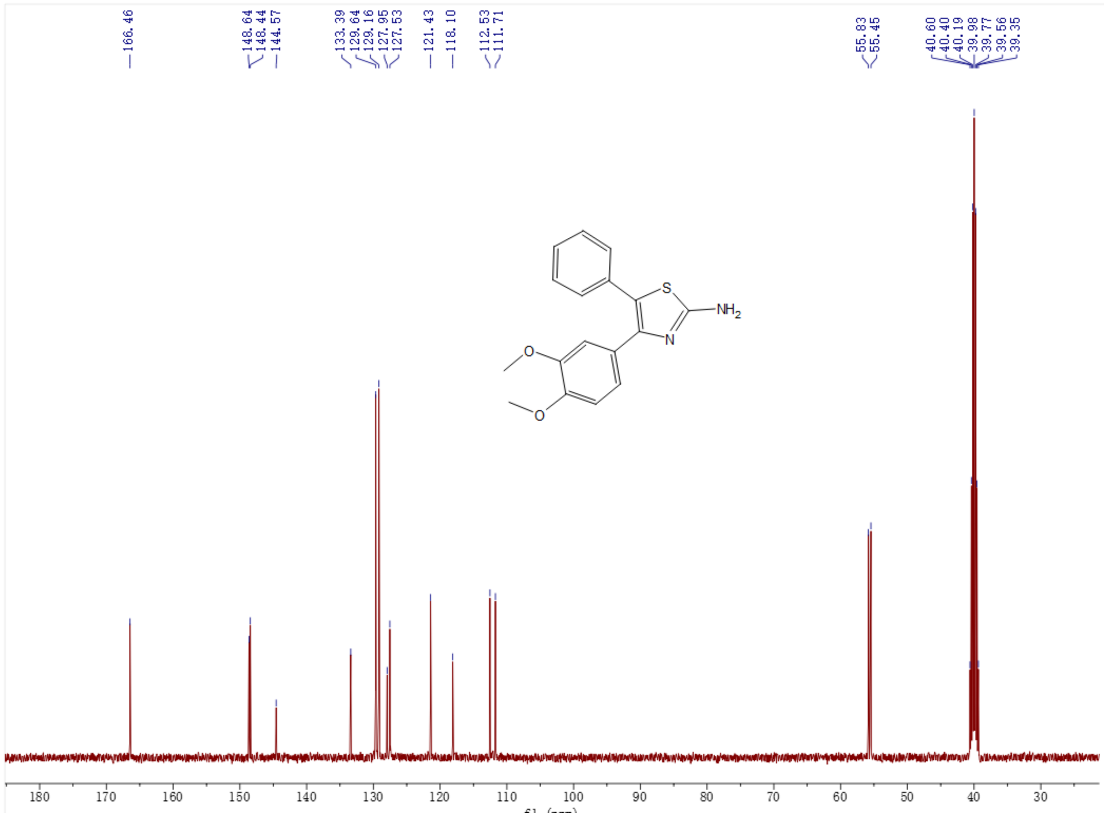

Fig.S62. 13C NMR Spectrum of compound 3a

4-(3-methoxyphenyl)-5-phenylthiazol-2-amine(3b)

3b, yield,72%, white solid. **^{1}H NMR**(400 MHz, DMSO-*d6*) δ 7.33 (d, *J* = 8.8 Hz, 2H), 7.15 (d, *J* = 8.7 Hz, 2H), 7.02 (s, 2H), 6.88 (d, *J* = 8.7 Hz, 2H), 6.81 (d, *J* = 8.9 Hz, 2H) 3.77 (s, 3H), 3.73 (s, 3H). **^{13}C NMR** (101 MHz, DMSO-*d6*) δ 166.38, 158.89, 145.19, 133.54, 130.15, 129.36, 129.18, 128.31, 127.33, 118.02, 113.91, 55.47.

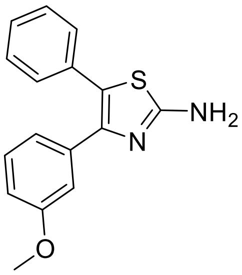

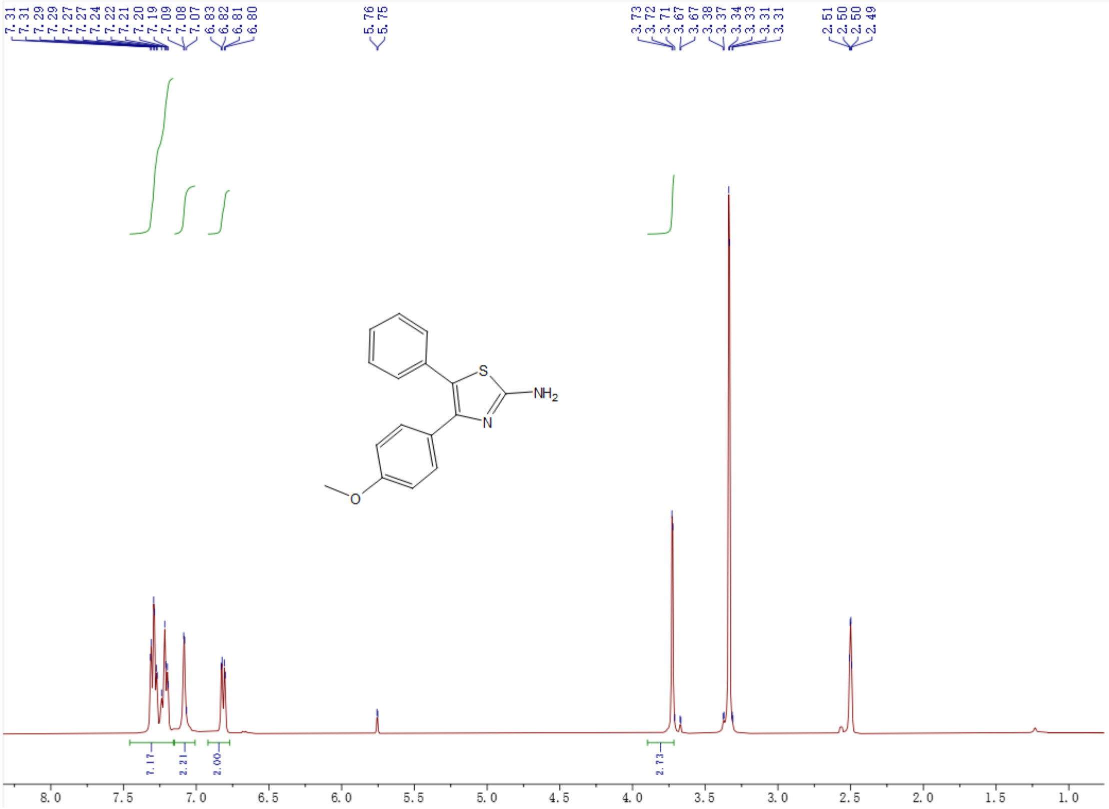

Fig.S63. 1H NMR Spectrum of compound 3b

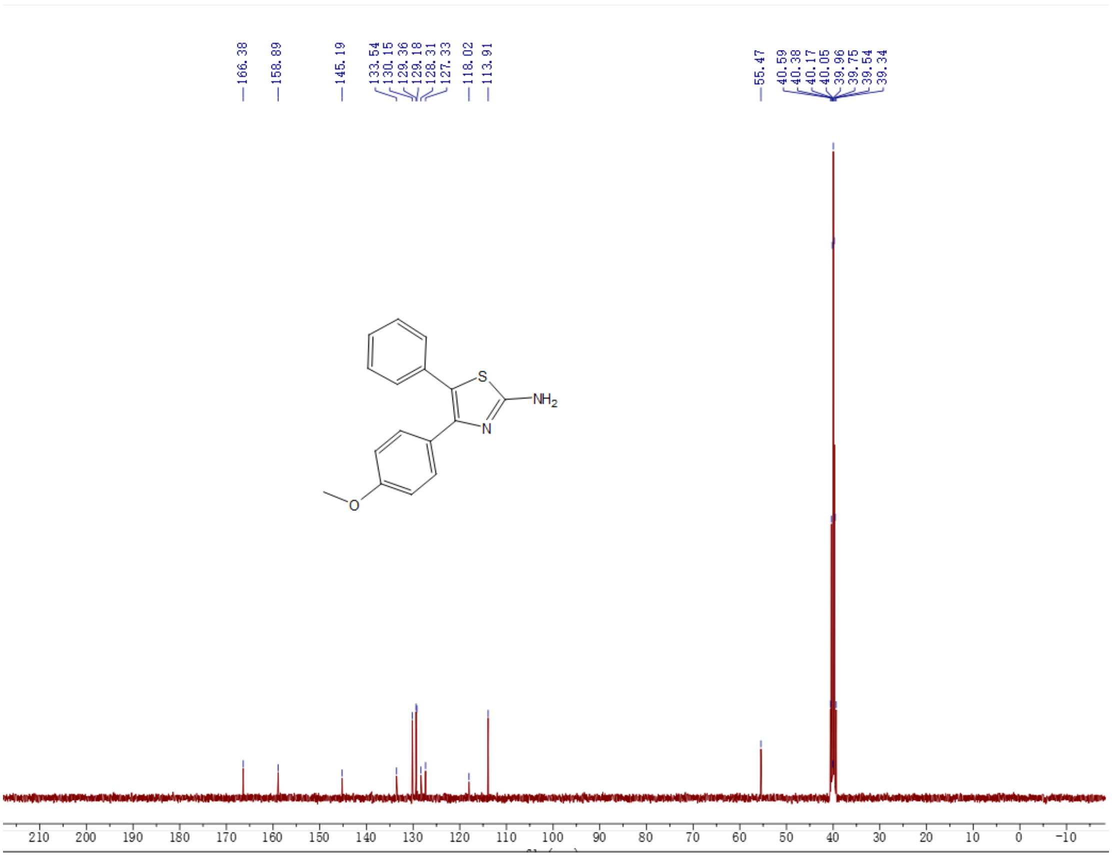

Fig.S64. 13C NMR Spectrum of compound 3b

4,5-bis(4-methoxyphenyl)thiazol-2-amine(3c)

3c, yield,70%, white solid. ^{1}H NMR (400 MHz, DMSO-d_6) δ 7.33 (d, J = 8.8 Hz, 2H), 7.15 (d, J = 8.7 Hz, 2H), 7.02 (s, 2H), 6.88 (d, J = 8.7 Hz, 2H), 6.81 (d, J = 8.9 Hz, 2H) 3.77 (s, 3H), 3.73 (s, 3H). ^{13}C NMR (101 MHz, DMSO-d_6) δ 165.93, 158.74, 158.73, 144.37, 130.81, 130.01, 128.46, 125.60, 118.04, 114.65, 113.86, 55.54, 55.44.

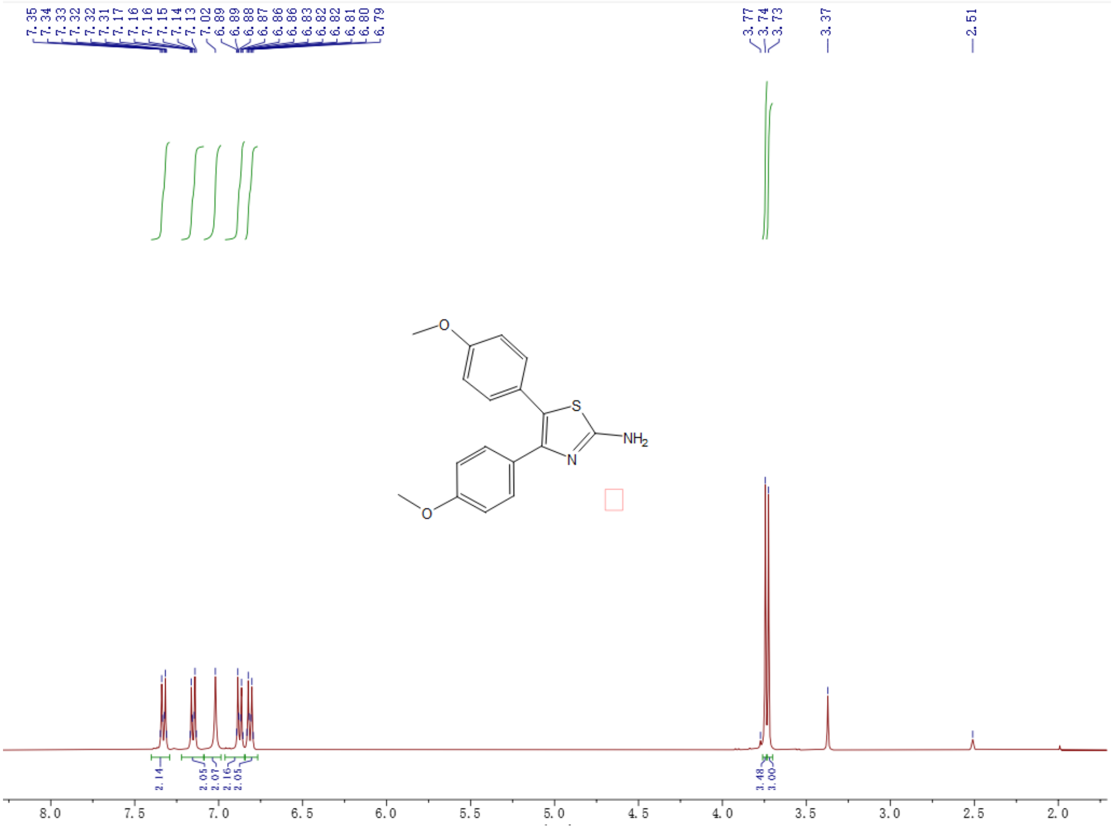

Fig.S65. 1H NMR Spectrum of compound 3c

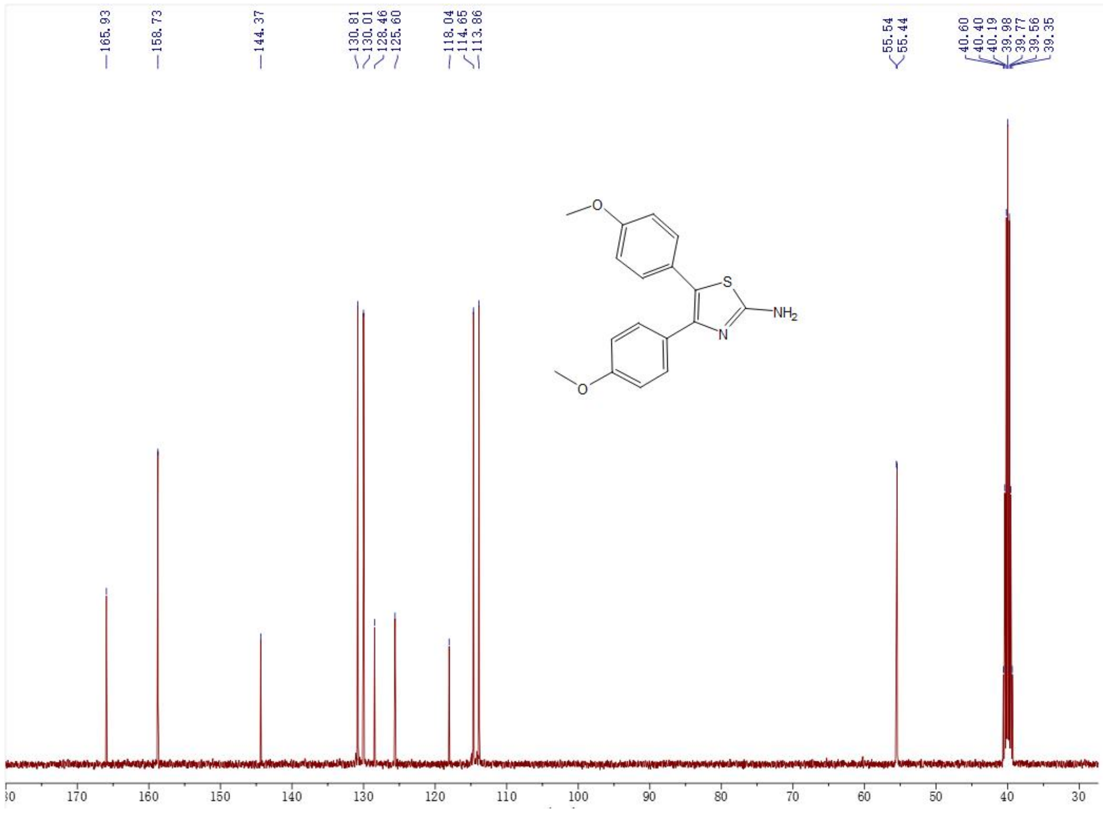

Fig.S66.   13C NMR Spectrum of compound 3c
